# Copper-catalyzed cyanothiolation to incorporate a sulfur-substituted quaternary carbon center†
†Electronic supplementary information (ESI) available. CCDC 1546232. ESI and crystallographic data in CIF or other electronic format. See DOI: 10.1039/c7sc02867a



**DOI:** 10.1039/c7sc02867a

**Published:** 2017-08-15

**Authors:** Yubing Huang, Xianwei Li, Xu Wang, Yue Yu, Jia Zheng, Wanqing Wu, Huanfeng Jiang

**Affiliations:** a Key Laboratory of Functional Molecular Engineering of Guangdong Province , School of Chemistry and Chemical Engineering , South China University of Technology , Guangzhou 510640 , China . Email: cewuwq@scut.edu.cn ; Email: jianghf@scut.edu.cn

## Abstract

Sulfur-containing nitriles have important research value in the life sciences due to their diverse biological activities resulting from the sulfur and cyano functional groups. Herein, a copper-catalyzed cyanothiolation of *N*-tosylhydrazones with thiocyanates to generate α-arylthioalkanenitriles bearing sulfur-substituted quaternary carbon center atoms has been described. This novel protocol involves the procedure of copper carbene species promoting S–CN bond cleavage and C–CN/C–S bond reconstruction to introduce both sulfur and cyano groups onto a single carbon center. This cyanothiolation reaction will greatly enhance the synthetic utility of carbenoid species as new entries for the construction of diverse heteroatom-containing nitriles *via* cyanofunctionalization of metal–carbene species.

## Abstract 2

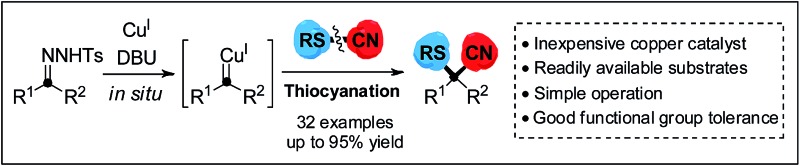
A copper-catalyzed cyanothiolation of *N*-tosylhydrazones with thiocyanates to generate α-arylthioalkanenitriles bearing sulfur-substituted quaternary carbon center atoms has been achieved.

## Introduction

**Scheme 1:**
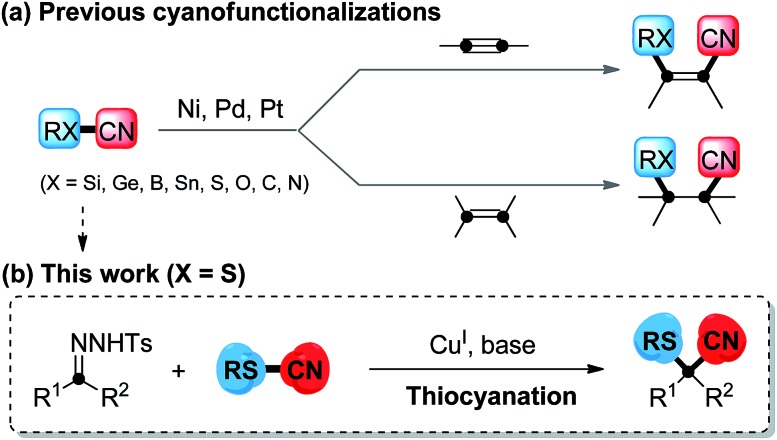
Transition metal-catalyzed cyanofunctionalization reactions.

Transition metal-catalyzed cyanofunctionalization reactions are direct and powerful strategies for the introduction of cyano groups and another functional groups simultaneously into one single molecular framework. It is known that cyano groups are often building blocks of pharmaceuticals,^1^ agrochemicals and functionalized materials, and are easily transformed into aldehydes, amines, amides or other carboxyl derivatives. Conceivably, the development of cyanofunctionalization is extremely desirable in the construction of functionalized nitriles,^2^ and it has caught increasing attention in the past decades. Previous cyanofunctionalizations generally required the cleavage of the RX–CN (X = Si,^3^ Ge,^4^ B,^5^ Sn,^6^ S,^7^ O,^8^ C,^9^ or N^10^) bond through oxidative addition of transition metal (M = Ni, Pd, Pt) complexes, and immediately, both functional fragments were simultaneously installed at disparate carbon atoms of versatile substrates, including arynes, alkenes and alkynes (Scheme 1a). However, a process to incorporate two cleaved functional fragments onto a single carbon atom *via* transition metal-catalyzed cyanofunctionalization has never been realized, and is still a challenging issue.

Carbenoid species,^11^ generated easily from diazo compounds, are a class of significant reactive intermediates, as they display both electrophilic and nucleophilic reactivity in a single carbon center.^11*g*^ In recent years, gem-difunctionalization of metal–carbene species has been of concern, as it provides direct pathways to enhance the synthetic applications of carbenoid species. In this respect, the chemical bond cleavage of heteroatom-containing reactants and installation of both cleaved carbon and heteroatom fragments onto carbenoid species *via* transition metal catalysis is one of the most attractive and promising methods to incorporate a heteroatom-containing quaternary carbon center. Currently, examples of inserting nitrogen^12^ and oxygen,^13^ which have small atomic radii, are limited. In addition, introducing the large steric hindrance of a sulfur atom into the metal–carbene through cleavage of the S–C bond to construct a sulfur-substituted quaternary carbon is generally done *via* the rearrangement of sulfonium ylide,^14^ where the employment of a noble metal catalyst is demanded. Sulfur-containing compounds, involving diaryl sulfides and their higher oxidation homologues, display a variety of biological and pharmaceutical activities,^15^ such as antifungal, antibacterial, and antitumoral activities. To our knowledge, direct cyanothiolation of metal–carbene species to construct sulfur-containing nitriles remains unexplored. Based on our previous studies^16^ on copper-catalyzed coupling reactions for the formation of carbon–heteroatom bonds, herein, we propose a copper-catalyzed cyanothiolation of *N*-tosylhydrazones with thiocyanates to incorporate a sulfur-substituted quaternary carbon center through a copper carbene species promoting S–CN bond cleavage and a C–CN/C–S bond reconstruction process (Scheme 1b). This protocol to install both the sulfur and cyano group simultaneously in a one-step manner to access diverse α-arylthioalkanenitriles would find its potential applications in the fields of pharmaceutical and materials science.

## Results and discussion

We initiated our studies with the reaction between the *N*-tosylhydrazone of acetophenone (**1a**) and 1.0 equiv. of 1-methyl-4-thiocyanatobenzene (**2a**) with 5 mol% CuTC and 2 equiv. of DBU in 0.5 mL of MeCN. Gratifyingly, the desired product **3aa** was given in 70% yield after 12 h at 90 °C under a nitrogen atmosphere. Various types and loadings of copper catalysts were then screened (Table 1, entries 1–6), and the yield of **3aa** increased to 76% when 10 mol% CuSCN was used as a catalyst (entry 6). An investigation into the effects of the **2a** and DBU loadings (entries 6–9) revealed that when employing 1.5 equiv. of **2a** and 1 equiv. of DBU, the yield could reach 94% (entry 8). It was found that different solvents did not have much effect on the yield (entries 10–11). Additionally, control experiments showed that no reaction occurred in the absence of the Cu catalyst or base.

**Table 1:** Optimization of the reaction conditions^*a*^

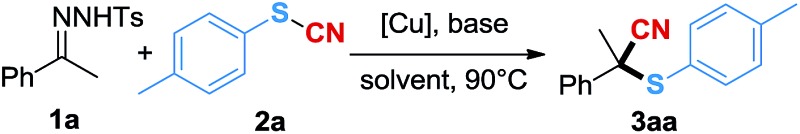					
Entry^*a*^	[Cu] (mol%)	**2a** (equiv.)	Base (equiv.)	Solvent	Yield^*b*^ (%)
1	CuTC (5)	1.0	DBU (2)	MeCN	70
2	CuSCN (5)	1.0	DBU (2)	MeCN	58
3	Cu(MeCN)_4_PF_6_ (5)	1.0	DBU (2)	MeCN	21
4	CuI (5)	1.0	DBU (2)	MeCN	36
5	CuTC (10)	1.0	DBU (2)	MeCN	55
6	CuSCN (10)	1.0	DBU (2)	MeCN	76
7	CuSCN (10)	1.0	DBU (1)	MeCN	83
**8**	**CuSCN (10)**	**1.5**	**DBU (1)**	**MeCN**	**94 (91)** ^*c*^
9	CuSCN (10)	2.0	DBU (1)	MeCN	81
10	CuSCN (10)	1.5	DBU (1)	DCE	n.d.
11	CuSCN (10)	1.5	DBU (1)	DMF	52

^*a*^Reaction conditions: all reactions were performed with **1a** (0.1 mmol), **2a**, catalyst and DBU in 0.5 mL of solvent at 90 °C under a nitrogen atmosphere for 12 h.

^*b*^GC-MS yield using *n*-dodecane as an internal standard. n.d. = not detected.

^*c*^Isolated yield.

With the optimal reaction conditions in hand, we subsequently examined a series of *N*-tosylhydrazones for this transformation (Table 2). Both the electron-rich groups (Me, MeO and Ph) and the electron-poor groups (F, CF_3_, Br, CO_2_Me, CN and SO_2_Me) at the *para*-position of the phenyl ring were tolerated in this conversion, and the corresponding products (**3ba–3ja**) were given in good to excellent yields. When the *meta*-substituted group was methyl (**3ka**) or chloride (**3la**), the reaction gave the desired products in 90% and 87% yields, respectively. The difluoro-substituted substrates could transfer to the corresponding product **3ma** in 78% isolated yield. It should be noted that naphthalene and the heterocyclic (furan and thiophene) substrates underwent the transformations smoothly, affording the desired products **3na–3pa** in 95%, 77% and 81% yields, respectively. Moreover, replacing the R^1^ group with a sterically hindered isopropyl (**1q**) or cyclopropyl (**1r**) had no obvious impact on the yields, and the corresponding products could be generated in good yields (**3qa–3ra**). It should be noted that when using the *N*-tosylhydrazones bearing tetrahydronaphthalene (**1s**) and chroman (**1t**) as the substrates, the cyanothiolation reaction gave the corresponding products **3sa** and **3ta** in 87% and 92% yields. The structure of **3na** was further confirmed by X-ray crystallographic analysis.

**Table 2:** Substrate scope of *N*-tosylhydrazones^*a*^

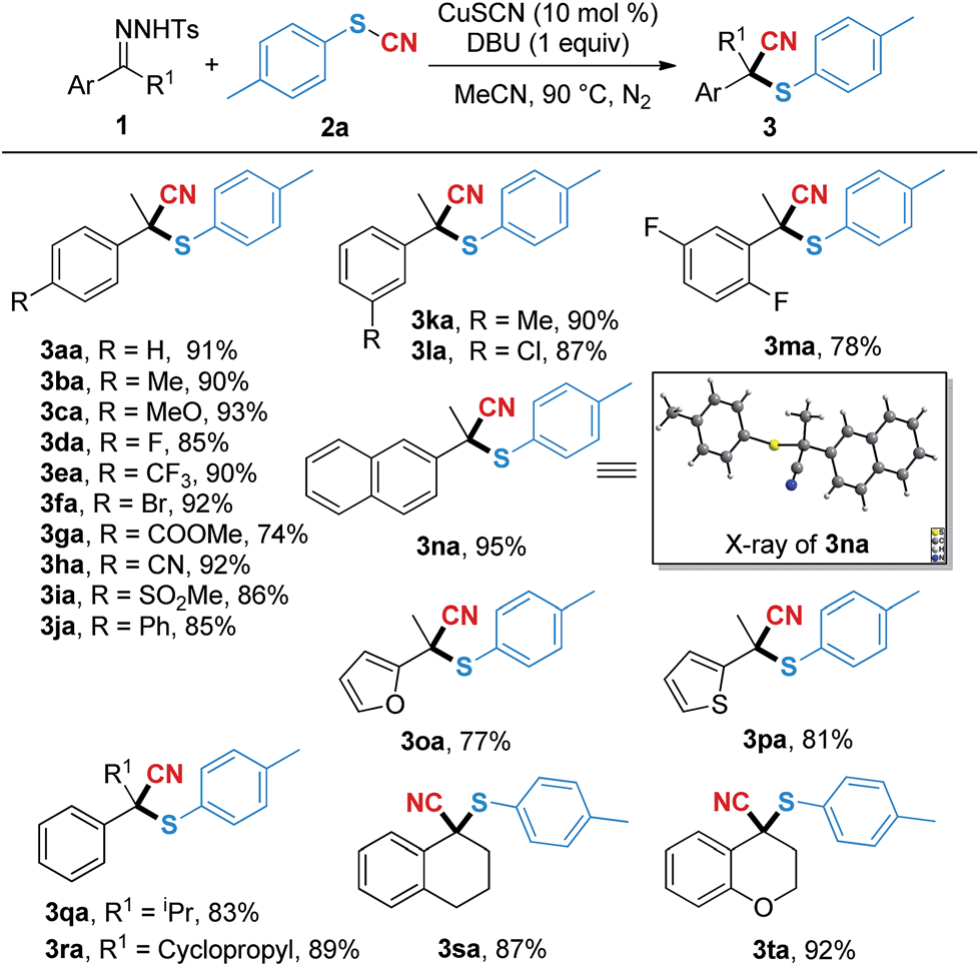

^*a*^Reaction conditions: **1** (0.2 mmol), **2a** (0.3 mmol), CuSCN (0.02 mmol) and DBU (0.2 mmol) in 1.0 mL of MeCN at 90 °C for 12 h. Isolated yield.

Next, the substrate scope of the thiocyanates was examined and the results are shown in Table 3. Electron-withdrawing and electron-donating groups were well tolerated on the aryl rings of the thiocyanates (**3ac–3ag**). Among them, substrates with halogen groups, **3ac** and **3ag**, delivered the expected products in relatively lower yields of 88% and 82%, respectively. When 2-thiocyanatonaphthalene was used as a substrate, the corresponding product **3ah** was isolated in 95% yield. However, the yield of **3ai**, bearing a pyridine ring, was decreased to 53%, while the thiocyanates containing other heterocycles such as furan, thiophene and 1-methylindole were suitable substrates and converted to the desired products in good yields (**3aj–3al**). Moreover, when thiocyanatocyclohexane was used in the reaction, a 42% isolated yield of product **3am** could be obtained.

**Table 3:** Substrate scope of thiocyanates^*a*^

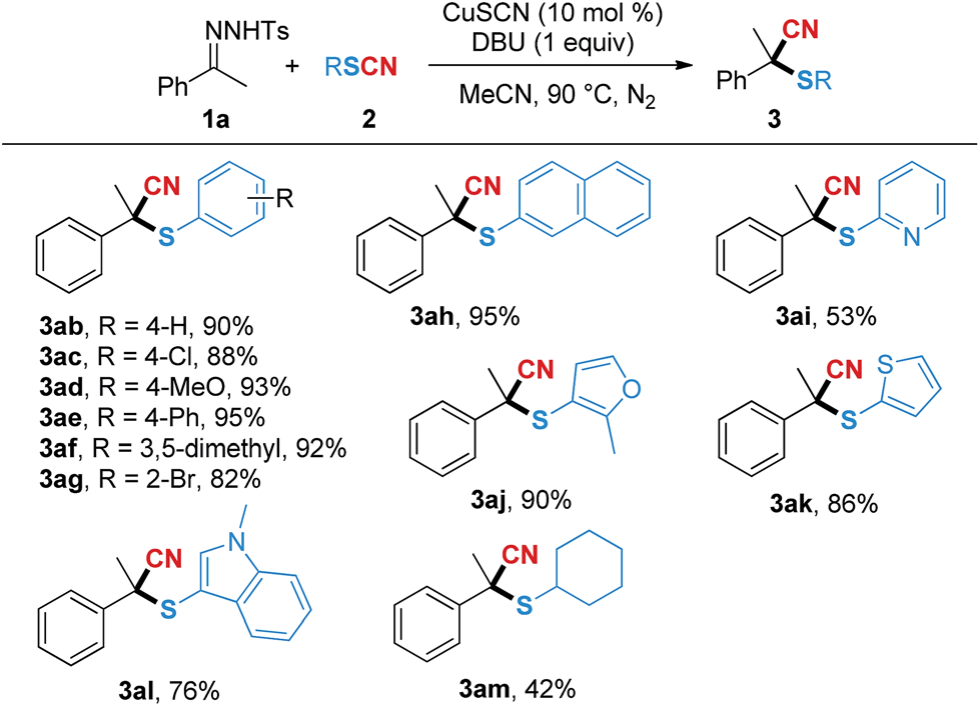

^*a*^Reaction conditions: **1a** (0.2 mmol), **2** (0.3 mmol), CuSCN (0.02 mmol), and DBU (0.2 mmol) in a 1.0 mL MeCN at 90 °C for 12 h. Isolated yield.

Interestingly, when the *ortho*-substituted (*o*-methyl and *o*-chloro) *N*-tosylhydrazones were used as the substrates, the reaction gave the nitrile products (**3ua** and **3va**) in 91% and 95% isolated yields instead of the cyanothiolation products (Table 4, eqn (1)). We suspected that the reaction might first undergo the cyano group insertion process after the cleavage of the S–CN bond, and subsequently, the release of the sulfur group was promoted due to the space hindrance of the *ortho*-substituents. Moreover, when the *ortho*-fluoro-substituted *N*-tosylhydrazone was employed, the *ortho*-thiophenyl-substituted nitrile product **3wa** was afforded in 39% yield (Table 4, eqn (2)), suggesting that the cleaved sulfur group was further involved in the coupling reaction^17^ under copper catalysis. In order to validate our hypothesis, we used the *N*-tosylhydrazone of benzaldehyde as a substrate to react with thiocyanatobenzene **2a** under the standard reaction conditions. As expected, the unstable intermediate produced by the insertion of the cyano group underwent the subsequent coupling process to give fumaronitrile **3xa** in 68% yield (Table 4, eqn (3)), which could not be obtained directly through the dimerization of phenylacetonitrile in the current system (see the ESI† for details).

**Table 4:** Formation of nitrile products^*a*^ ^,^ ^*b*^

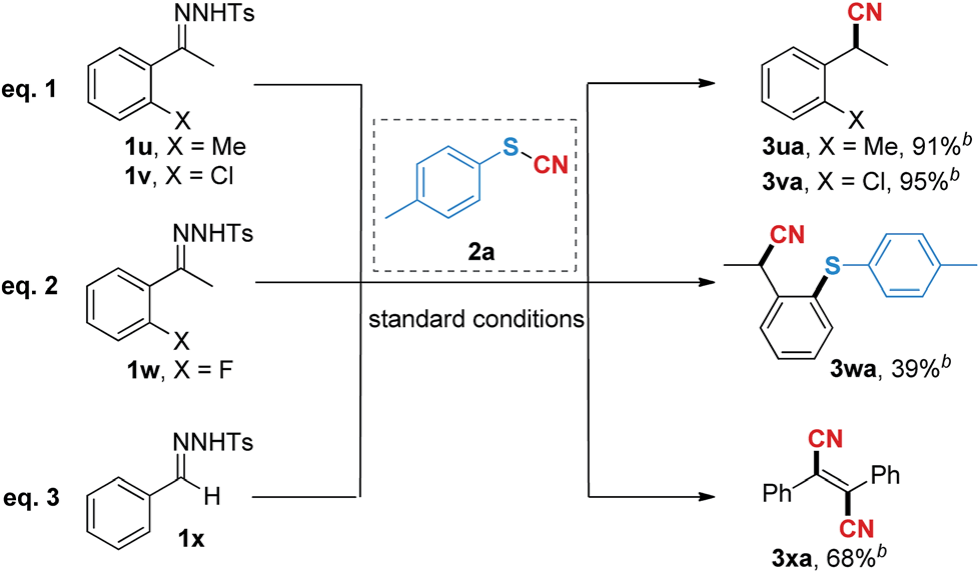

^*a*^Reaction conditions: **1** (0.2 mmol), **2a** (0.3 mmol), CuSCN (0.02 mmol), and DBU (0.2 mmol) in 1.0 mL of MeCN at 90 °C for 12 h.

^*b*^Isolated yield.

**Scheme 2:**
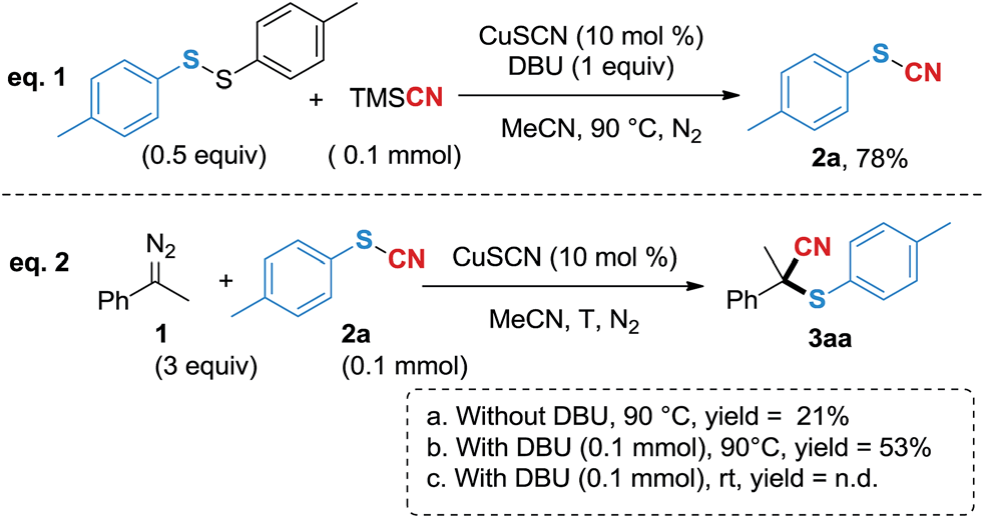
Mechanistic studies. GC-MS yield using *n*-dodecane as an internal standard. n.d. = not detected.

In order to further study the reaction mechanism, several control experiments were performed (Scheme 2). Firstly, when using 1,2-di-*p*-tolyldisulfane and TMSCN as the substrates, **2a** was generated in 78% yield (Scheme 2, eqn (1)), which indicated that thiocyanate could be generated in a good yield under the standard conditions with TMSCN as the cyano source. To validate that the copper carbene species would promote the S–CN bond cleavage, we next mixed (1-diazoethyl) benzene^18^ and **2a** in the reaction system without a base. However, **3aa** was generated in only 21% yield (Scheme 2, eqn (2a)), which might be due to the instability of the diazo compound at higher temperatures. In contrast, when DBU was added, the yield of **3aa** was increased to 53% (Scheme 2, eqn (2b)), suggesting that DBU probably acted as both a ligand and a base to facilitate the reaction. However, when the reaction was conducted at room temperature with DBU added, **2a** did not participate in the conversion (Scheme 2, eqn (2c)), therefore, a higher energy was required for the cleavage of the S–CN bond in this cyanothiolation reaction.

**Scheme 3:**
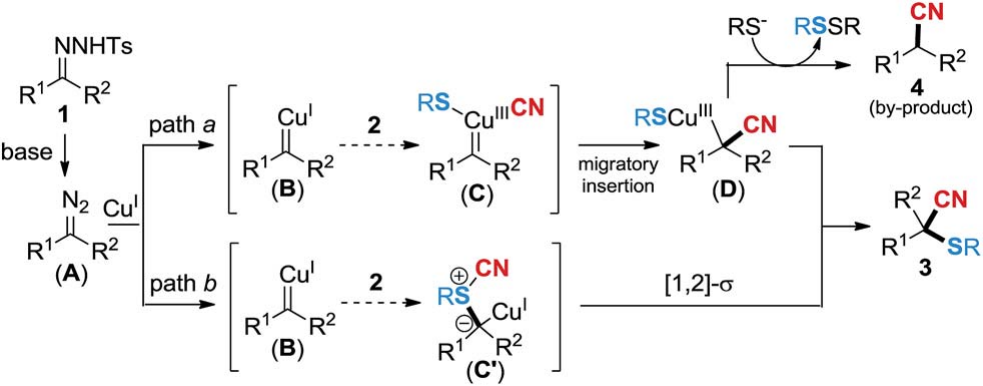
Proposed mechanism.

Based on the above experimental results, a tentative mechanism is proposed for the copper-catalyzed cyanothiolation of *N*-tosylhydrazone in Scheme 3. Initially, the diazo substrate **A** is released slowly from the *N*-tosylhydrazone in the presence of DBU, which would react with Cu^I^ to give the copper carbene species **B**. We speculate that the generation of the product might be *via* two paths. In path a, the interaction of thiocyanate **2** and the copper carbene species **B** leads to the formation of the copper^III^ carbene species **C**,^12^ followed by migratory insertion^18a,19^ to give the intermediate **D**. The reductive elimination of **D** provides the major product **3** and regenerates Cu^I^. In addition, a small amount of intermediate **D** would take off the RS^–^ group in the presence of the thiophenol anion,^20^ and disulfide ether would be generated as a by-product, followed by protonation^19^ to release the nitrile by-product **4**. In addition, the process of sulfur ylide rearrangement should be considered in the transformation as well. The copper carbene species **B** could react with thiocyanate **2** to generate sulfur ylide **C′** ^14^ *via* path b, which may subsequently undergo [1,2]-sigmatropic rearrangement^14*d*^ to access product **3**.

## Conclusions

In summary, we have demonstrated a copper-catalyzed cyanothiolation reaction of *N*-tosylhydrazones with thiocyanates to give α-arylthioalkanenitriles in high yields. Sulfur-containing nitriles have important research value in the life sciences due to their diverse biological activities resulting from the sulfur^21^ and cyano functional groups, which may dramatically modify the properties of each other, and even serve as intermediates in diverse transformations. This cyanothiolation reaction provides a valuable and efficient strategy to introduce both sulfur and cyano groups onto a single carbon center *via* a copper carbene species-promoting S–CN bond cleavage and C–CN/C–S bond reconstruction process. Furthermore, the inexpensive copper catalyst, readily available substrates and simple operation provide great advantages for the transformation. We speculated that the cyanothiolation reaction might occur through formation and conversion of the copper^III^ carbene species or [1,2]-sigmatropic rearrangement of a sulfur ylide. Further investigations into chiral synthetic applications and elucidating the mechanism of this novel reaction are ongoing in our laboratory.

## Conflicts of interest

There are no conflicts to declare.
