# Supplementary material for: Copper-catalyzed cyanothiolation to incorporate a sulfur-substituted quaternary carbon center
Source: Chem Sci. 2017 Aug 15;8(10):7047–51. doi: 10.1039/c7sc02867a (PMC5637458; doi:10.1039/c7sc02867a)

Electronic Supplementary Material (ESI) for Chemical Science.
This journal is © The Royal Society of Chemistry 2017

Supporting Information

Copper-Catalyzed Cyanothiolation to Incorporate Sulfur-Substituted Quaternary Carbon Center

Yubing Huang, Xianwei Li, Xu Wang, Yue Yu, Jia Zheng, Wanqing Wu, and Huanfeng Jiang**

Key Laboratory of Functional Molecular Engineering of Guangdong Province, School of Chemistry and Chemical Engineering, South China University of Technology, Guangzhou 510640, P. R. China

E-mail: cewuwq@scut.edu.cn, jianghf@scut.edu.cn

Contents

1. General Information……………………………..…….…………………………S2
2. General Experimental Procedure…………..……..…….…..…………………....…S2
3. Mechanistic Studies.........................…..………………………………………......S3
4. Characterization Data for All Products…...………......…………………….……...S4
5. Reference…......……………………………………………………….….......S12
6. X-ray Diffraction Parameters and Data for **3na**………..…………………..…......S13
7. NMR Spectra for All Compounds……..……………………………...…..…...S14

1. General Information

Melting points were determined with a Buchi Melting Point B-545 instrument. ^{1}H and ^{13}C NMR spectra were recorded using a Bruker DRX-400 spectrometer using $CDCl_3$ as solvent. The chemical shifts are referenced to signals at 7.26 and 77.0 ppm, respectively, and chloroform is solvent with TMS as the internal standard. IR spectra were obtained either as potassium bromide pellets or as liquid films between two potassium bromide pellets with a Bruker TENSOR 27 spectrometer. Mass spectra were recorded on a Thermo Scientific ISQ gas chromatograph-mass spectrometer. The data of HRMS was carried out on a high-resolution mass spectrometer (LCMS-IT-TOF). TLC was performed by using commercially prepared 100-400 mesh silica gel plates and visualization was effected at 254 nm. Unless otherwise noted, all reagents and solvents were obtained from commercial suppliers and used without further purification.

2. General Experimental Procedure

A. Typical Procedure for the Synthesis of *N*-Tosylhydrazones

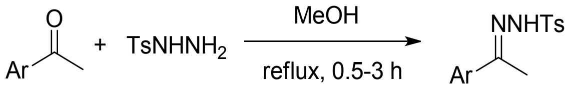

1

A mixture of ketone compound (5.0 mmol) and *p*-toluenesulfonhydrazide (5.0 mmol) in 7.5 mL MeOH was stirred at 60 °C for 0.5-3 h to afford the corresponding *N*-tosylhydrazone **1** as white precipitate. After that, the precipitate was washed and filtered with petroleum ether twice and dried under vacuum to provide the pure compounds.

B. General Procedure for the Synthesis of Thiocyanates 1a-1f, 1i-1k and 1m

A mixture of thioether compound (1.0 mmol), I_2 (2.0 mmol) and KOAc (5.0 mmol) in 5 mL CH_3NO_2 was added to a test tube. The mixture was stirred under air at 90 °C for 12 h. After that, water was added and extracted with ethyl acetate twice. The combined organic phase was dried over Na_2SO_4 and concentrated. The residue was purified by flash column chromatography on silica gel with petroleum ether/ethyl acetate (PE/EA = 100: 1–10: 1) as the eluent to afford the corresponding products.

C. General Procedure for the Synthesis of Thiocyanates 1g

A mixture of 2-bromobenzenethiol (1.0 mmol), CuCN (2.0 mmol) and TMEDA (2.0 mmol) in 3 mL MeCN was added to a test tube. The mixture was stirred in an open vessel for 18 h at room temperature. After that, water was added and extracted with ethyl acetate twice. The combined organic phase was dried over Na_2SO_4 and concentrated. The residue was purified by flash column chromatography on silica gel with petroleum ether as the eluent to afford the corresponding product **1g**.

D. General Procedure for the Synthesis of Thiocyanates 1l

To a mixture of 1-methyl-1*H*-indole (1 mmol) and NH_4SCN (2 mmol) in CH_3OH (1 mL) in a 10 mL test tube was added DDQ (1.25 mmol). The resulting mixture irradiated for 5 min by cooling of samples at 25 °C. The progress of the reaction was monitored by TLC (eluent: *n*-hexane-ethyl acetate, 9: 1). The solvent was evaporated. The residue was diluted with water (10 mL) and extracted with chloroform (3×10 mL). The combined organic layers were dried over $MgSO_4$ and evaporated. The resulting crude product was purified by chromatography on silica gel (eluent: *n*-hexane-ethyl acetate, 9: 1) to afford the pure product **1l**.

E. General Procedure for the Synthesis of (1-Diazoethyl)benzene

The (1-diazoethyl)benzene was prepared following a literature procedure.[1] To a solution of acetophenone (20.0 mmol, 1.0 equiv) in methanol (20 mL) was added hydrazine hydrate (2.9 mL, 60.0 mmol, 3.0 equiv) and the mixture was stirred at 80 °C for 3 h in a sealed vial. The solvent was removed under reduced pressure and the crude residue diluted with water (25 mL) and CH_2Cl_2 (25 mL). The mixture was separated and the aqueous layer extracted with CH_2Cl_2 (3×25 mL) and the combined organic extracts were dried over $MgSO_4$, filtered and evaporated under reduced pressure to provide the desired hydrazone. The crude hydrazone was used for the generation of corresponding diazo compound without further purification. Activated manganese dioxide (690 mg, 8.0 mmol) was added to a cold solution (0 °C) of hydrazone (2.0 mmol) and $MgSO_4$ (361 mg, 3.0 mmol) in CH_2Cl_2 (5 mL), at which point the mixture turned deep red immediately. The reaction mixture was stirred for 2 h at 0 °C and 1 h at room temperature, and then filtered off through cotton to provide the aryldiazoalkanes as a clear, deep red solution. The solution of compound was used without further purification.

F. General Procedure for the Synthesis of desired Products

A mixture of *N*-tosylhydrazone (0.2 mmol), thiocyanates (0.3 mmol), CuSCN (10 mol %), DBU (0.2 mmol) and 1.0 mL MeCN was added to a sealed tube. The mixture was stirred at 90 °C in N_2 atmosphere for 12 h. After that, water was added and extracted with ethyl acetate twice. The combined organic phase was dried over Na_2SO_4 and concentrated. The residue was purified by flash column chromatography on silica gel with petroleum ether/ethyl acetate (PE/EA = 20: 1–5: 1) as the eluent to afford the corresponding products.

3. Mechanistic Studies

A. Supplementary Experiment

N-Tosylhydrazone of benzaldehyde **1x**, thiocyanatobenzene **2a**, CuSCN (10 mol %), DBU (0.2 mmol) and 1.0 mL MeCN were mixed in a sealed tube. After stirring at 90 °C in N_2 atmosphere for 12 h, fumaronitrile **3xa** was isolated in 68% yield and a trace amount of phenylacetonitrile **4xa** was detected (Scheme S1, a). In order to verify whether product **3xa** was obtained through the dimerization of phenylacetonitrile in the current system, phenylacetonitrile **4xa** (0.1 mmol), CuSCN (10 mol %), DBU (0.2 mmol) and 0.5 mL MeCN were then mixed in a sealed tube. After stirring at 90 °C in N_2 atmosphere for 12 h, no dimerization product fumaronitrile **2** was detected (Scheme S1, b), which suggested that the formation of **3xa** was not through the dimerization of phenylacetonitrile.

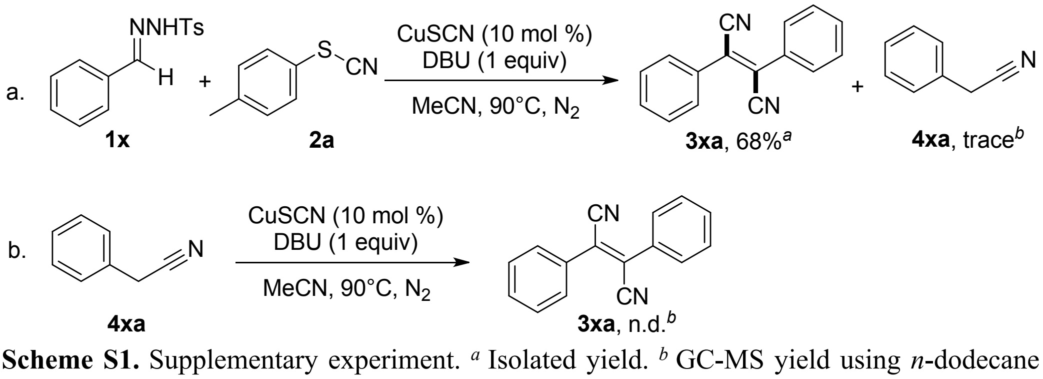

Scheme S1. Supplementary experiment. [a] Isolated yield. [b] GC-MS yield using *n*-dodecane as an internal standard. n.d. = not detected.

B. Proposed Mechanism for the Formation of 3xa

A tentative mechanism for the formation of **3xa** was proposed in Scheme S2. Initially, the diazo substrate **a** is released slowly from the *N*-tosylhydrazone **1x** in the presence of DBU, which would react with CuI to give the copper carbene species **b**. The interaction between copper carbene

species **b** and thiocyanate **2a** may be via path *a* or path *b* (see the text section, Scheme 3) to generate the cyanothiolation product **c**, which can undergo deprotonation by the base to give rise to anion **d**. Subsequently, the coupling of anion **d** with cyanothiolation product **c** generates the intermediates **e** with simultaneous release of thiophenol anion. After deprotonation and the release of thiophenol anion, fumaronitrile **3xa** is finally obtained.

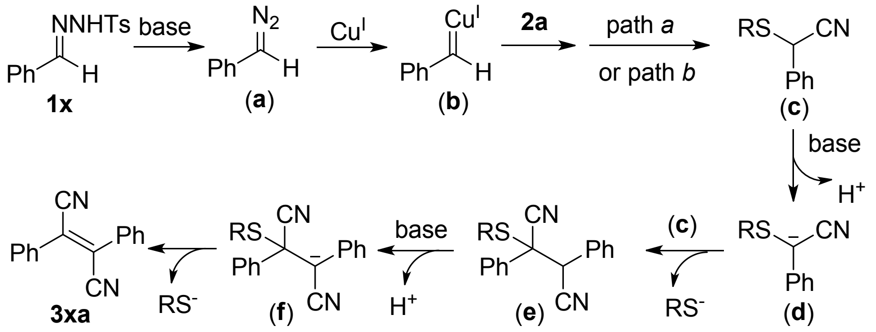

Scheme S2. Proposed mechanism for the formation of **3xa**.

4. Characterization Data for All Products

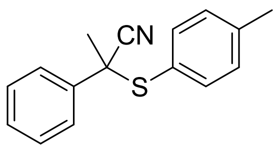

2-Phenyl-2-(*p*-tolylthio)propanenitrile (3aa): Yield: 46 mg (91%), yellow oil. ^{1}H NMR (400 MHz, $CDCl_3$, ppm): δ = 7.52 (dd, *J* = 7.8, 1.4 Hz, 2H), 7.36 (m, 5H), 7.13 (d, *J* = 8.0 Hz, 2H), 2.36 (s, 3H), 2.01 (s, 3H); ^{13}C NMR (101 MHz, $CDCl_3$, ppm): δ = 140.7, 137.8, 136.7, 129.8, 128.7, 128.6, 126.8, 126.2, 120.9, 48.9, 27.4, 21.4; IR (KBr, cm^{-1}): *v* = 2927, 2860, 2232, 1602, 1451, 1075, 809, 695, 501; HRMS (ESI) calc. $C_{16}H_{15}NNaS$ $[M+Na]^+$: 276.0817, found: 276.0813.

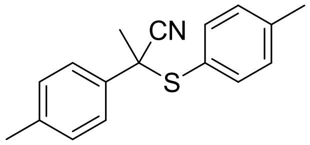

2-(*p*-Tolyl)-2-(*p*-tolylthio)propanenitrile (3ba): Yield: 48 mg (90%), yellow oil. ^{1}H NMR (400 MHz, $CDCl_3$, ppm): δ = 7.41 (d, *J* = 8.3 Hz, 2H), 7.37 (d, *J* = 8.1 Hz, 2H), 7.18 (d, *J* = 8.0 Hz, 2H), 7.14 (d, *J* = 7.9 Hz, 2H), 2.38 (d, *J* = 3.4 Hz, 6H), 1.98 (s, 3H); ^{13}C NMR (101 MHz, $CDCl_3$, ppm): δ = 140.7, 138.5, 136.7, 134.8, 129.8, 129.4, 127.0, 126.1, 121.0, 48.6, 27.5, 21.4, 21.1. IR (KBr, cm^{-1}): *v* = 2927, 2232, 1597, 1503, 1448, 1031, 813, 741, 511; HRMS (ESI) calc. $C_{17}H_{17}NNaS$ $[M+Na]^+$: 290.0974, found: 290.0970.

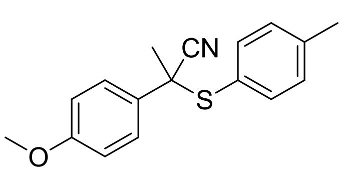

2-(4-Methoxyphenyl)-2-(*p*-tolylthio)propanenitrile (3ca): Yield: 53 mg (93%), yellow solid. M.p.: 69-71 ºC. ^{1}H NMR (400 MHz, $CDCl_3$, ppm): δ = 7.43 (m, 2H), 7.35 (d, *J* = 8.1 Hz, 2H), 7.13 (d, *J* = 8.0 Hz, 2H), 6.88 (m, 2H), 3.84 (s, 3H), 2.37 (s, 3H), 1.98 (s, 3H); ^{13}C NMR (101 MHz, $CDCl_3$, ppm): δ = 159.7, 140.6, 136.7, 129.8, 129.7, 127.6, 127.0, 121.1, 114.0, 55.4, 48.3, 27.5, 21.3. IR (KBr, cm^{-1}): *v* = 2923, 2855, 1609, 1502, 1250, 1027, 814, 491; HRMS (ESI) calc. $C_{17}H_{17}NNaOS$ $[M+Na]^+$: 306.0923, found: 306.0917.

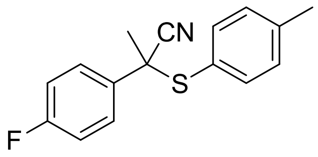

2-(4-Fluorophenyl)-2-(*p*-tolylthio)propanenitrile (3da): Yield: 46 mg (85%), yellow oil. ^{1}H NMR (400 MHz, $CDCl_3$, ppm): δ = 7.44 (m, 2H), 7.29 (d, *J* = 8.1 Hz, 2H), 7.11 (d, *J* = 7.9 Hz, 2H), 7.02 (m, 2H), 2.34 (s, 3H), 1.98 (s, 3H); ^{13}C NMR (101 MHz, $CDCl_3$, ppm): δ = 162.5 (d, *J* = 248.7 Hz), 140.9, 136.7, 133.8 (d, *J* = 3.2 Hz), 129.9, 128.2 (d, *J* = 8.4 Hz), 126.6, 120.8, 115.6 (d, *J* = 21.9 Hz), 48.3, 27.5, 21.3; IR (KBr, cm^{-1}): *ν* = 2928, 2233, 1600, 1505, 1235, 1165, 823, 746, 520; HRMS (ESI) calc. $C_{16}H_{14}FNNaS$ $[M+Na]^+$: 294.0723, found: 294.0720.

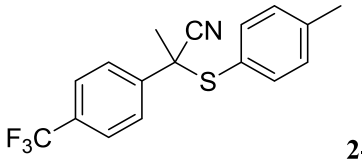

2-(*p*-Tolylthio)-2-(4-(trifluoromethyl)phenyl)propanenitrile (3ea): Yield: 58 mg (90%), yellow oil. ^{1}H NMR (400 MHz, $CDCl_3$, ppm): δ = 7.62 (s, 4H), 7.30 (d, *J* = 8.0 Hz, 2H), 7.13 (d, *J* = 8.0 Hz, 2H), 2.36 (s, 3H), 2.04 (s, 3H); ^{13}C NMR (101 MHz, $CDCl_3$, ppm): δ = 142.0, 141.2, 136.7, 130.8 (q, *J* = 32.8 Hz), 130.0, 126.8, 126.1, 125.7 (q, *J* = 3.7 Hz), 123.8 (q, *J* = 272.2 Hz), 120.3, 48.6, 27.3, 21.3. IR (KBr, cm^{-1}): *ν* = 2929, 2861, 2234, 1613, 1410, 1326, 1128, 1076, 835, 513; HRMS (ESI) calc. $C_{17}H_{14}F_3NNaS$ $[M+Na]^+$: 344.0691, found: 344.0688.

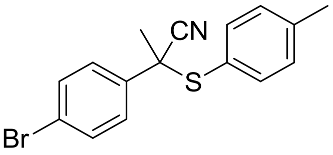

2-(4-Bromophenyl)-2-(*p*-tolylthio)propanenitrile (3fa): Yield: 61 mg (92%), yellow oil. ^{1}H NMR (400 MHz, $CDCl_3$, ppm): δ = 7.48 (m, 2H), 7.37 (m, 2H), 7.32 (d, *J* = 8.1 Hz, 2H), 7.13 (d, *J* = 8.0 Hz, 2H), 2.36 (s, 3H), 1.99 (s, 3H).; ^{13}C NMR (101 MHz, $CDCl_3$, ppm): δ =141.0, 137.1, 136.7, 131.8, 130.0, 128.0, 126.4, 122.7, 120.5, 48.5, 27.3, 21.4. IR (KBr, cm^{-1}): *ν* = 2925, 2858, 2236, 1648, 1499, 1393, 1080, 1010, 819, 512; HRMS (ESI) calc. $C_{16}H_{14}BrNNaS$ $[M+Na]^+$: 353.9923, found: 353.9919.

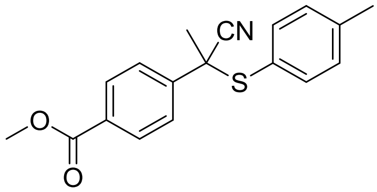

Methyl 4-(1-cyano-1-(*p*-tolylthio)ethyl)benzoate (3ga): Yield: 46 mg (74%), white solid. M.p.: 72-73 ℃. ^{1}H NMR (400 MHz, $CDCl_3$, ppm): δ = 7.99 (m, 2H), 7.53 (m, 2H), 7.26 (d, *J* = 8.1 Hz, 2H), 7.07 (d, *J* = 8.3 Hz, 2H), 3.92 (s, 3H), 2.31 (s, 3H), 2.00 (s, 3H); ^{13}C NMR (101 MHz, $CDCl_3$, ppm): δ = 166.2, 142.8, 141.0, 136.6, 130.3, 129.9, 129.9, 126.3, 126.2, 120.4, 52.2, 48.8, 27.2, 21.3; IR (KBr, cm^{-1}): *ν* = 2945, 2233, 1725, 1606, 1442, 1283, 1191, 1112, 814, 703, 515; HRMS (ESI) calc. $C_{18}H_{17}NNaO_2S$ $[M+Na]^+$: 334.0872, found: 334.0873.

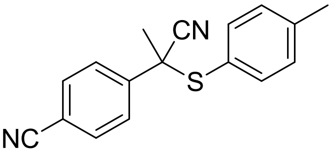

4-(1-Cyano-1-(*p*-tolylthio)ethyl)benzonitrile (3ha): Yield: 51 mg (92%), yellow oil. ^{1}H NMR (400 MHz, $CDCl_3$, ppm): δ = 7.64 (m, 2H), 7.56 (m, 2H), 7.25 (d, *J* = 8.1 Hz, 2H), 7.11 (d, *J* = 7.9 Hz, 2H), 2.35 (s, 3H), 2.03 (s, 3H); ^{13}C NMR (101 MHz, $CDCl_3$, ppm): δ = 143.2, 141.3, 136.6, 132.4, 130.0, 127.1, 125.8, 119.9, 118.1, 112.5, 48.7, 27.0, 21.4; IR

(KBr, cm^{-1}): v = 2926, 2859, 2231, 1917, 1601, 1496, 1402, 1272, 1206, 1021, 822, 749, 550; HRMS (ESI) calc. $C_{17}H_{14}N_2NaS$ $[M+Na]^+$: 301.0770, found: 301.0756.

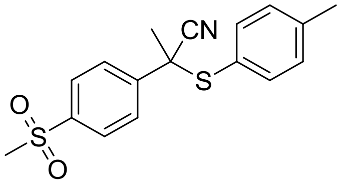

2-(4-(Methylsulfonyl)phenyl)-2-(*p*-tolylthio)propanenitrile (3ia): Yield: 57 mg (86%), yellow oil. ^1H NMR (400 MHz, $CDCl_3$, ppm): δ = 7.91 (d, J = 8.6 Hz, 2H), 7.66 (d, J = 8.6 Hz, 2H), 7.26 (d, J = 8.1 Hz, 2H), 7.10 (d, J = 7.9 Hz, 2H), 3.06 (s, 3H), 2.33 (s, 3H), 2.02 (s, 3H); ^{13}C NMR (101 MHz, $CDCl_3$, ppm): δ = 144.1, 141.4, 140.7, 136.7, 130.1, 127.8, 127.4, 125.78, 120.0, 48.6, 44.5, 27.2, 21.3; IR (KBr, cm^{-1}): v = 2926, 1594, 1399, 1310, 1152, 1090, 957, 758, 548; HRMS (ESI) calc. $C_{17}H_{17}NNaO_2S_2$ $[M+Na]^+$: 354.0593, found:354.0592.

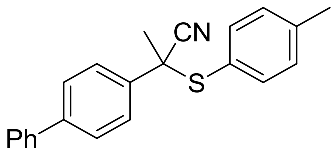

2-([1,1'-Biphenyl]-4-yl)-2-(*p*-tolylthio)propanenitrile (3ja): Yield: 56 mg (85%), white solid. M.p.: 138-139 ºC. ^1H NMR (400 MHz, $CDCl_3$, ppm): δ = 7.57 (m, 6H), 7.44 (t, J = 7.5 Hz, 2H), 7.35 (dd, J = 10.0, 7.7 Hz, 3H), 7.10 (d, J = 7.9 Hz, 2H), 2.33 (s, 3H), 2.00 (s, 3H); ^{13}C NMR (101 MHz, $CDCl_3$, ppm): δ = 141.5, 140.8, 140.1, 136.8, 136.8, 129.9, 128.9, 127.8, 127.3, 127.1, 126.8, 126.7, 120.9, 48.7, 27.4, 21.4; IR (KBr, cm^{-1}): v = 3032, 2928, 2855, 2230, 1649, 1596, 1482, 1393, 1244, 1069, 816, 759, 697, 507; HRMS (ESI) calc. $C_{22}H_{19}NNaS$ $[M+Na]^+$: 352.1130, found: 352.1134.

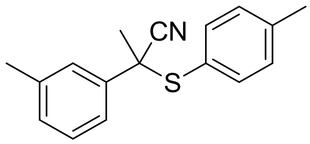

2-(*m*-Tolyl)-2-(*p*-tolylthio)propanenitrile (3ka): Yield: 48 mg (90%), yellow oil. ^1H NMR (400 MHz, $CDCl_3$, ppm): δ = 7.36 (dd, J = 14.4, 8.4 Hz, 4H), 7.29 (d, J = 7.7 Hz, 1H), 7.17 (t, J = 6.1 Hz, 3H), 2.39 (s, 6H), 2.01 (s, 3H); ^{13}C NMR (101 MHz, $CDCl_3$, ppm): δ = 140.7, 138.5, 137.6, 136.8, 129.8, 129.4, 128.6, 126.9, 126.9, 123.2, 121.0, 48.8, 27.4, 21.4, 21.3; IR (KBr, cm^{-1}): v = 3032, 2926, 2863, 2232, 1599, 1486, 1449, 1072, 804, 699, 487; HRMS (ESI) calc. $C_{17}H_{17}NNaS$ $[M+Na]^+$: 290.0974, found: 290.0972.

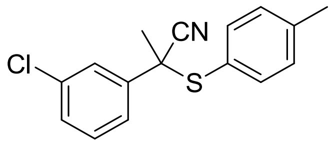

2-(3-Chlorophenyl)-2-(*p*-tolylthio)propanenitrile (3la): Yield: 50 mg (87%), yellow oil. ^1H NMR (400 MHz, $CDCl_3$, ppm): δ = 7.45 (t, J = 1.7 Hz, 1H), 7.34 (m, 1H), 7.29 (m, 4H), 7.12 (d, J = 7.9 Hz, 2H), 2.35 (s, 3H), 1.98 (s, 3H); ^{13}C NMR (101 MHz, $CDCl_3$, ppm): δ =141.1, 139.9, 136.7, 134.7, 129.9, 129.9, 128.8, 126.5, 126.3, 124.5, 120.4, 48.5, 27.3, 21.3; IR (KBr, cm^{-1}): v = 2927, 2860, 2233, 1723, 1585, 1475, 1275, 1218, 1084, 803, 699, 487; HRMS (ESI) calc. $C_{16}H_{14}ClNNaS$ $[M+Na]^+$: 310.0428, found: 310.0424.

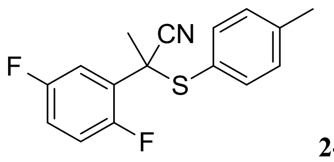

2-(2,5-Difluorophenyl)-2-(*p*-tolylthio)propanenitrile (3ma): Yield: 45 mg (78%), yellow oil. ^1H NMR (400 MHz, $CDCl_3$, ppm): δ = 7.30 (m, 2H), 7.10 (m, 2H), 7.02 (m, 3H), 2.33 (s, 3H), 2.09 (d, J = 1.2 Hz, 3H); ^{13}C NMR (101 MHz, $CDCl_3$, ppm): δ = 157.9 (dd, J = 244.2, 2.4 Hz), 156.1 (dd, J = 248.5, 2.8 Hz), 141.2, 136.8, 129.9, 126.5 (dd, J = 11.6, 7.2 Hz),

126.1, 119.8, 118.2 (dd, J = 25.2, 8.5 Hz), 116.9 (dd, J = 23.7, 9.0 Hz), 115.8 (dd, J = 26.5, 3.2 Hz), 46.8 (dd, J = 3.4, 0.9 Hz), 25.5 (d, J = 5.6 Hz), 21.3; IR (KBr, cm^{-1}): ν = 3076, 2930, 2235, 1494, 1266, 1200, 1056, 872, 816, 750, 480; HRMS (ESI) calc. $C_{16}H_{13}F_2NNaS$ $[M+Na]^+$: 312.0629, found: 312.0627.

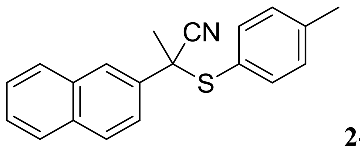

2-(Naphthalen-2-yl)-2-(*p*-tolylthio)propanenitrile (3na): Yield: 58 mg (95%), white crystal. M.p.: 91-92 ºC. ^{1}H NMR (400 MHz, $CDCl_3$, ppm): δ = 7.85 (d, J = 8.7 Hz, 1H), 7.81 (dd, J = 5.6, 3.1 Hz, 2H), 7.74 (m, 1H), 7.66 (dd, J = 8.7, 2.0 Hz, 1H), 7.48 (m, 2H), 7.27 (m, 2H), 7.01 (d, J = 7.9 Hz, 2H), 2.26 (s, 3H), 2.05 (s, 3H); ^{13}C NMR (101 MHz, $CDCl_3$, ppm): δ = 140.8, 136.7, 135.0, 133.0, 132.7, 129.8, 128.9, 128.4, 127.6, 126.9, 126.7, 125.8, 123.3, 120.9, 49.3, 27.5, 21.3; IR (KBr, cm^{-1}): ν = 3053, 2927, 2862, 2232, 1914, 1597, 1496, 1448, 1375, 1188, 1123, 1064, 815, 750, 479; HRMS (ESI) calc. $C_{20}H_{17}NNaS$ $[M+Na]^+$: 326.0974, found: 326.0977.

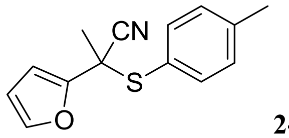

2-(Furan-2-yl)-2-(*p*-tolylthio)propanenitrile (3oa): Yield: 37 mg (77%), tan oil. ^{1}H NMR (400 MHz, $CDCl_3$, ppm): δ = 7.45 (dd, J = 1.8, 0.8 Hz, 1H), 7.29 (m, 2H), 7.15 (d, J = 7.9 Hz, 2H), 6.29 (dd, J = 3.3, 1.9 Hz, 1H), 6.16 (dd, J = 3.3, 0.8 Hz, 1H), 2.38 (s, 3H), 2.02 (s, 3H); ^{13}C NMR (101 MHz, $CDCl_3$, ppm): δ = 149.0, 143.4, 140.9, 137.0, 129.8, 126.2, 119.4, 110.6, 109.3, 43.9, 24.5, 21.4; IR (KBr, cm^{-1}): ν = 3129, 2927, 2861, 2223, 1491, 1384, 1164, 1074, 812, 746, 504; HRMS (ESI) calc. $C_{14}H_{13}NNaOS$ $[M+Na]^+$: 266.0610, found: 266.0608.

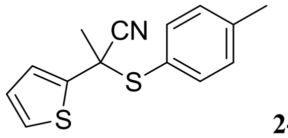

2-(Thiophen-2-yl)-2-(*p*-tolylthio)propanenitrile (3pa): Yield: 42 mg (81%), yellow oil. ^{1}H NMR (400 MHz, $CDCl_3$, ppm): δ = 7.34 (d, J = 8.1 Hz, 2H), 7.30 (dd, J = 5.1, 1.2 Hz, 1H), 7.13 (d, J = 7.9 Hz, 2H), 6.90 (dd, J = 3.6, 1.2 Hz, 1H), 6.84 (dd, J = 5.1, 3.7 Hz, 1H), 2.35 (s, 3H), 2.05 (s, 3H); ^{13}C NMR (101 MHz, $CDCl_3$, ppm): δ = 142.3, 141.0, 136.7, 129.9, 127.1, 126.7, 126.5, 126.4, 120.3, 45.6, 28.8, 21.4; IR (KBr, cm^{-1}): ν = 2922, 2855, 2233, 1595, 1439, 1230, 1048, 810, 702, 508; HRMS (ESI) calc. $C_{14}H_{13}NNaS_2$ $[M+Na]^+$: 282.0382, found: 282.0383.

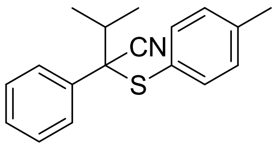

3-Methyl-2-phenyl-2-(*p*-tolylthio)butanenitrile (3qa): Yield: 47 mg (83%), yellow solid. M.p.: 81-82 ºC. ^{1}H NMR (400 MHz, $CDCl_3$, ppm): δ = 7.38 (dd, J = 7.6, 1.7 Hz, 2H), 7.28 (dd, J = 7.5, 5.6 Hz, 3H), 7.10 (d, J = 8.1 Hz, 2H), 6.97 (d, J = 8.1 Hz, 2H), 2.57 (dt, J = 13.4, 6.7 Hz, 1H), 2.28 (s, 3H), 1.48 (d, J = 6.7 Hz, 3H), 0.91 (d, J = 6.7 Hz, 3H); ^{13}C NMR (101 MHz, $CDCl_3$, ppm): δ = 140.1, 137.1, 136.5, 129.5, 128.3, 128.0, 127.2, 126.3, 118.7, 61.5, 37.7, 21.2, 19.4, 19.3; IR (KBr, cm^{-1}): ν = 3046, 2926, 2863, 2231, 1726, 1646, 1460, 1386, 1269, 1124, 1024, 810, 744, 698, 515; HRMS (ESI) calc. $C_{18}H_{19}NNaS$ $[M+Na]^+$: 304.1130, found: 304.1131.

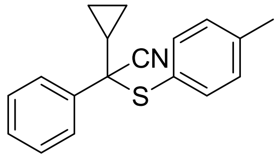

2-Cyclopropyl-2-phenyl-2-(*p*-tolylthio)acetonitrile (3ra): Yield: 50 mg (89%), yellow oil. ^{1}H NMR (400 MHz, $CDCl_3$, ppm): δ = 7.52 (m, 2H), 7.31 (m, 5H), 7.05 (d, *J* = 7.9 Hz, 2H), 2.31 (s, 3H), 1.67 (ddd, *J* = 13.3, 6.6, 4.0 Hz, 1H), 0.73 (m, 2H), 0.59 (m, 1H), 0.52 (m, 1H); ^{13}C NMR (101 MHz, $CDCl_3$, ppm): δ = 140.5, 137.6, 136.8, 129.6, 128.5, 128.5, 126.9, 126.5, 117.7, 57.3, 21.3, 19.3, 5.2, 3.2; IR (KBr, cm^{-1}): *v* = 3023, 2924, 2858, 2234, 1594, 1489, 1447, 1188, 1026, 810, 742, 694, 505; HRMS (ESI) calc. $C_{18}H_{17}NNaS$ $[M+Na]^+$: 302.0974, found: 302.0975.

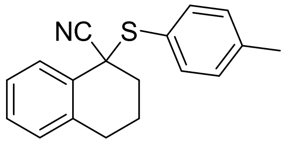

1-(*p*-Tolylthio)-1,2,3,4-tetrahydronaphthalene-1-carbonitrile (3sa): Yield: 48 mg (87%), red crystal. M.p.: 79-80 ºC. ^{1}H NMR (400 MHz, $CDCl_3$, ppm): δ = 7.55 (m, 3H), 7.22 (m, 4H), 7.12 (d, *J* = 7.4 Hz, 1H), 2.86 (m, 2H), 2.39 (s, 3H), 2.33 (m, 1H), 2.14 (m, 2H), 1.85 (ddd, *J* = 9.7, 5.4, 3.3 Hz, 1H); ^{13}C NMR (101 MHz, $CDCl_3$, ppm): δ = 140.9, 137.1, 136.1, 131.3, 130.1, 129.7, 129.4, 128.9, 127.1, 126.5, 122.0, 48.1, 32.3, 28.6, 21.4, 17.6; IR (KBr, cm^{-1}): *v* = 3026, 2933, 2857, 2229, 1757, 1591, 1445, 1252, 1022, 896, 744, 518, 461; HRMS (ESI) calc. $C_{18}H_{17}NNaS$ $[M+Na]^+$: 302.0974, found: 302.0975.

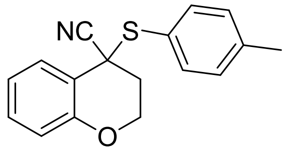

4-(*p*-Tolylthio)chroman-4-carbonitrile (3ta): Yield: 52 mg (92%), yellow oil. ^{1}H NMR (400 MHz, $CDCl_3$, ppm): δ = 7.54 (d, *J* = 8.1 Hz, 2H), 7.43 (dd, *J* = 7.8, 1.5 Hz, 1H), 7.23 (m, 3H), 6.92 (m, 1H), 6.85 (dd, *J* = 8.3, 0.9 Hz, 1H), 4.63 (td, *J* = 11.5, 2.1 Hz, 1H), 4.29 (dt, *J* = 11.5, 3.6 Hz, 1H), 2.44 (m, 1H), 2.40 (d, *J* = 5.5 Hz, 3H), 2.22 (ddd, *J* = 14.5, 3.4, 2.2 Hz, 1H); ^{13}C NMR (101 MHz, $CDCl_3$, ppm): δ =153.5, 141.4, 137.2, 130.9, 130.3, 129.6, 126.1, 121.0, 120.7, 117.8, 117.2, 61.1, 44.1, 31.0, 21.4; IR (KBr, cm^{-1}): *v* = 3050, 2926, 2233, 1688, 1594, 1483, 1231, 1126, 1050, 814, 755, 480; HRMS (ESI) calc. $C_{17}H_{15}NNaOS$ $[M+Na]^+$: 304.0767, found: 304.0770.

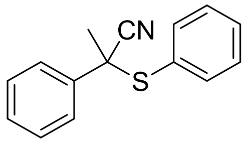

2-Phenyl-2-(phenylthio)propanenitrile (3ab): Yield: 43 mg (90%), yellow oil. ^{1}H NMR (400 MHz, $CDCl_3$, ppm): δ = 7.47 (m, 2H), 7.40 (m, 2H), 7.32 (m, 6H), 2.00 (s, 3H); ^{13}C NMR (101 MHz, $CDCl_3$, ppm): δ = 137.7, 136.7, 130.3, 130.2, 129.0, 128.7, 128.7, 126.2, 120.8, 48.8, 27.5; IR (KBr, cm^{-1}): *v* = 3063, 2926, 2232, 1446, 1378, 1269, 1071, 1025, 751, 693, 510; HRMS (ESI) calc. $C_{15}H_{13}NNaS$ $[M+Na]^+$: 262.0661, found: 262.0667.

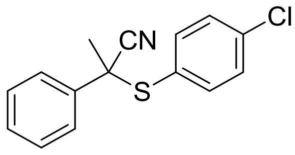

2-((4-Chlorophenyl)thio)-2-phenylpropanenitrile (3ac): Yield: 48 mg (88%), yellow oil. ^{1}H NMR (400 MHz, $CDCl_3$, ppm): δ = 7.44 (m, 2H), 7.32 (m, 5H), 7.24 (m, 2H), 2.01 (s, 3H); ^{13}C NMR (101 MHz, $CDCl_3$, ppm): δ = 137.9, 137.4, 137.0, 129.8, 129.2, 128.8, 128.6, 126.2, 120.6, 49.1, 27.4; IR (KBr, cm^{-1}): *v* = 2924, 1462, 1268, 1083, 1015, 821, 752, 498; HRMS (ESI) calc. $C_{15}H_{12}ClNNaS$ $[M+Na]^+$: 296.0271, found: 296.0271.

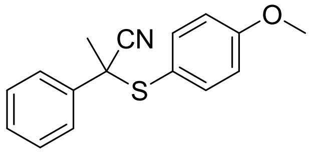

2-((4-Methoxyphenyl)thio)-2-phenylpropanenitrile (3ad): Yield: 50 mg (93%), white solid. M.p.: 72-73 ºC. ^{1}H NMR (400 MHz, $CDCl_3$, ppm): δ = 7.46 (dd, *J* = 8.0, 1.6 Hz, 2H), 7.32 (m, 5H), 6.79 (m, 2H), 3.78 (s, 3H), 1.97 (s, 3H); ^{13}C NMR (101 MHz, $CDCl_3$, ppm): δ = 161.5, 138.5, 137.8, 128.7, 128.6, 126.2, 121.1, 120.9, 114.5, 55.3, 49.1, 27.1; IR (KBr, cm^{-1}): *v* = 2986, 1758, 1587, 1485, 1245, 1174, 1038, 751, 694, 515; HRMS (ESI) calc. $C_{16}H_{15}NNaOS$ $[M+Na]^+$: 292.0767, found: 292.0763.

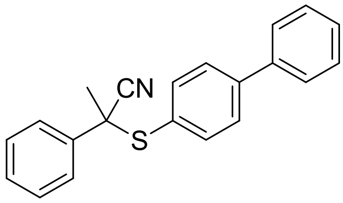

2-([1,1'-Biphenyl]-4-ylthio)-2-phenylpropanenitrile (3ae): Yield: 60 mg (95%), white solid. M.p.: 127-128 ºC. ^{1}H NMR (400 MHz, $CDCl_3$, ppm): δ = 7.50 (m, 10H), 7.35 (ddd, *J* = 14.1, 8.3, 4.5 Hz, 4H), 2.03 (s, 3H); ^{13}C NMR (101 MHz, $CDCl_3$, ppm): δ = 143.1, 139.9, 137.7, 137.1, 129.0, 128.9, 128.7, 128.7, 127.9, 127.6, 127.2, 126.3, 120.9, 49.0, 27.5; IR (KBr, cm^{-1}): *v* = 2920, 2851, 1740, 1644, 1460, 1379, 1258, 1051, 751; HRMS (ESI) calc. $C_{21}H_{17}NNaS$ $[M+Na]^+$: 338.0974, found: 338.0975.

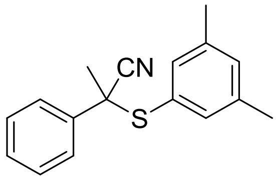

2-((3,5-Dimethylphenyl)thio)-2-phenylpropanenitrile (3af): Yield: 49 mg (92%), white crystal. M.p.: 71-72 ºC. ^{1}H NMR (400 MHz, $CDCl_3$, ppm): δ = 7.47 (m, 2H), 7.32 (m, 3H), 7.00 (s, 3H), 2.23 (d, *J* = 0.4 Hz, 6H), 1.98 (s, 3H); ^{13}C NMR (101 MHz, $CDCl_3$, ppm): δ = 138.5, 137.9, 134.3, 132.0, 129.6, 128.6, 128.6, 126.3, 120.9, 48.6, 27.4, 21.0; IR (KBr, cm^{-1}): *v* = 2924, 2859, 2232, 1594, 1448, 1269, 1067, 850, 751, 691, 508; HRMS (ESI) calc. $C_{17}H_{17}NNaS$ $[M+Na]^+$: 290.0974, found: 290.0971.

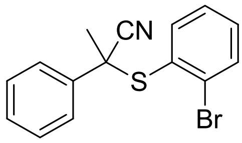

2-((2-Bromophenyl)thio)-2-phenylpropanenitrile (3ag): Yield: 52 mg (82%), yellow oil. ^{1}H NMR (400 MHz, $CDCl_3$, ppm): δ = 7.63 (ddd, J = 14.0, 7.8, 1.5 Hz, 2H), 7.56 (m, 2H), 7.35 (m, 3H), 7.26 (dt, J = 7.6, 3.9 Hz, 1H), 7.19 (td, J = 7.7, 1.7 Hz, 1H), 2.02 (s, 3H); ^{13}C NMR (101 MHz, $CDCl_3$, ppm): δ = 137.6, 137.0, 133.6, 131.9, 131.2, 131.0, 129.0, 128.9, 128.1, 126.3, 120.7, 49.3, 27.7.; IR (KBr, cm^{-1}): *v* = 3061, 2927, 2856, 1573, 1494, 1441, 1257, 1075, 1023, 752, 697, 512; HRMS (ESI) calc. $C_{15}H_{12}BrNNaS$ $[M+Na]^+$: 339.9766, found: 339.9765.

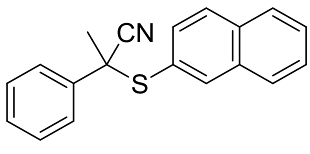

2-(Naphthalen-2-ylthio)-2-phenylpropanenitrile (3ah): Yield: 55 mg (95%), white solid. M.p.: 85-86 ºC. ^{1}H NMR (400 MHz, $CDCl_3$, ppm): δ = 7.95 (s, 1H), 7.76 (m, 3H), 7.50 (m, 4H), 7.41 (dd, J = 8.5, 1.7 Hz, 1H), 7.33 (m, 3H), 2.04 (s, 3H); ^{13}C NMR (101 MHz, $CDCl_3$, ppm): δ = 137.7, 137.3, 133.8, 133.4, 132.5, 128.7, 128.7, 128.5, 128.3, 127.6, 127.5, 127.5, 126.5, 126.3, 120.9, 49.0, 27.5; IR (KBr, cm^{-1}): ν = 2926, 2852, 1762, 1448, 1376, 1246, 1059, 752, 479; HRMS (ESI) calc. $C_{19}H_{15}NNaS$ $[M+Na]^+$: 312.0817, found: 312.0812.

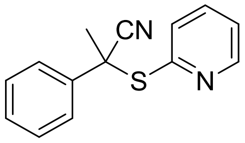

2-Phenyl-2-(pyridin-2-ylthio)propanenitrile (3ai): Yield: 25 mg (53%), yellow oil. ^{1}H NMR (400 MHz, $CDCl_3$, ppm): δ = 8.45 (dd, J = 4.8, 0.9 Hz, 1H), 7.67 (m, 2H), 7.50 (td, J = 7.8, 1.9 Hz, 1H), 7.31 (m, 4H), 7.08 (ddd, J = 7.5, 4.9, 0.8 Hz, 1H), 2.13 (s, 3H); ^{13}C NMR (101 MHz, $CDCl_3$, ppm): δ = 154.9, 149.9, 137.8, 136.8, 128.9, 128.7, 126.2, 125.8, 122.1, 120.9, 47.1, 28.8; IR (KBr, cm^{-1}): ν = 3055, 2991, 2929, 2234, 1570, 1444, 1281, 1215, 1128, 1075, 988, 756, 700, 599, 513; HRMS (ESI) calc. $C_{14}H_{12}N_2NaS$ $[M+Na]^+$: 263.0613, found: 263.0614.

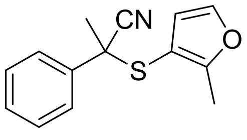

2-((2-Methylfuran-3-yl)thio)-2-phenylpropanenitrile (3aj): Yield: 44 mg (90%), tan oil. ^{1}H NMR (400 MHz, $CDCl_3$, ppm): δ = 7.50 (m, 2H), 7.37 (m, 3H), 7.26 (d, J = 1.9 Hz, 1H), 6.24 (d, J = 1.9 Hz, 1H), 2.16 (s, 3H), 2.04 (s, 3H); ^{13}C NMR (101 MHz, $CDCl_3$, ppm): δ = 159.7, 140.8, 137.8, 128.7, 128.7, 126.2, 121.2, 115.7, 106.9, 48.3, 26.7, 11.6; IR (KBr, cm^{-1}): ν = 2927, 2857, 2234, 1570, 1720, 1581, 1505, 1446, 1382, 1225, 1125, 1081, 941, 696, 507; HRMS (ESI) calc. $C_{14}H_{13}NNaOS$ $[M+Na]^+$: 266.0610, found: 266.0614.

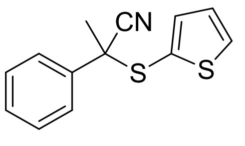

2-Phenyl-2-(thiophen-2-ylthio)propanenitrile (3ak): Yield: 42 mg (86%), yellow oil. ^{1}H NMR (400 MHz, $CDCl_3$, ppm): δ = 7.49 (dt, J = 4.6, 2.5 Hz, 2H), 7.45 (dd, J = 5.4, 1.2 Hz, 1H), 7.35 (m, 3H), 7.23 (dd, J = 3.6, 1.2 Hz, 1H), 7.02 (dd, J = 5.3, 3.6 Hz, 1H), 2.01 (s, 3H); ^{13}C NMR (101 MHz, $CDCl_3$, ppm): δ = 138.4, 137.1, 133.1, 128.9, 128.9, 128.3, 127.9, 126.3, 120.7, 50.6, 26.9; IR (KBr, cm^{-1}): ν = 3092, 2986, 2929, 2233, 1494, 1447, 1393, 1220, 1072, 992, 847, 707, 508; HRMS (ESI) calc. $C_{13}H_{11}NNaS_2$ $[M+Na]^+$: 268.0225, found: 268.0229.

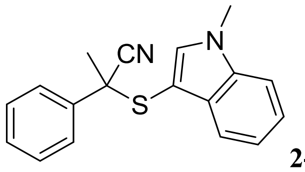

2-((1-Methyl-1H-indol-3-yl)thio)-2-phenylpropanenitrile (3al): Yield: 45 mg (76%), brown solid. M.p.: 121-122 ºC. ^{1}H NMR (400 MHz, $CDCl_3$, ppm): δ = 7.54 (m, 3H), 7.31 (d, J = 7.4 Hz, 5H), 7.24 (d, J = 5.3 Hz, 1H), 7.12 (t, J = 7.4 Hz, 1H), 3.78 (s, 3H), 1.96 (s, 3H); ^{13}C NMR (101 MHz, $CDCl_3$, ppm): δ = 137.9, 137.2, 137.1, 130.7, 128.7, 128.5, 126.3, 122.5, 121.8, 120.8, 119.5, 109.7, 100.2, 50.1, 33.2, 27.0; IR (KBr, cm^{-1}): ν = 3092, 2986, 2929, 2233, 1494, 1447, 1393, 1220, 1072, 992, 847, 707, 508; HRMS (ESI) calc. $C_{18}H_{16}N_2NaS$ $[M+Na]^+$: 315.0926, found: 315.0927.

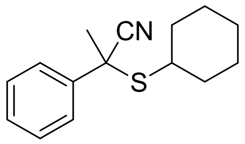

2-(Cyclohexylthio)-2-phenylpropanenitrile (3am): Yield: 21 mg (42%), pale yellow oil. ^{1}H NMR (400 MHz, $CDCl_3$, ppm): δ = 7.65 (dd, *J* = 5.3, 3.4 Hz, 2H), 7.39 (m, 2H), 7.32 (m, 1H), 2.66 (m, 1H), 2.07 (m, 1H), 1.92 (s, 3H), 1.69 (ddd, *J* = 6.6, 3.4, 1.6 Hz, 1H), 1.50 (m, 4H), 1.21 (m, 4H); ^{13}C NMR (101 MHz, $CDCl_3$, ppm): δ = 139.1, 128.8, 128.5, 125.9, 121.6, 45.2, 45.0, 34.0, 33.6, 29.9, 25.7, 25.7, 25.4; IR (KBr, cm^{-1}): *v* = 3062, 2930, 2856, 2232, 1730, 1596, 1491, 1447, 1343, 1269, 1209, 1131, 1076, 1012, 753, 697, 513; HRMS (ESI) calc. $C_{15}H_{19}NNaS$ $[M+Na]^+$: 268.1130, found: 268.1134.

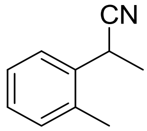

2-(*o*-Tolyl)propanenitrile (3ua): Yield: 26 mg (91%), yellow oil. ^{1}H NMR (400 MHz, $CDCl_3$, ppm): δ = 7.48 (d, *J* = 7.0 Hz, 1H), 7.28 (dd, *J* = 12.3, 7.1 Hz, 2H), 7.22 (t, *J* = 5.9 Hz, 1H), 4.07 (q, *J* = 7.2 Hz, 1H), 2.39 (s, 3H), 1.64 (d, *J* = 7.2 Hz, 3H); ^{13}C NMR (101 MHz, $CDCl_3$, ppm): δ = 135.3, 134.8, 131.0, 128.1, 127.0, 126.7, 121.8, 28.2, 20.0, 19.0; IR (KBr, cm^{-1}): *v* = 2984, 2932, 2883, 2241, 1457, 1297, 1230, 985, 752; HRMS (ESI) calc. $C_{10}H_{11}NNa$ $[M+Na]^+$: 168.0784, found: 168.0781.

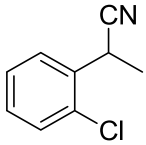

2-(*o*-Chlorophenyl)propanenitrile (3va): Yield: 31 mg (95%), yellow oil. ^{1}H NMR (400 MHz, $CDCl_3$, ppm): δ = 7.61 (d, *J* = 7.5 Hz, 1H), 7.42 (d, *J* = 7.7 Hz, 1H), 7.33 (dd, *J* = 15.4, 7.5 Hz, 2H), 4.38 (q, *J* = 6.7 Hz, 1H), 1.65 (d, *J* = 7.0 Hz, 3H); ^{13}C NMR (101 MHz, $CDCl_3$, ppm): δ = 134.8, 132.5, 130.0, 129.5, 128.2, 127.8, 121.0, 28.9, 19.9; IR (KBr, cm^{-1}): *v* = 2925, 2855, 2244, 1675, 1454, 1305, 1034, 752; HRMS (ESI) calc. C_9H_8ClNNa $[M+Na]^+$: 188.0237, found: 188.0233.

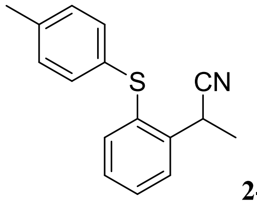

2-(2-(*p*-Tolylthio)phenyl)propanenitrile (3wa): Yield: 20 mg (39%), red-brown oil. ^{1}H NMR (400 MHz, $CDCl_3$, ppm): δ = 7.64 (dd, *J* = 7.7, 1.2 Hz, 1H), 736 (m, 2H), 7.29 (m, 2H), 7.15 (s, 3H), 4.58 (q, *J* = 7.2 Hz, 1H), 2.35 (s, 3H), 1.60 (d, *J* = 7.2 Hz, 3H); ^{13}C NMR (101 MHz, $CDCl_3$, ppm): δ = 138.6, 137.4, 133.7, 133.6, 131.4, 130.5, 130.3, 129.0, 128.7, 127.8, 121.8, 29.0, 21.0; IR (KBr, cm^{-1}): *v* =3058, 2926, 2238, 1645, 1472, 1270, 809, 749; HRMS (ESI) calc. $C_{16}H_{15}NNaS$ $[M+Na]^+$: 276.0817, found:276.0820.

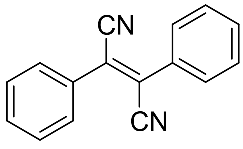

2,3-Diphenylfumaronitrile (3xa): Yield: 31 mg (68%), pale yellow oil. ^{1}H NMR (400 MHz, $CDCl_3$, ppm): δ = 7.84 (m, 4H), 7.55 (dd, *J* = 5.1, 1.9 Hz, 6H).; ^{13}C NMR (101 MHz, $CDCl_3$, ppm): δ = 132.0, 131.7, 129.3, 128.7, 125.6, 116.7; IR (KBr, cm^{-1}): *v* = 2919, 1759, 1445, 1376, 1243, 1056, 753, 688; HRMS (ESI) calc. $C_{16}H_{10}N_2Na$ $[M+Na]^+$: 253.0736, found: 253.0741.

5. Reference

[1] (a) J.-S. Poh, D. N. Tran, C. Battilocchio, J. M. Hawkins, S. V. Ley, *Angew. Chem. Int. Ed.*, 2015, **54**, 7920; (b) J.-S. Poh, S. Makai, T. von Keutz, D. N. Tran, C. Battilocchio, P. Pasau, S. V. Ley, *Angew. Chem. Int. Ed.*, 2017, **56**, 1864.

6. X-ray Diffraction Parameters and Data for 3na

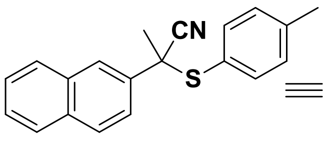

3na

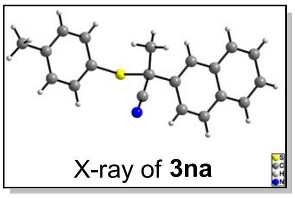

Compound	**3na**
Empirical formula	$C_{20}H_{17}NS$
Formula weight	303.41
Temperature (K)	150.00(10)
Wavelength (Å)	1.54184
Crystal system	monoclinic
Space group	*P2*$P2_1/c$
	a = 11.18234(17) Å α = 90.00 º<br>b = 5.96601(10) Å β = 94.8227(14) º<br>c = 23.6894(4) Å γ = 90.00 º
Volume (Å^3)	1574.82(4)
Z	4
Density (calcd g cm^{-3})	1.280
Absorption coeff. (mm^{-1})	1.766
F(000)	640
Crystal size (mm)	0.39 × 0.34 × 0.13
Crystal color and shape	yellow block
θ range for data collection	5.2090 to 73.7230
Limiting indices	-13 ≤ h ≤ 13, -6 ≤ k ≤ 7, -28 ≤ l ≤ 16
Reflections collected	5794
Unique	3098 [R_{int} = 0.0223]
Refinement method	Full-matrix least-squares on F^2
Data/restraints/parameters	3098/0/201
Goodness-of-fit on F^2	1.073
Final R indexes [I>=2σ (I)]	R_1 = 0.0369, wR_2 = 0.0976
R indexes (all data)	R_1 = 0.0389, wR_2 = 0.0999

7. NMR Spectra for All Compounds

^{1}H NMR (400 MHz, CDCl$_3$) spectrum of compound 3aa

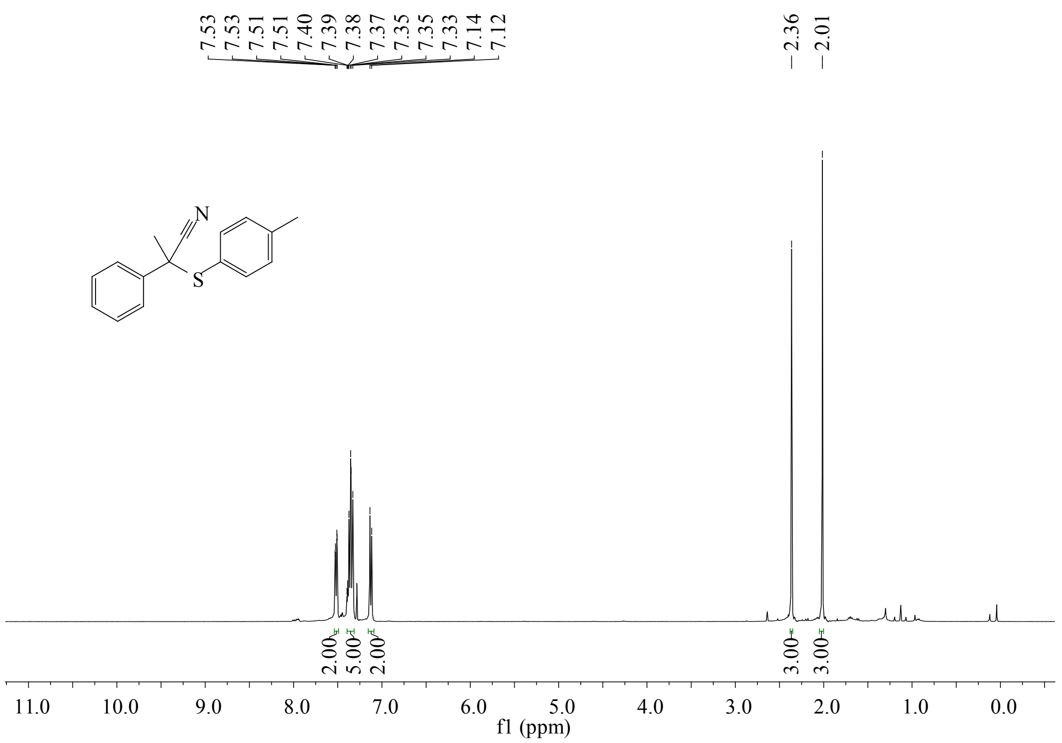

^{13}C NMR (101 MHz, CDCl$_3$) spectrum of compound 3aa

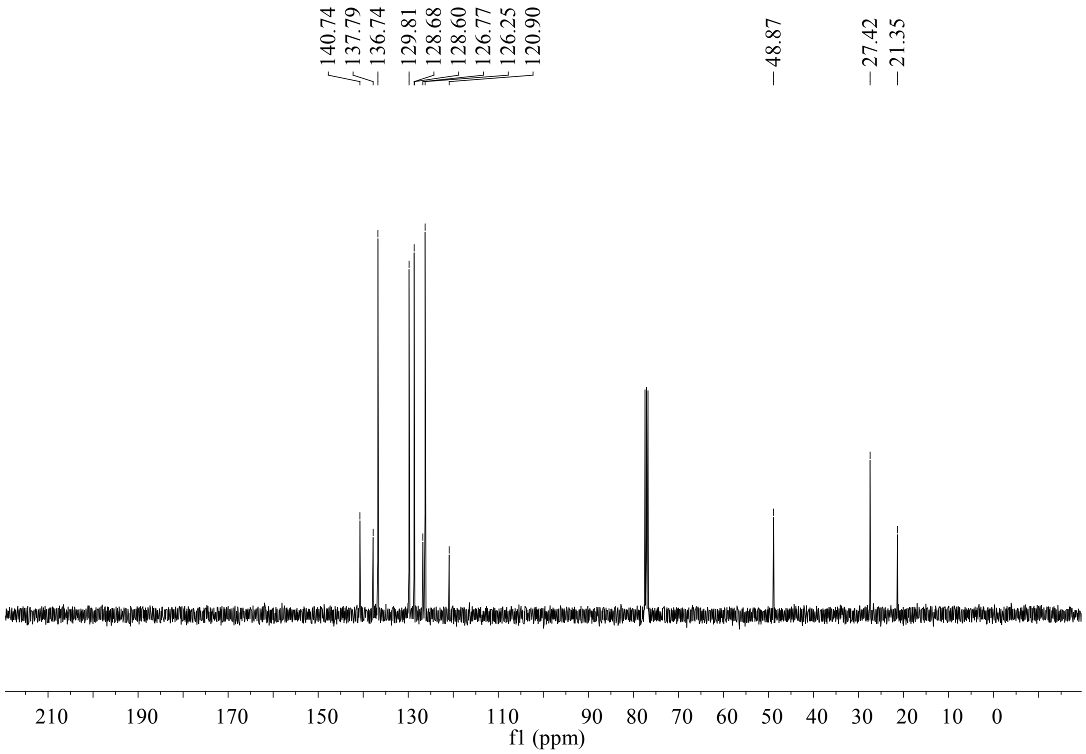

^{1}H NMR (400 MHz, $CDCl_3$) spectrum of compound 3ba

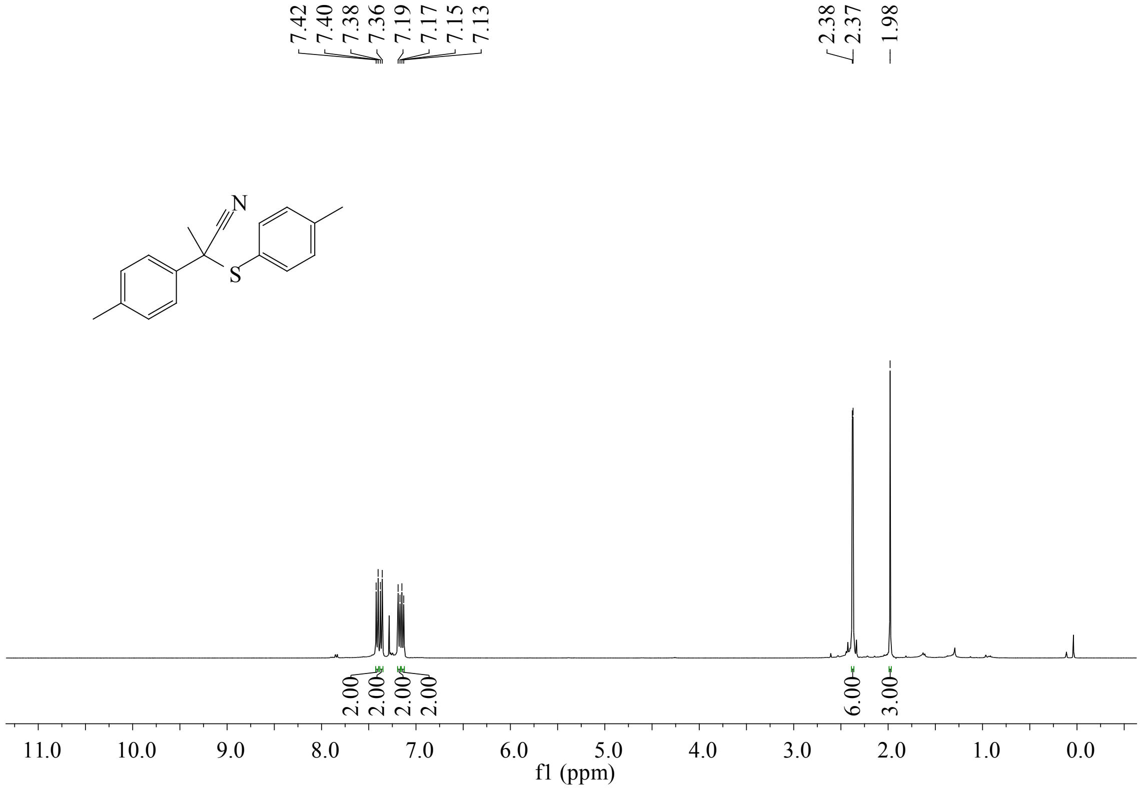

^{13}C NMR (101 MHz, $CDCl_3$) spectrum of compound 3ba

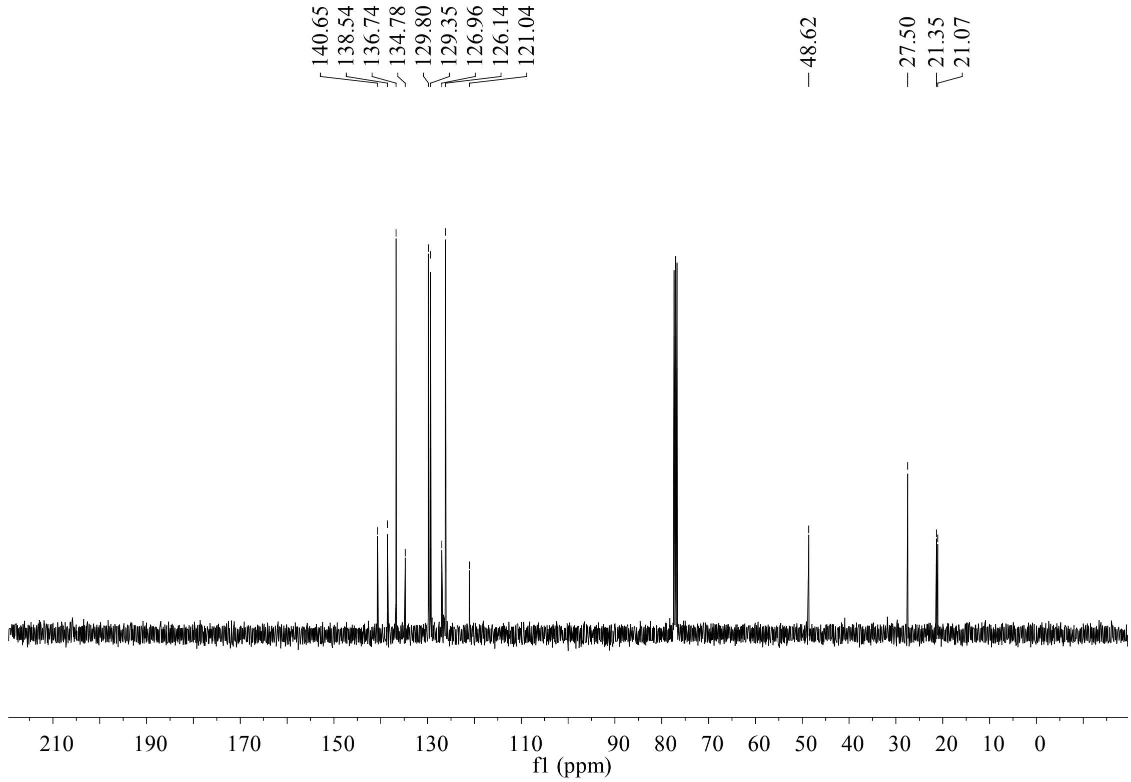

^{1}H NMR (400 MHz, CDCl$_3$) spectrum of compound 3ca

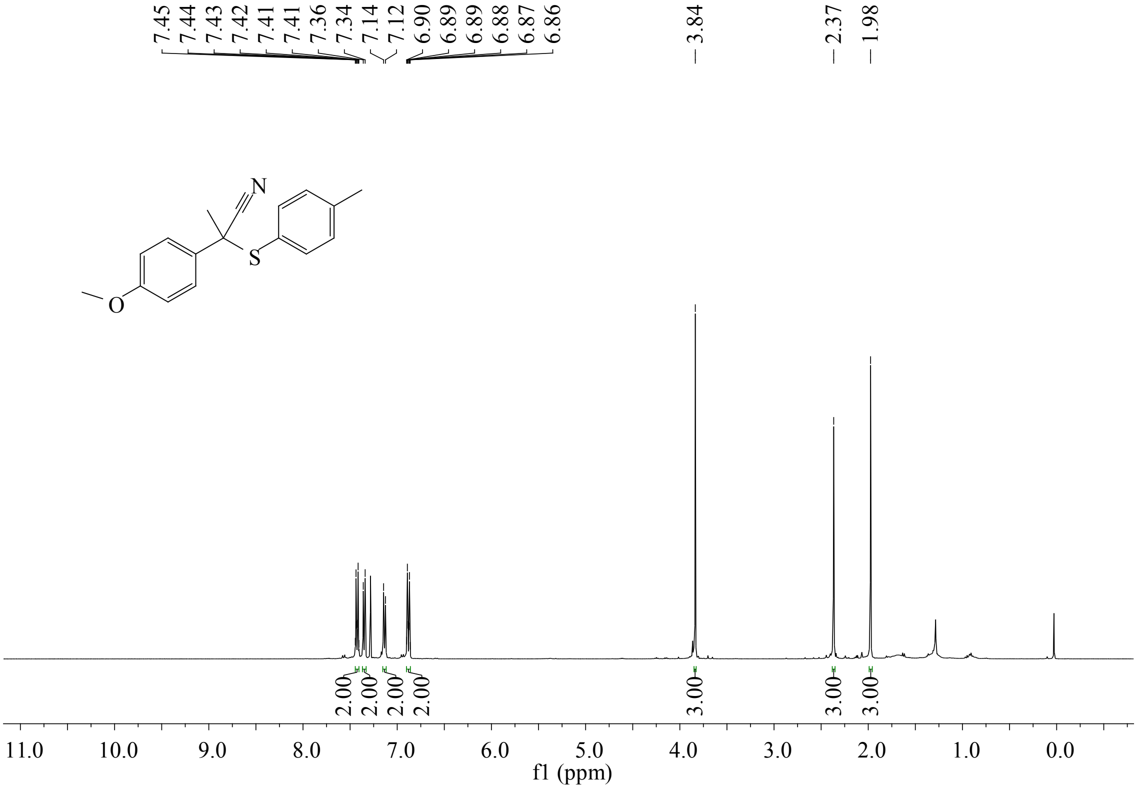

^{13}C NMR (101 MHz, CDCl$_3$) spectrum of compound 3ca

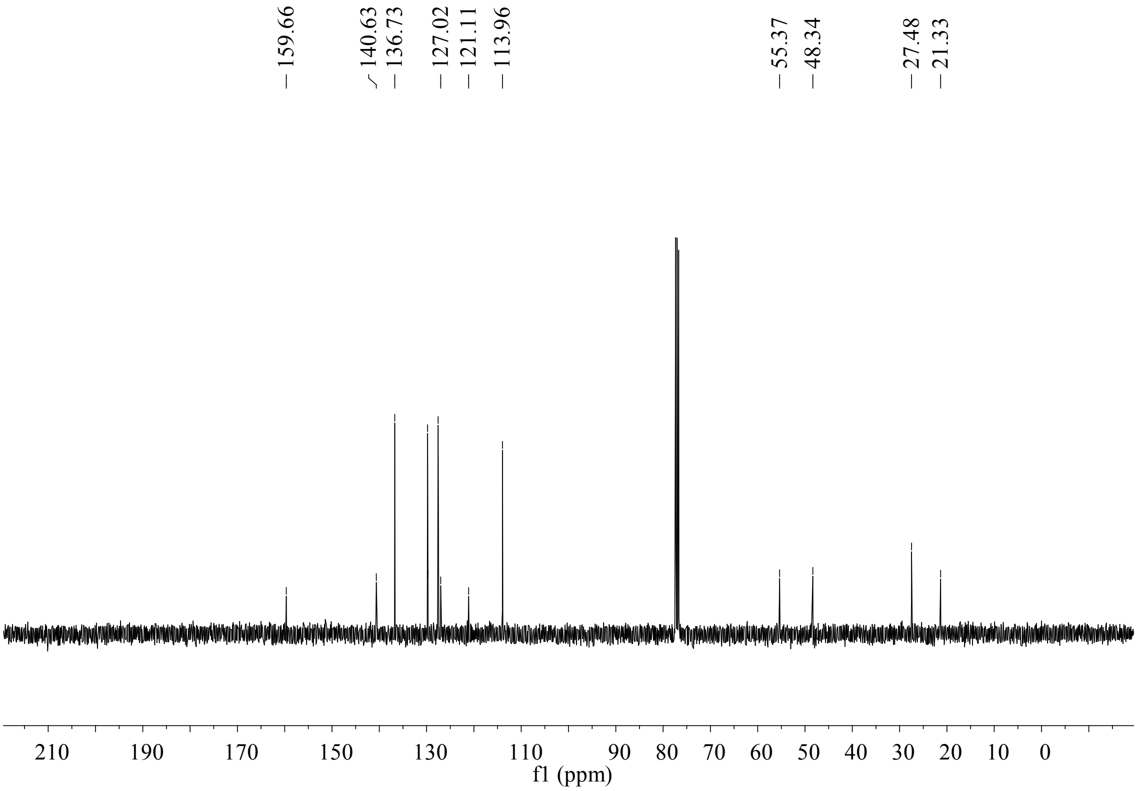

^{1}H NMR (400 MHz, $CDCl_3$) spectrum of compound 3da

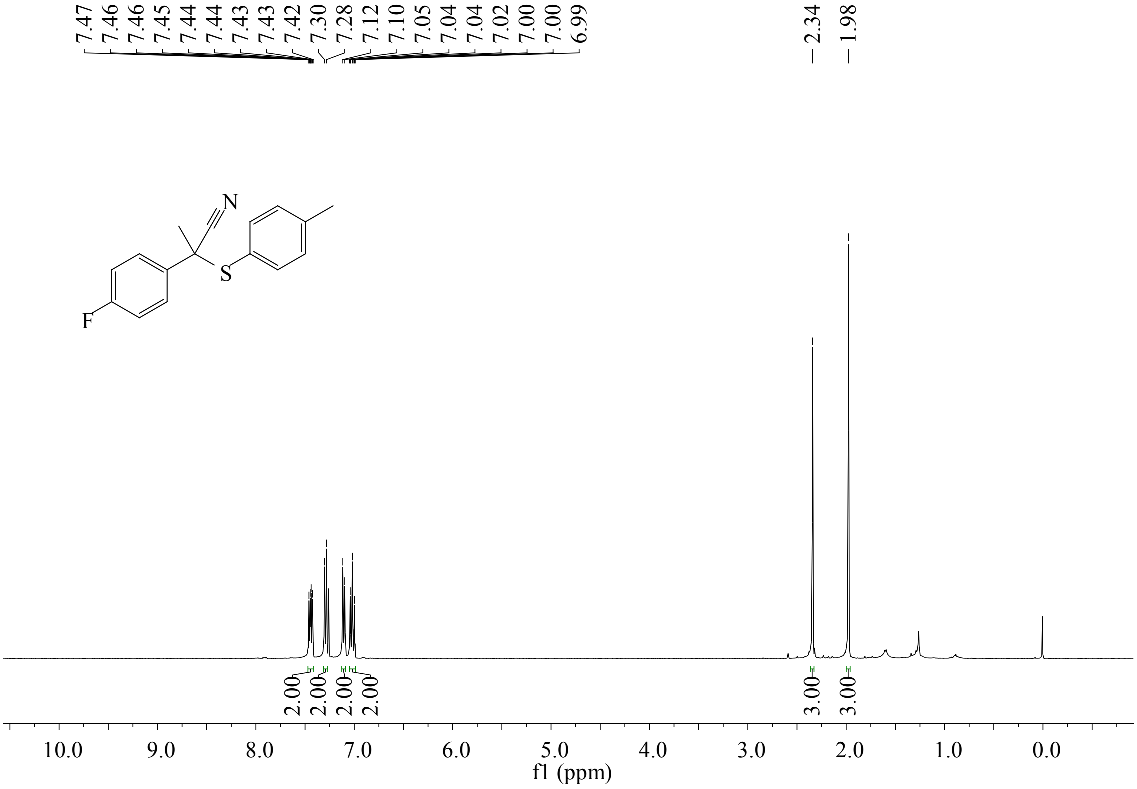

^{13}C NMR (101 MHz, $CDCl_3$) spectrum of compound 3da

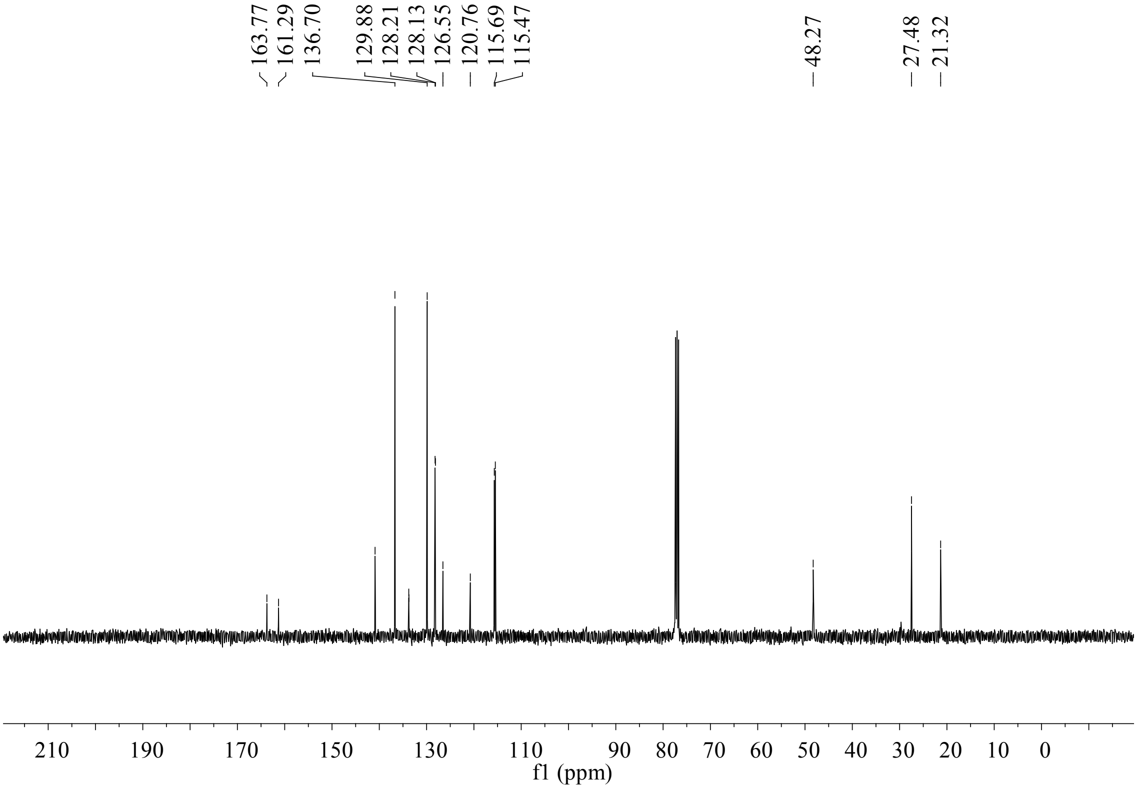

^{1}H NMR (400 MHz, $CDCl_3$) spectrum of compound 3ea

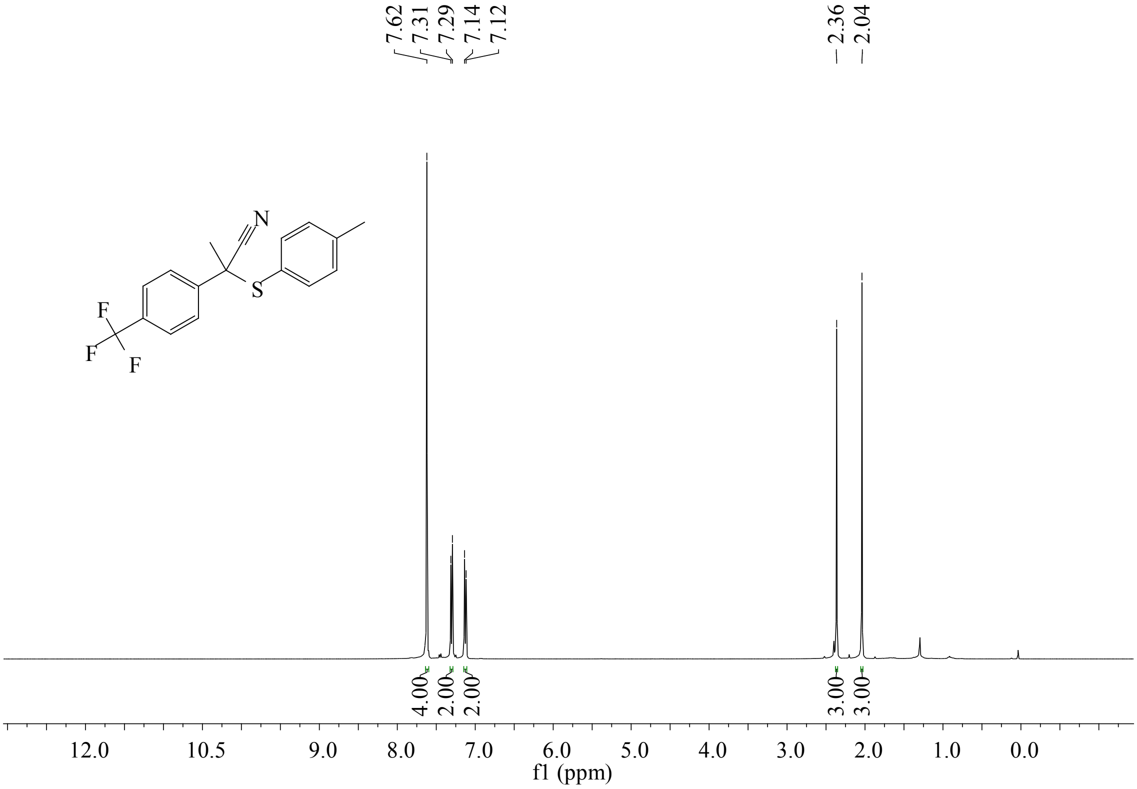

^{13}C NMR (101 MHz, $CDCl_3$) spectrum of compound 3ea

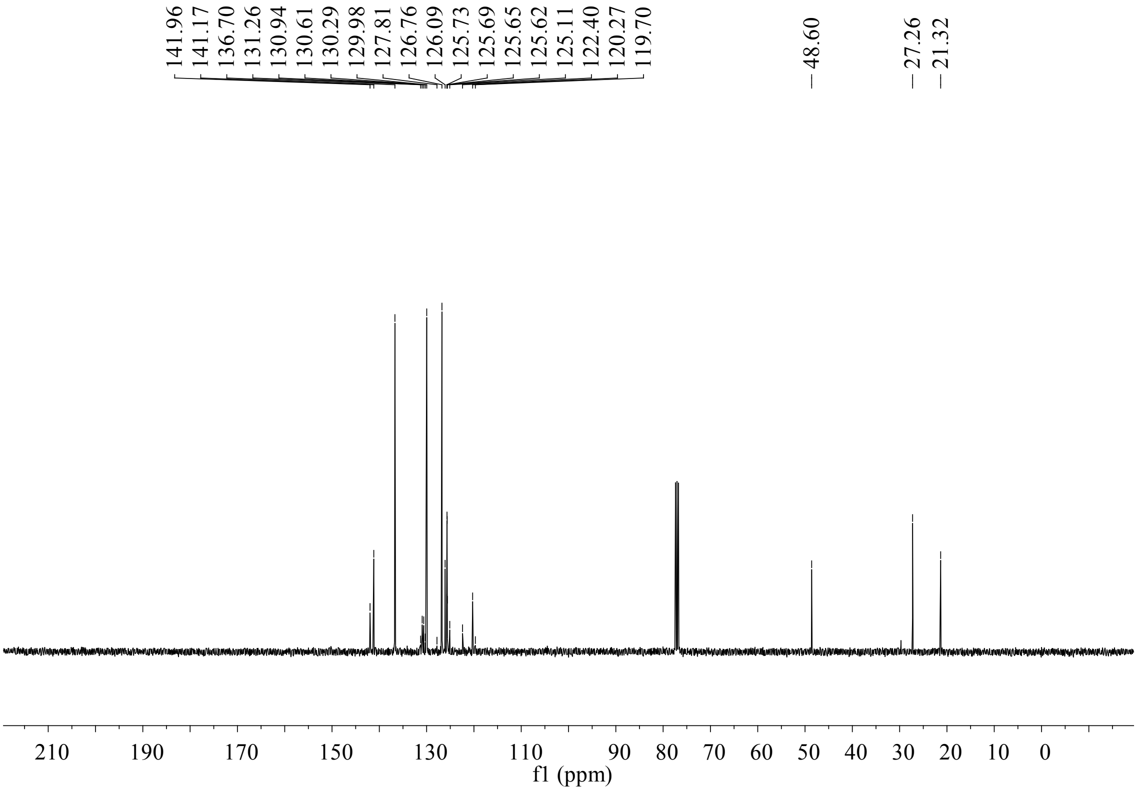

^{1}H NMR (400 MHz, $CDCl_3$) spectrum of compound 3fa

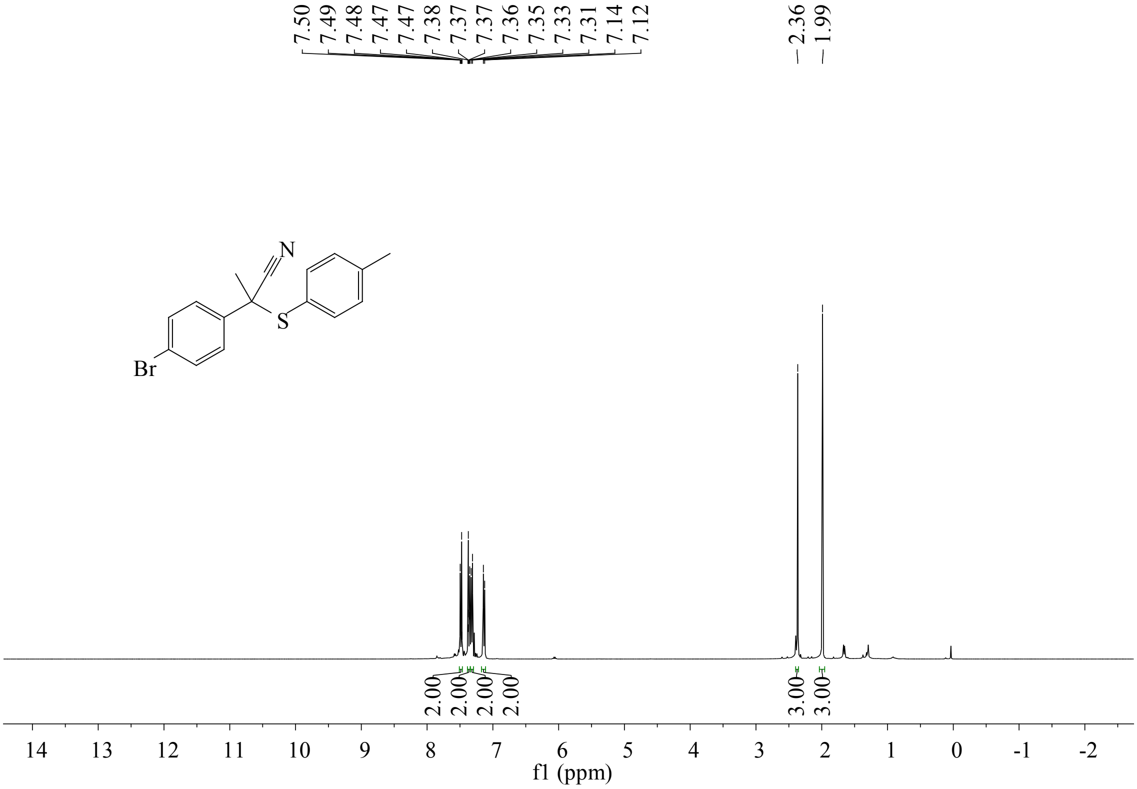

^{13}C NMR (101 MHz, $CDCl_3$) spectrum of compound 3fa

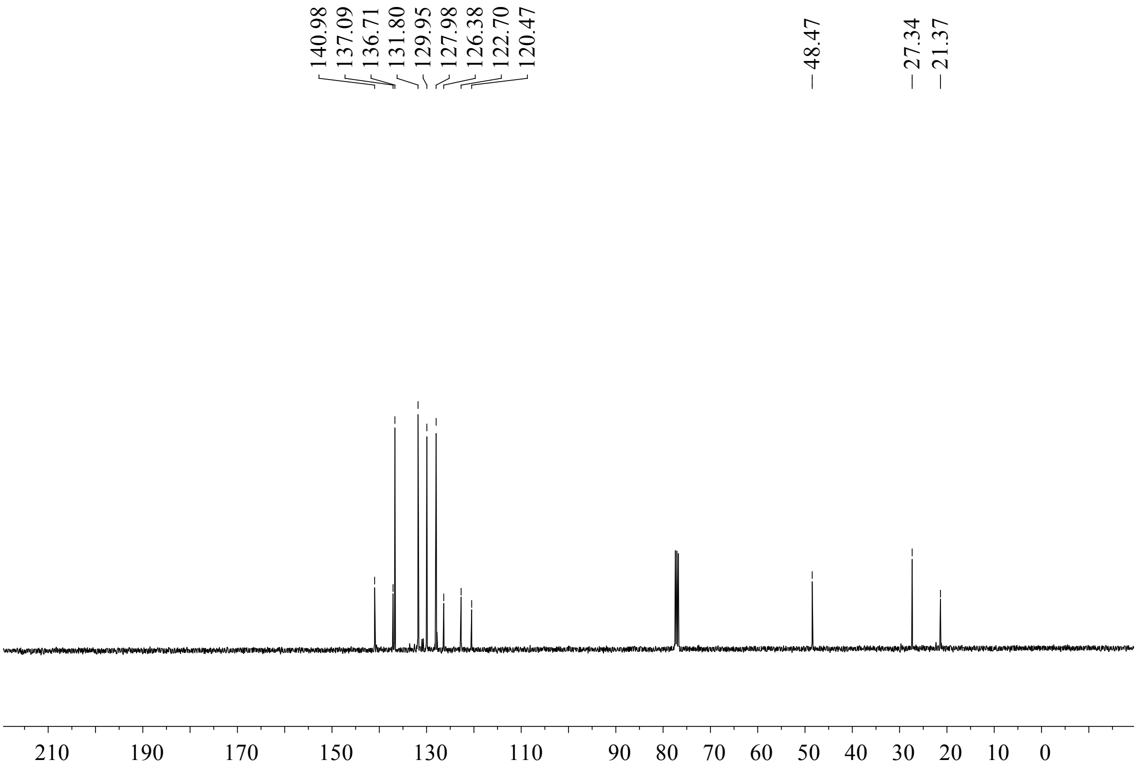

^{1}H NMR (400 MHz, $CDCl_3$) spectrum of compound 3ga

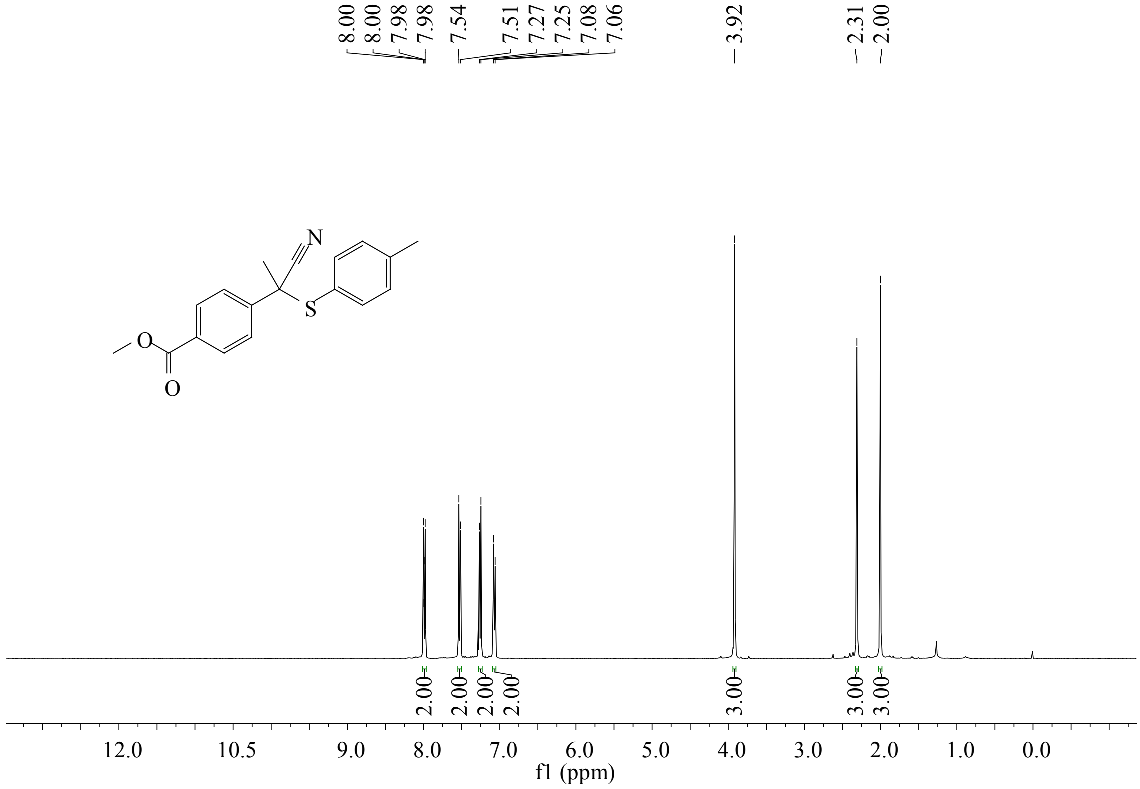

^{13}C NMR (101 MHz, $CDCl_3$) spectrum of compound 3ga

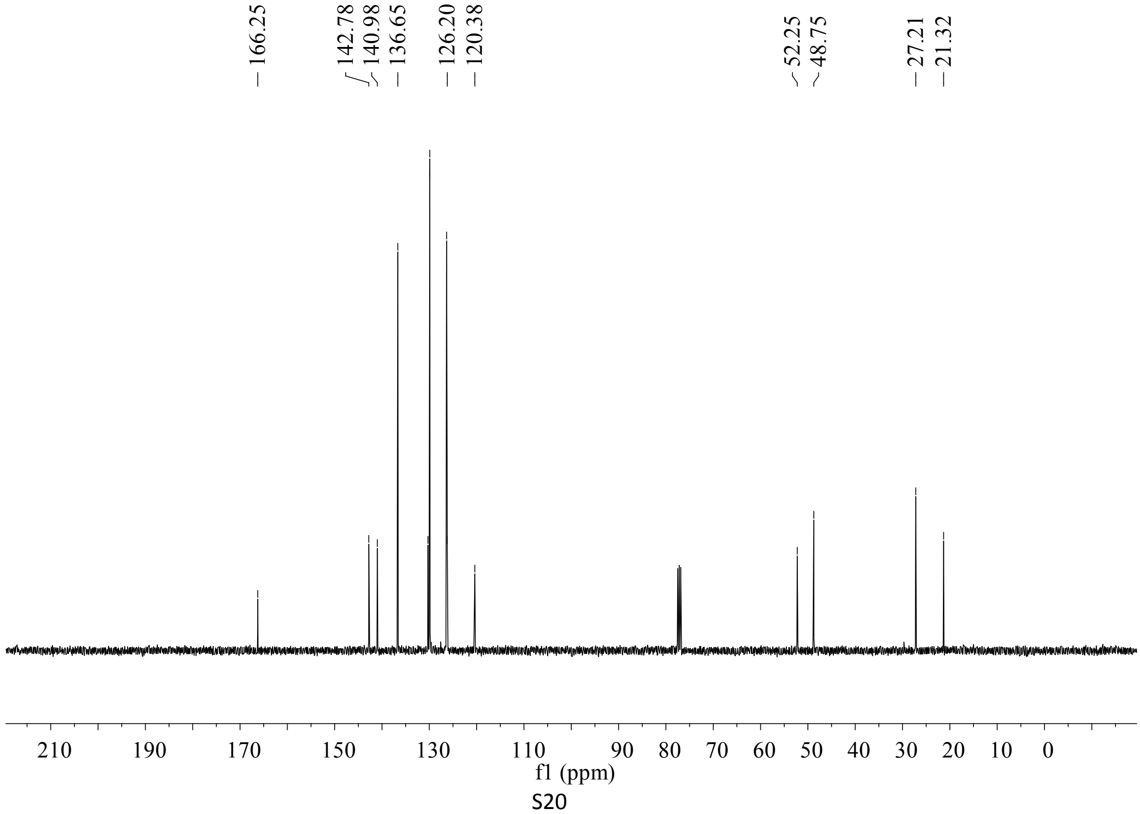

^{1}H NMR (400 MHz, $CDCl_3$) spectrum of compound 3ha

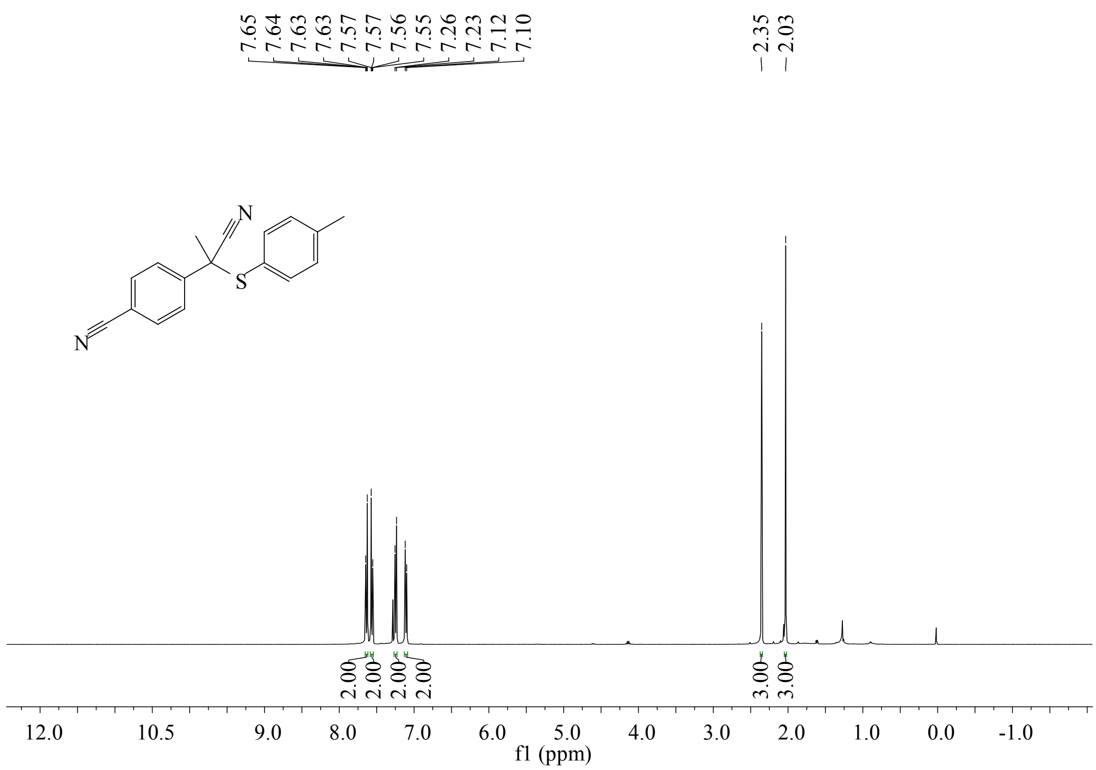

^{13}C NMR (101 MHz, $CDCl_3$) spectrum of compound 3ha

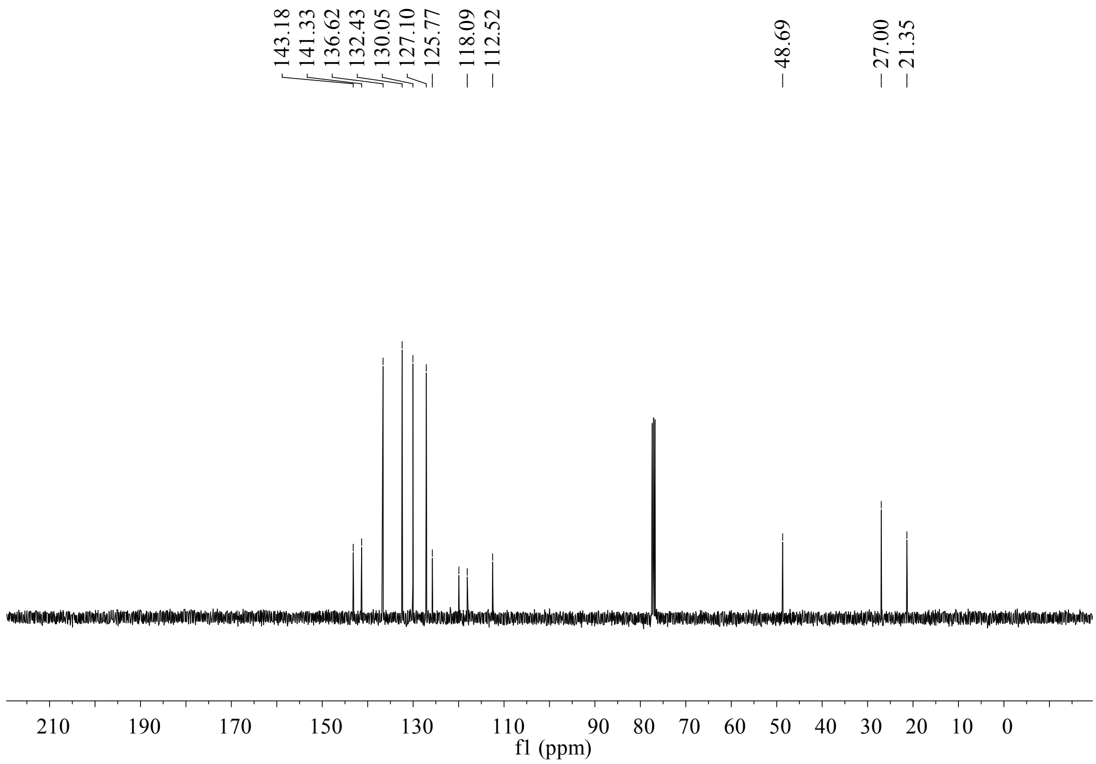

^{1}H NMR (400 MHz, $CDCl_3$) spectrum of compound 3ia

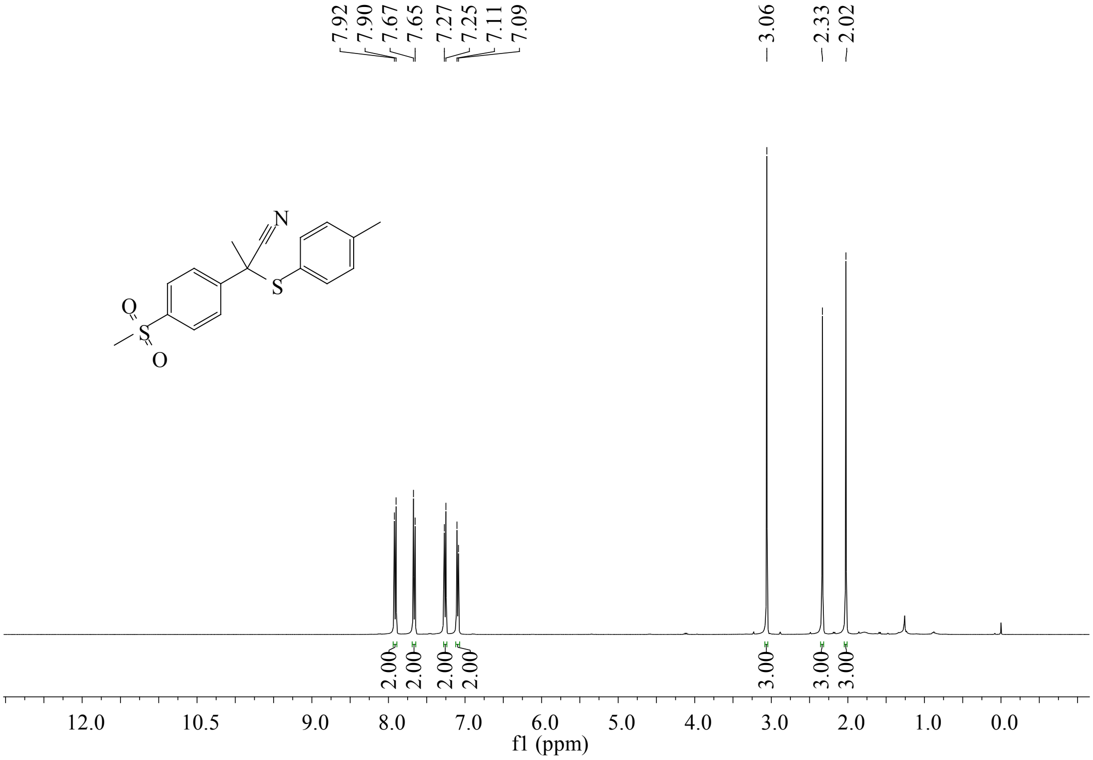

^{13}C NMR (101 MHz, $CDCl_3$) spectrum of compound 3ia

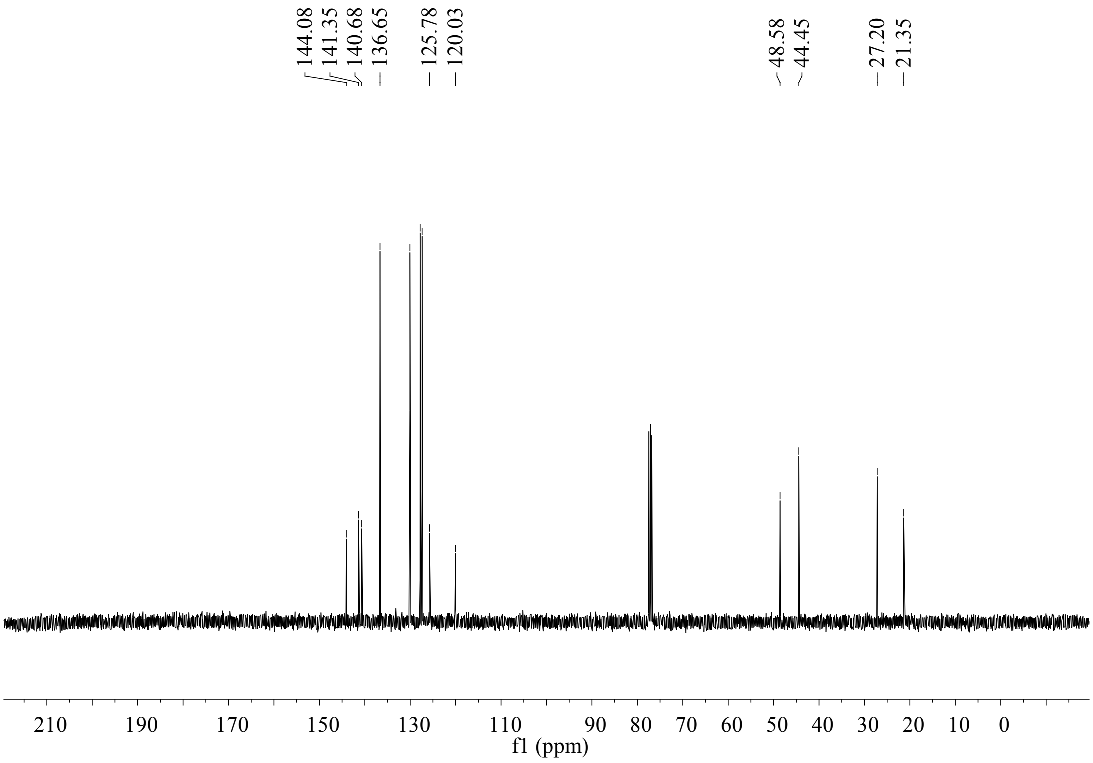

^{1}H NMR (400 MHz, $CDCl_3$) spectrum of compound 3ja

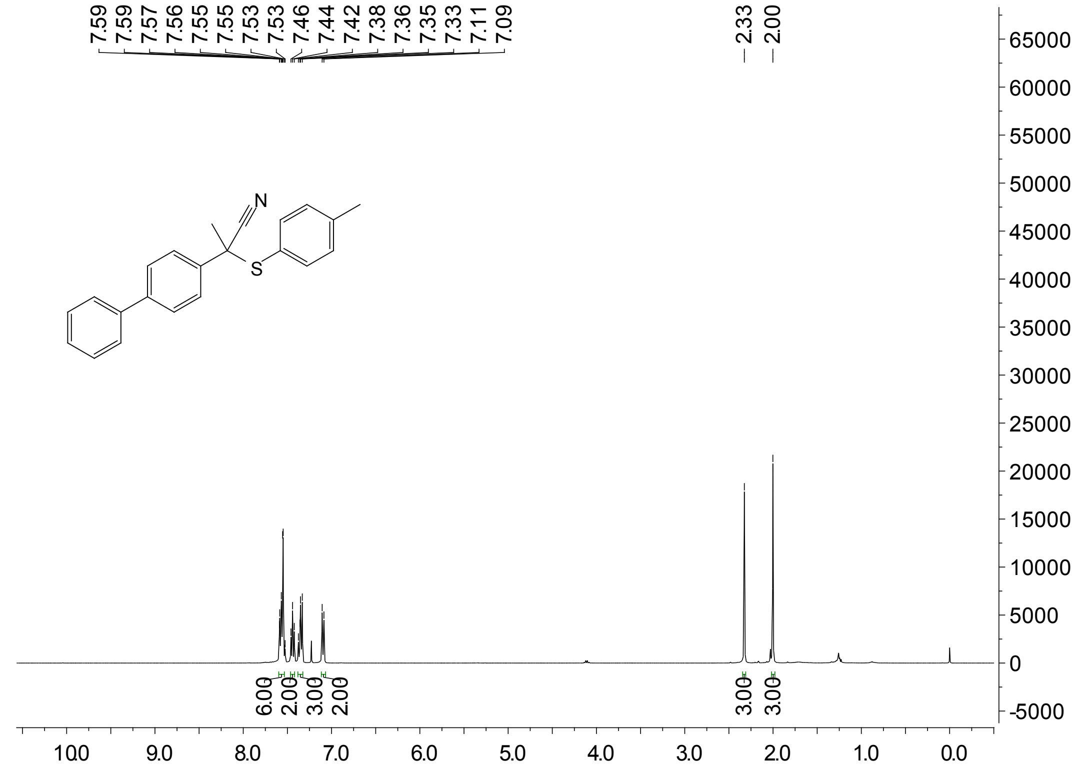

^{13}C NMR (101 MHz, $CDCl_3$) spectrum of compound 3ja

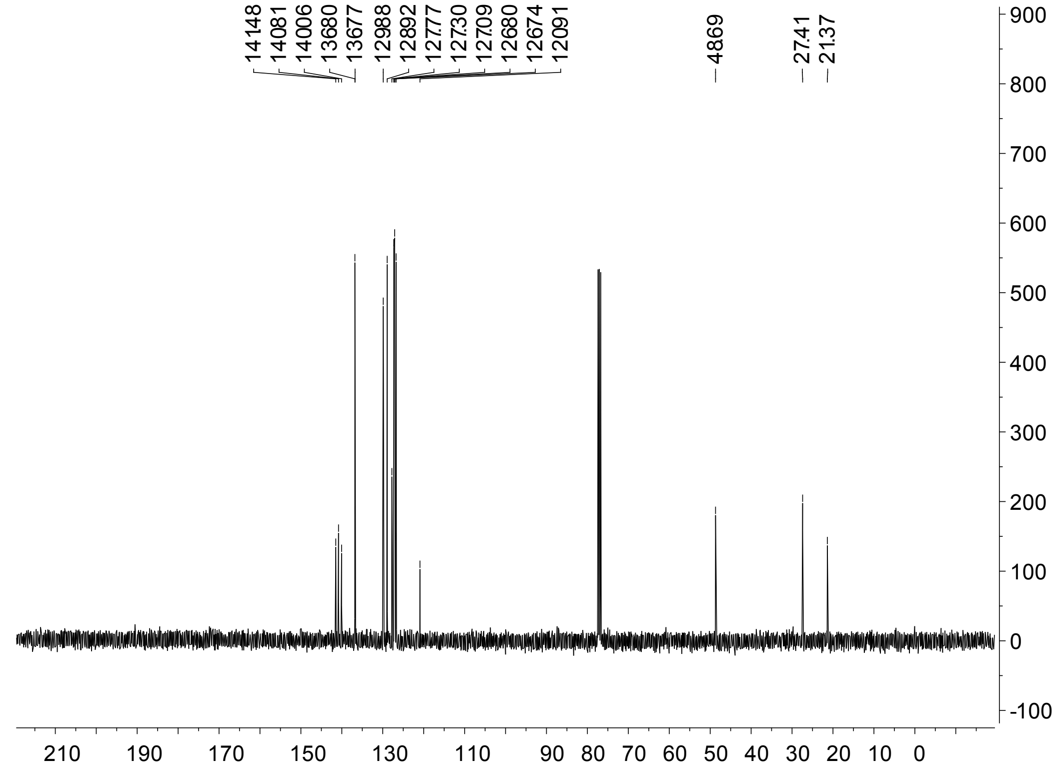

^{1}H NMR (400 MHz, $CDCl_3$) spectrum of compound 3ka

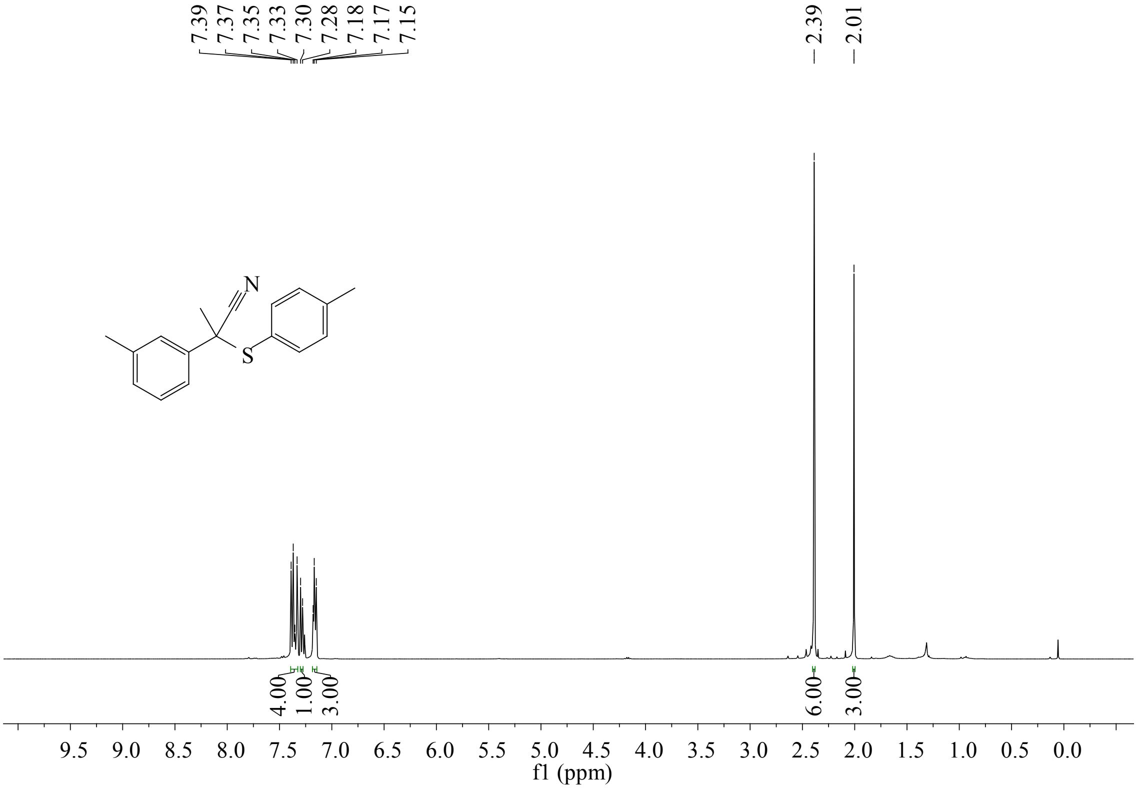

^{13}C NMR (101 MHz, $CDCl_3$) spectrum of compound 3ka

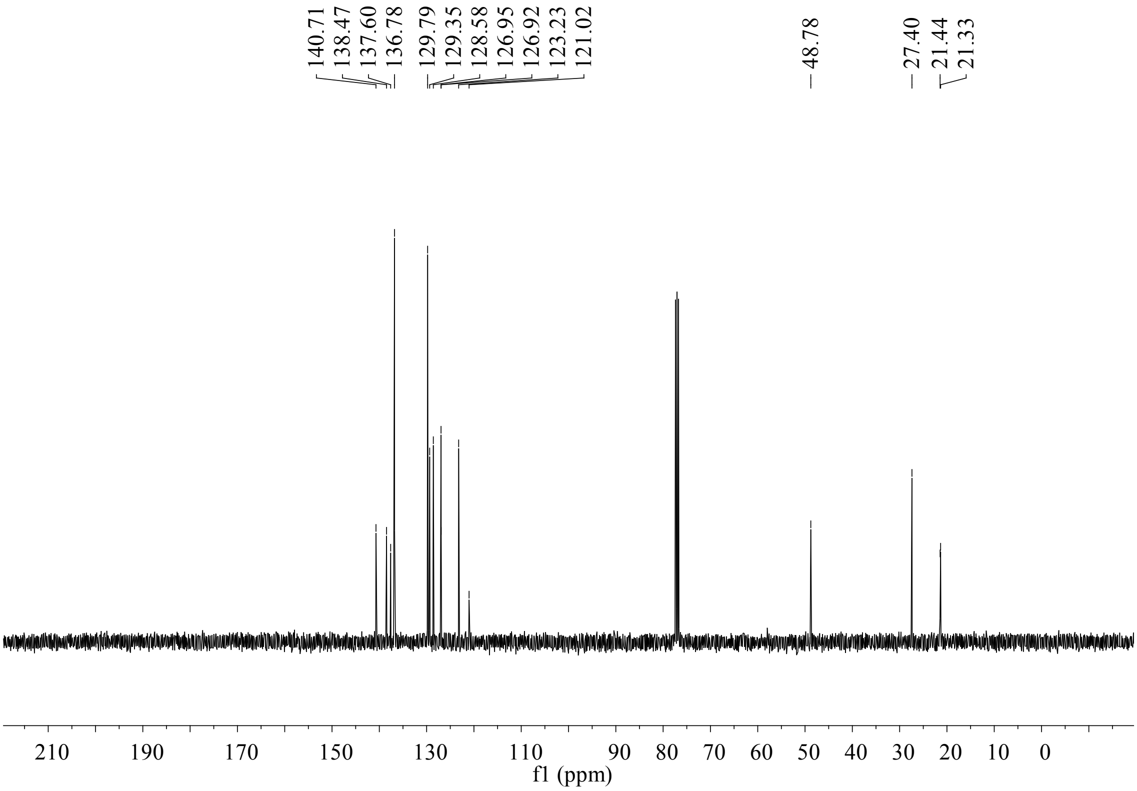

^{1}H NMR (400 MHz, CDCl$_3$) spectrum of compound 3la

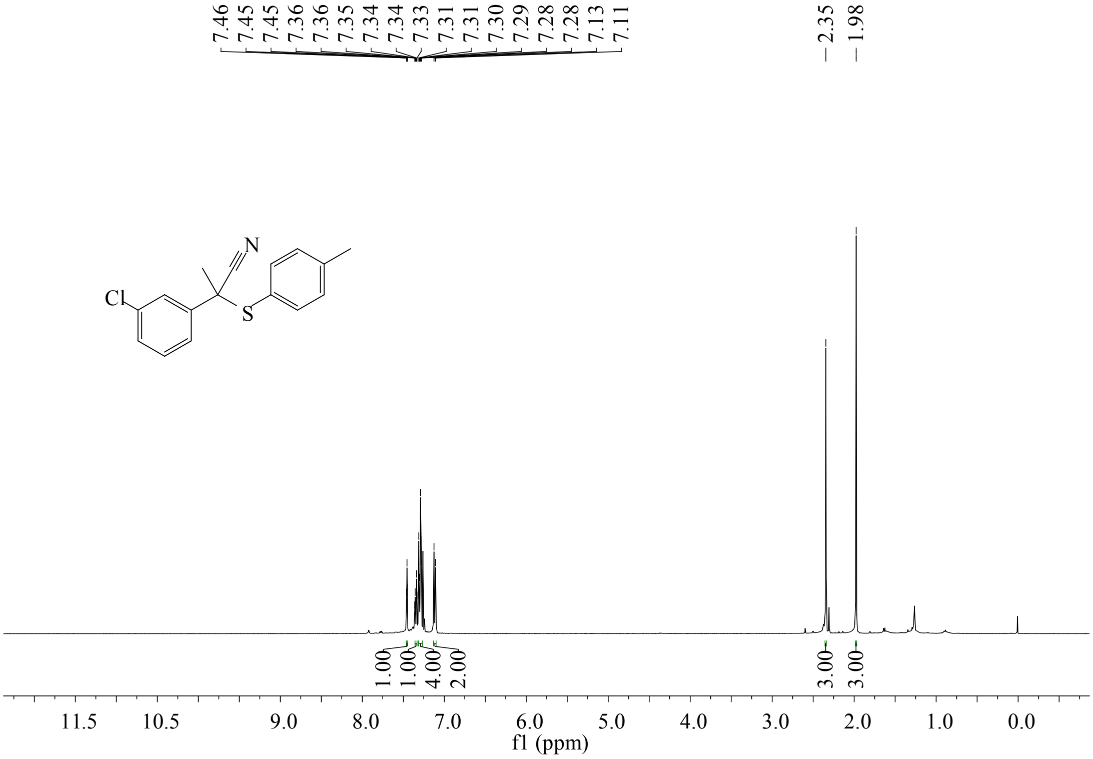

^{13}C NMR (101 MHz, CDCl$_3$) spectrum of compound 3la

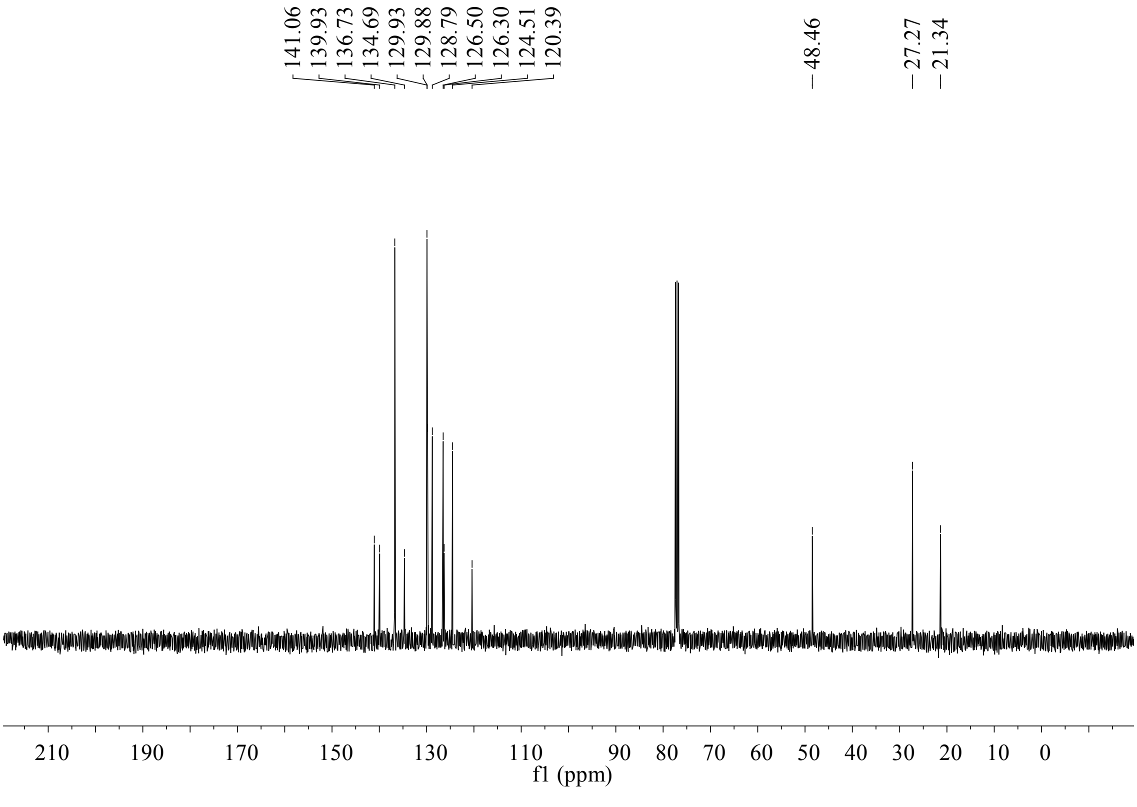

^{1}H NMR (400 MHz, $CDCl_3$) spectrum of compound 3ma

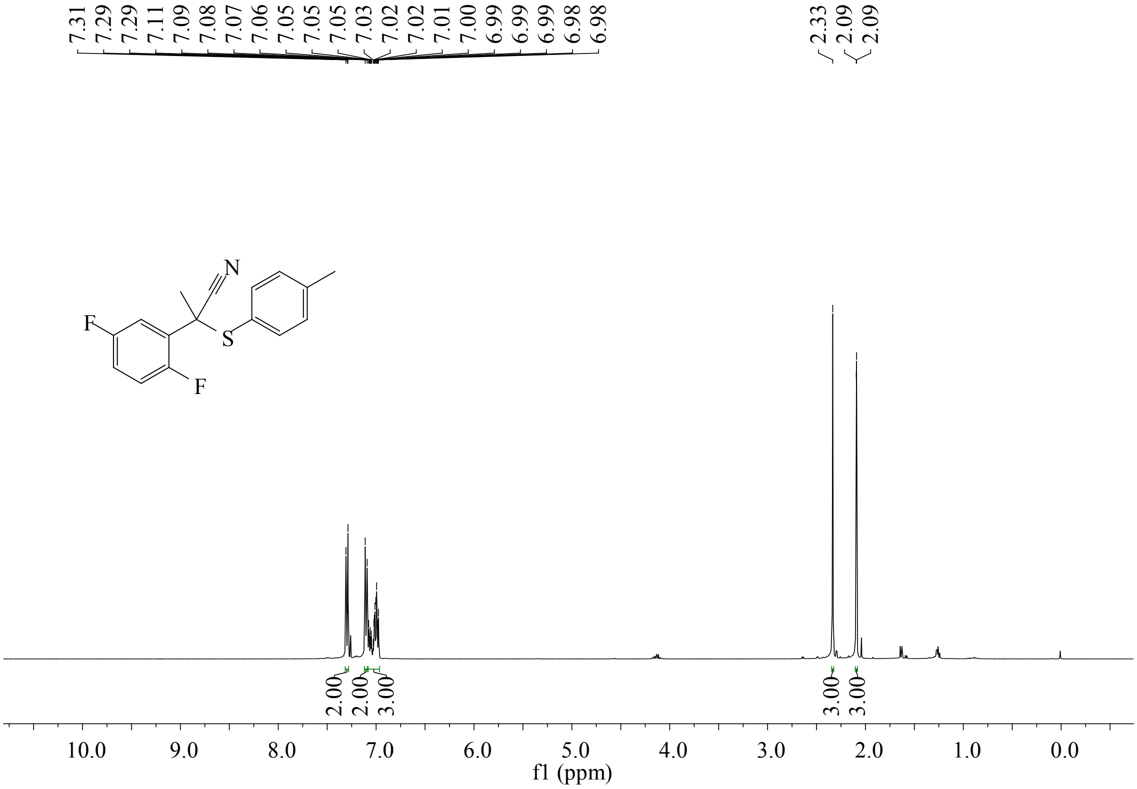

^{13}C NMR (101 MHz, $CDCl_3$) spectrum of compound 3ma

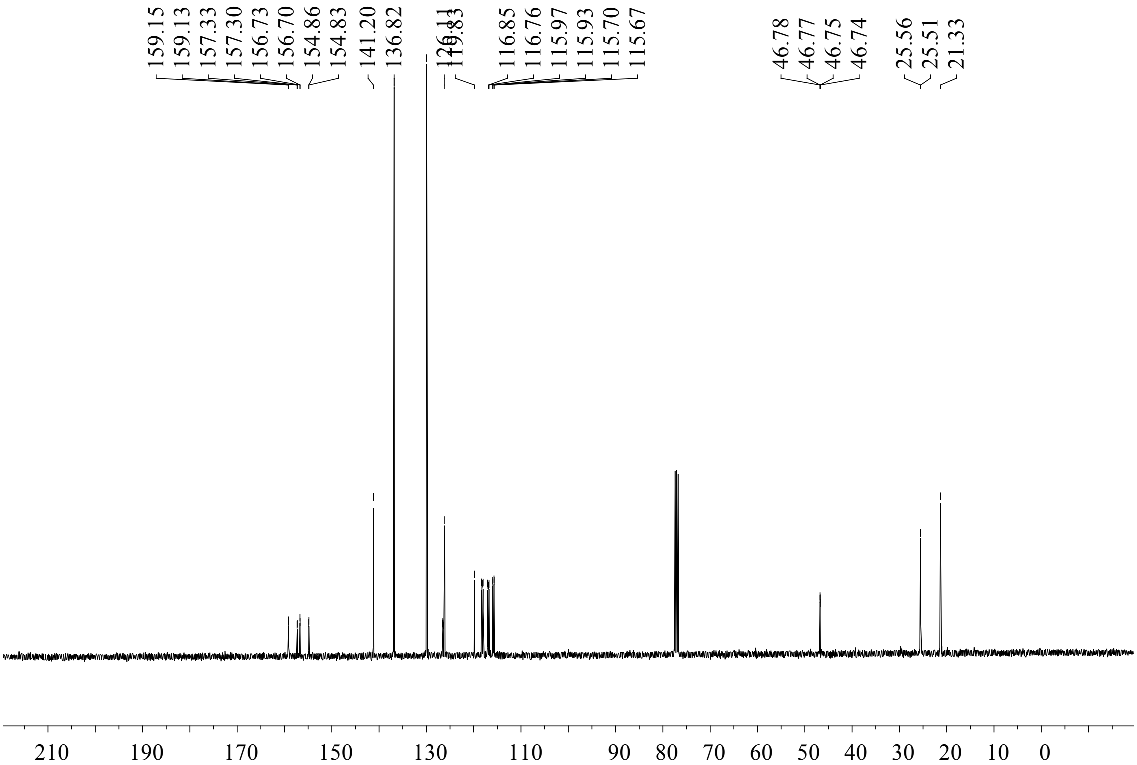

^{1}H NMR (400 MHz, $CDCl_3$) spectrum of compound 3na

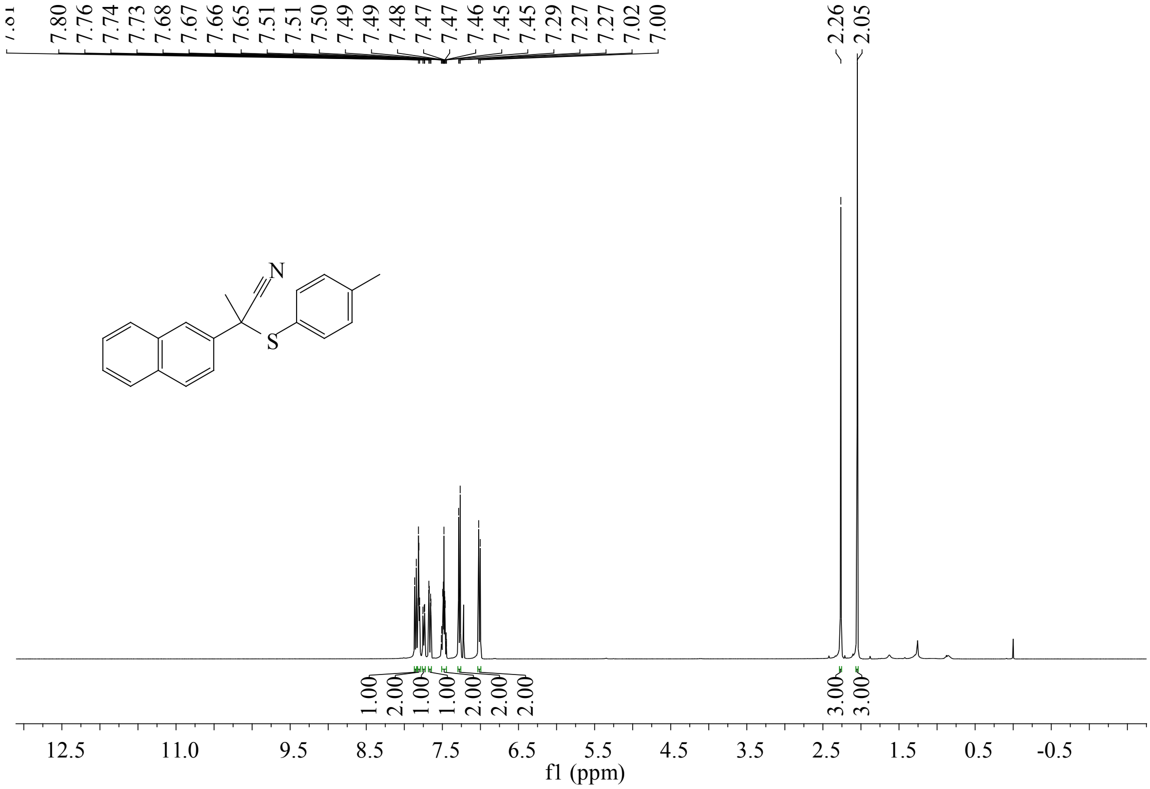

^{13}C NMR (101 MHz, $CDCl_3$) spectrum of compound 3na

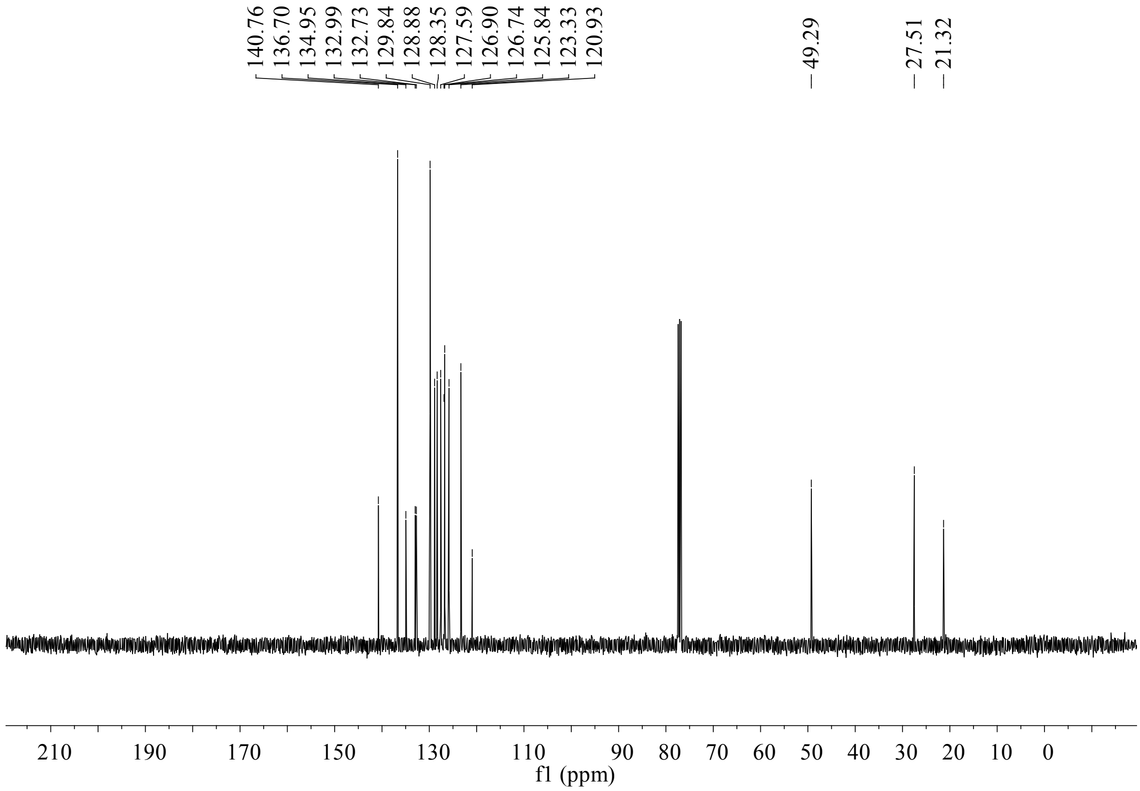

^{1}H NMR (400 MHz, CDCl$_3$) spectrum of compound 3oa

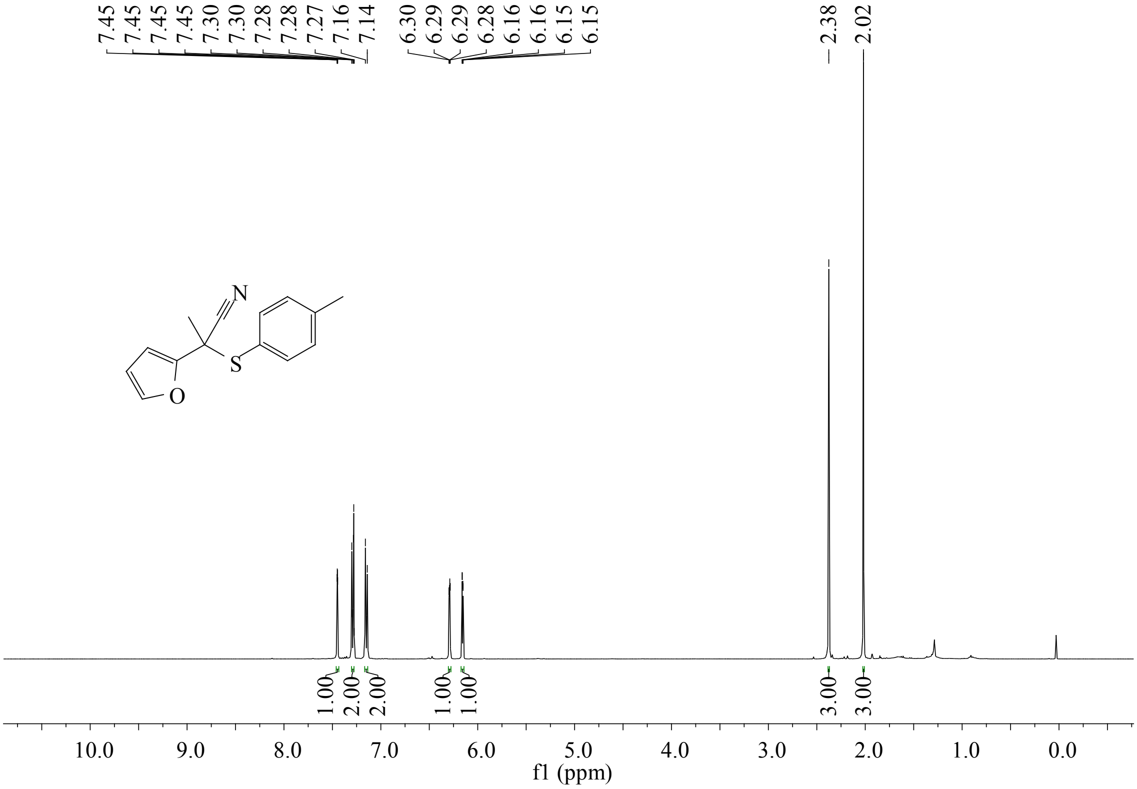

^{13}C NMR (101 MHz, CDCl$_3$) spectrum of compound 3oa

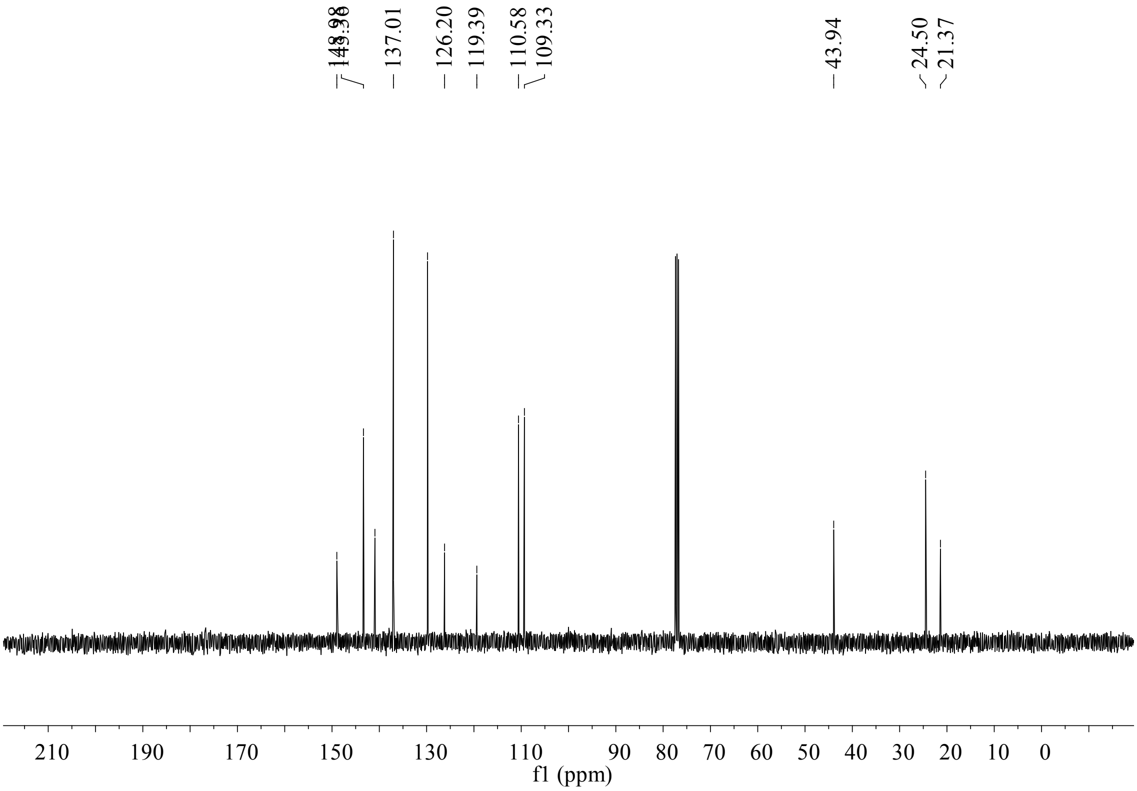

^{1}H NMR (400 MHz, $CDCl_3$) spectrum of compound 3pa

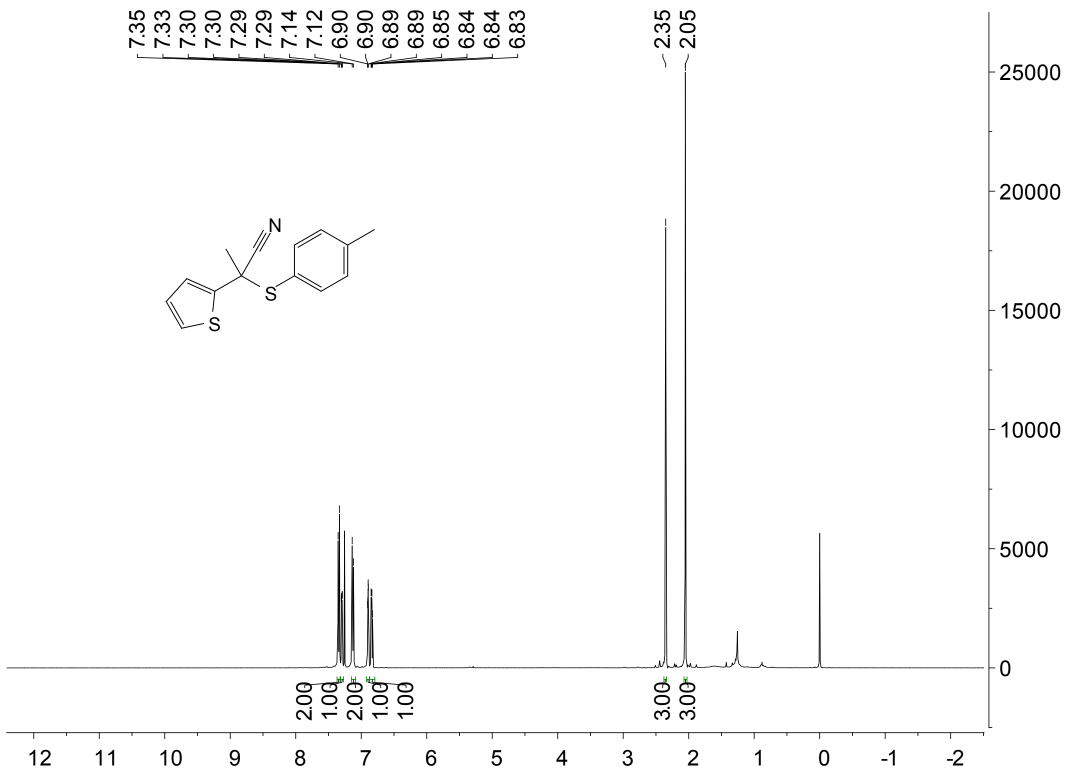

^{13}C NMR (101 MHz, $CDCl_3$) spectrum of compound 3pa

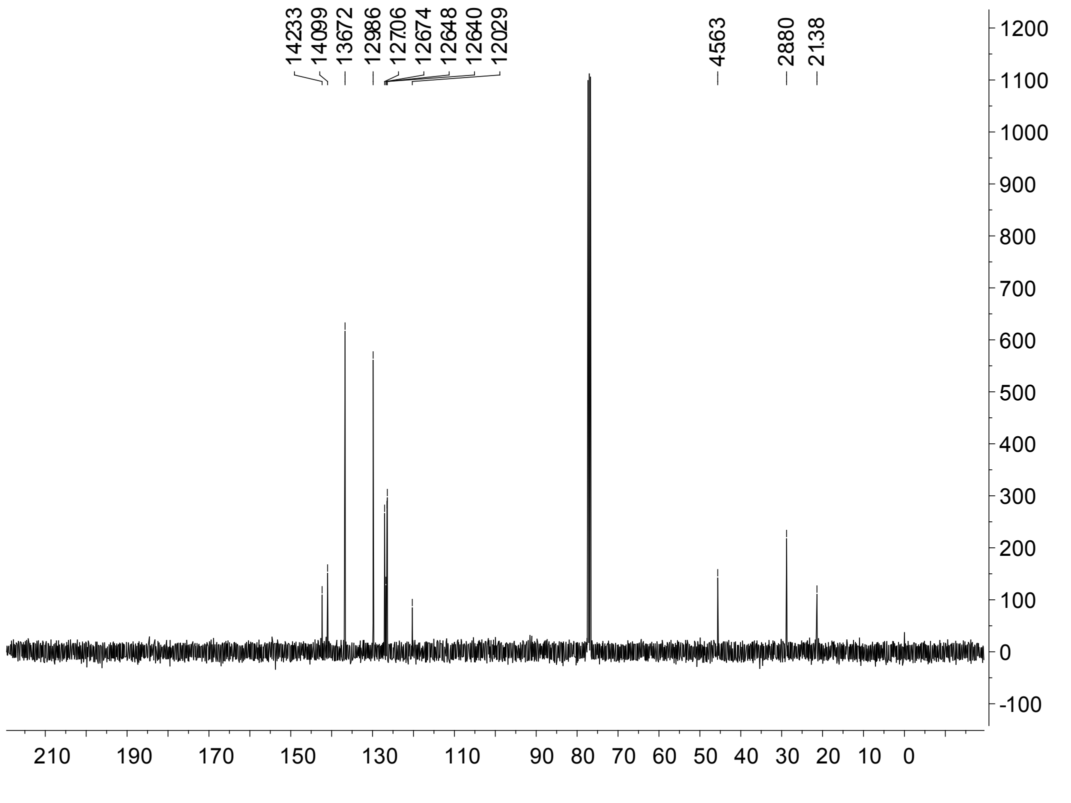

^{1}H NMR (400 MHz, $CDCl_3$) spectrum of compound 3qa

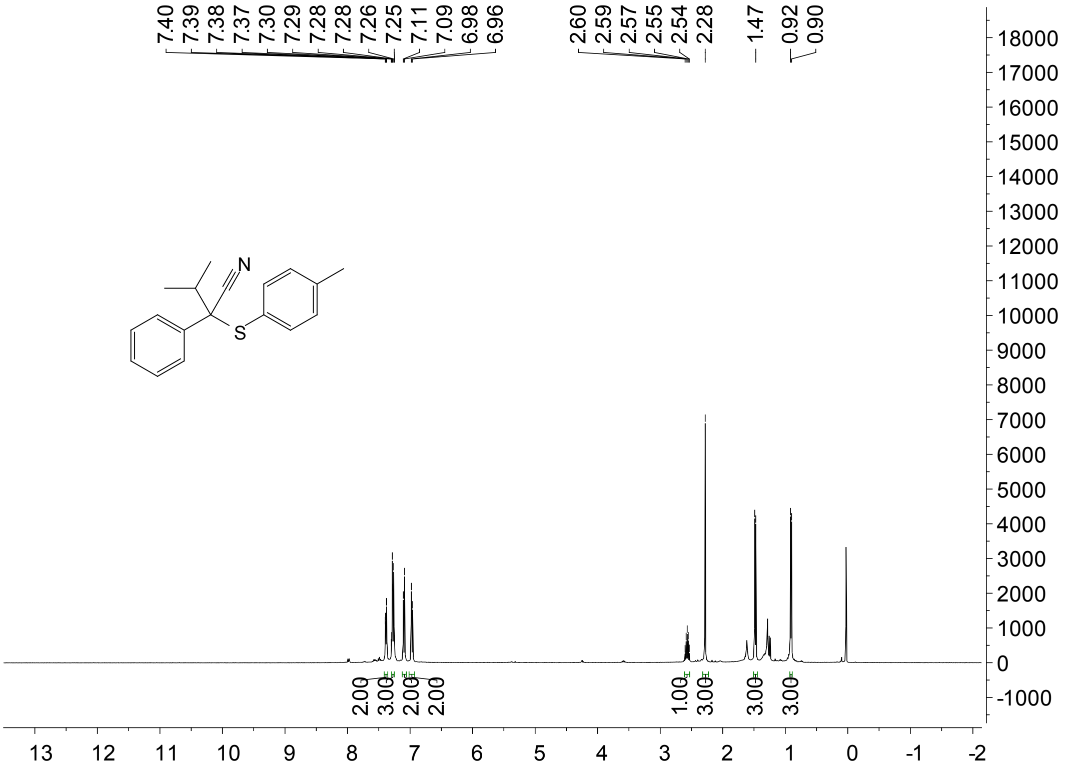

^{13}C NMR (101 MHz, $CDCl_3$) spectrum of compound 3qa

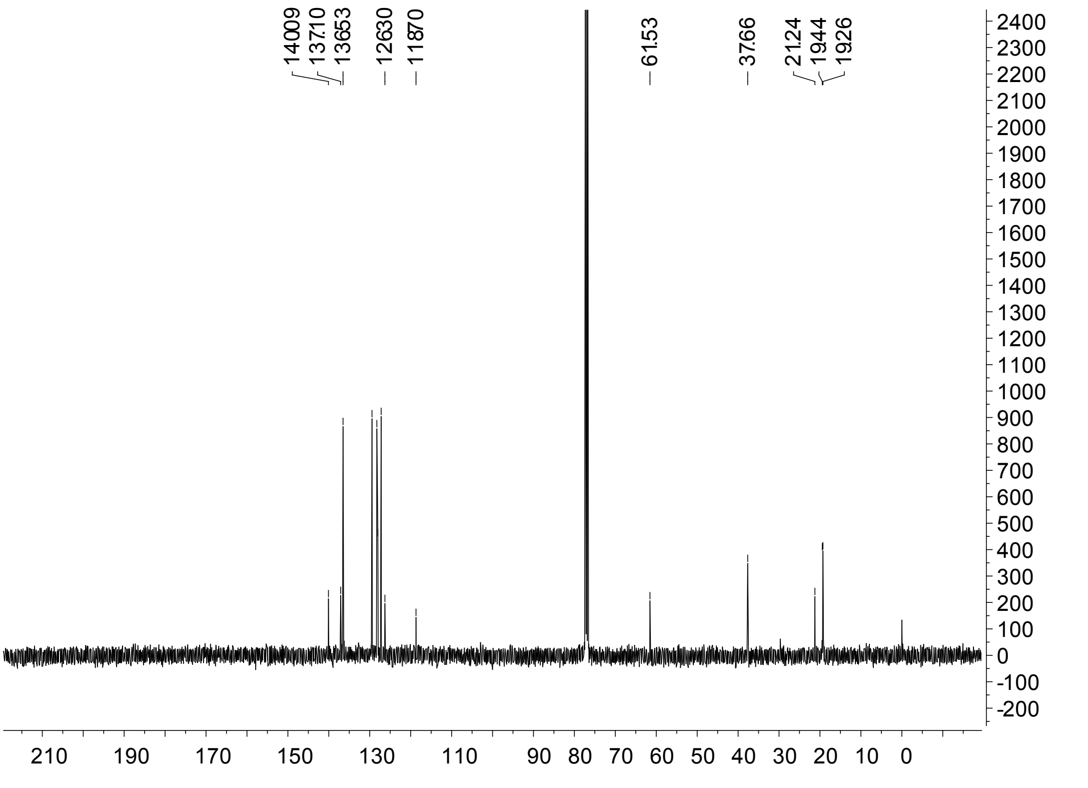

^{1}H NMR (400 MHz, $CDCl_3$) spectrum of compound 3ra

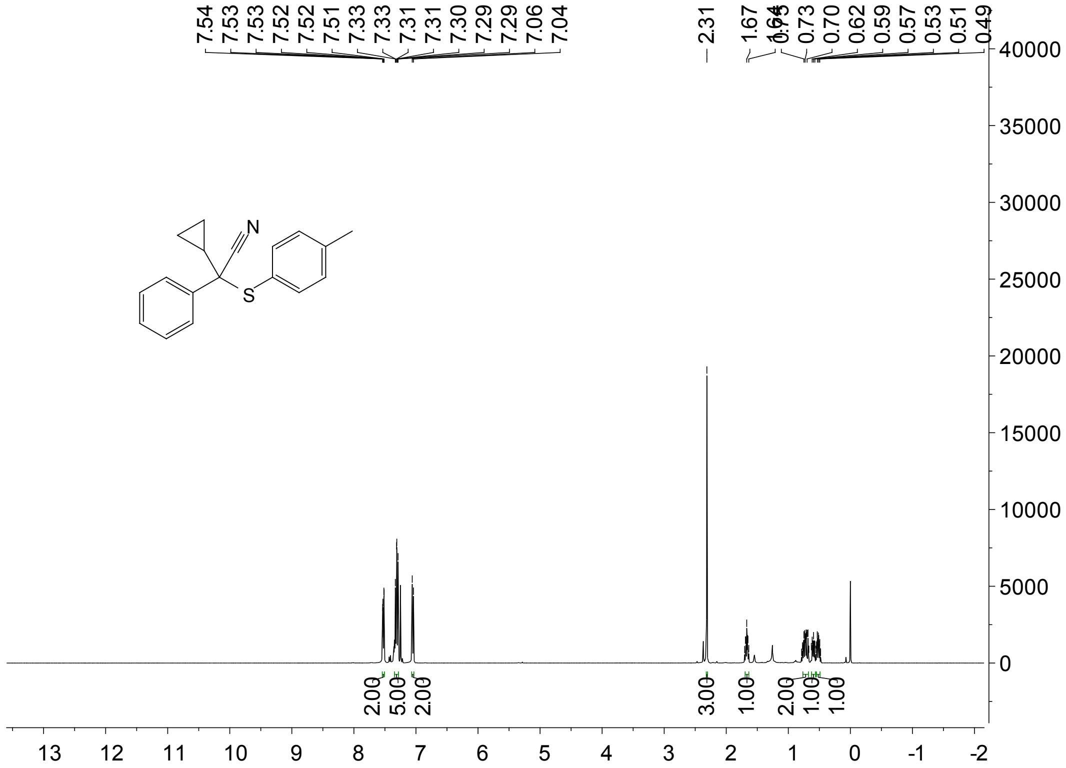

^{13}C NMR (101 MHz, $CDCl_3$) spectrum of compound 3ra

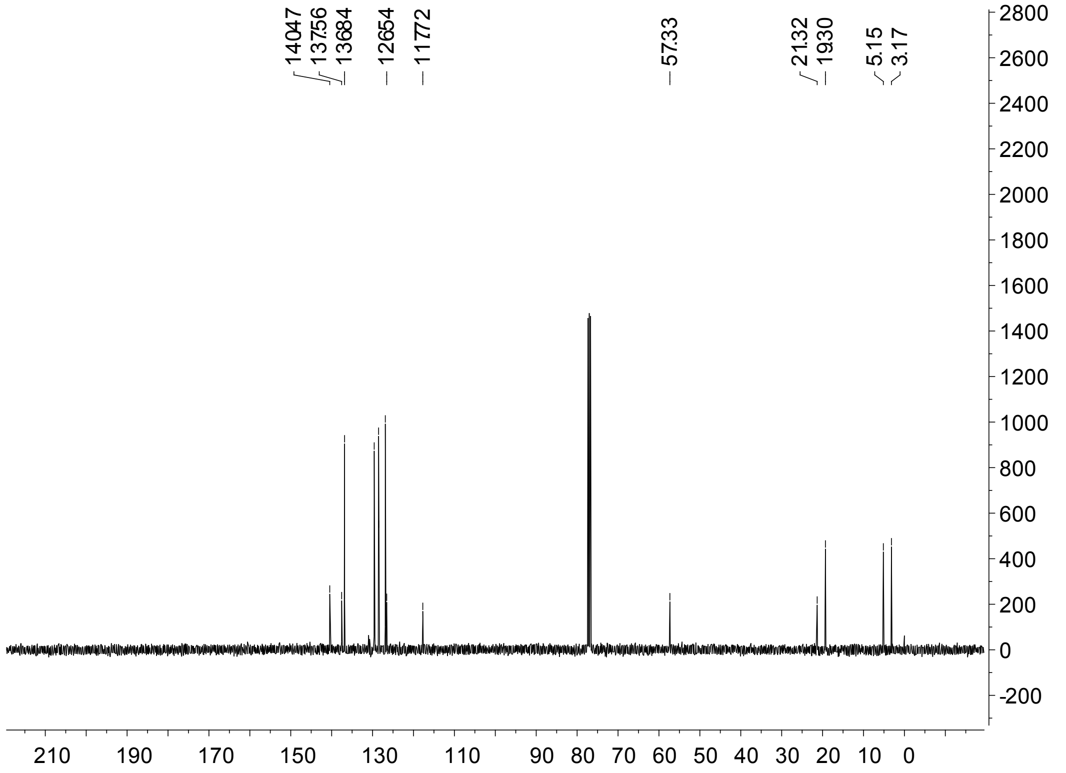

^{1}H NMR (400 MHz, $CDCl_3$) spectrum of compound 3sa

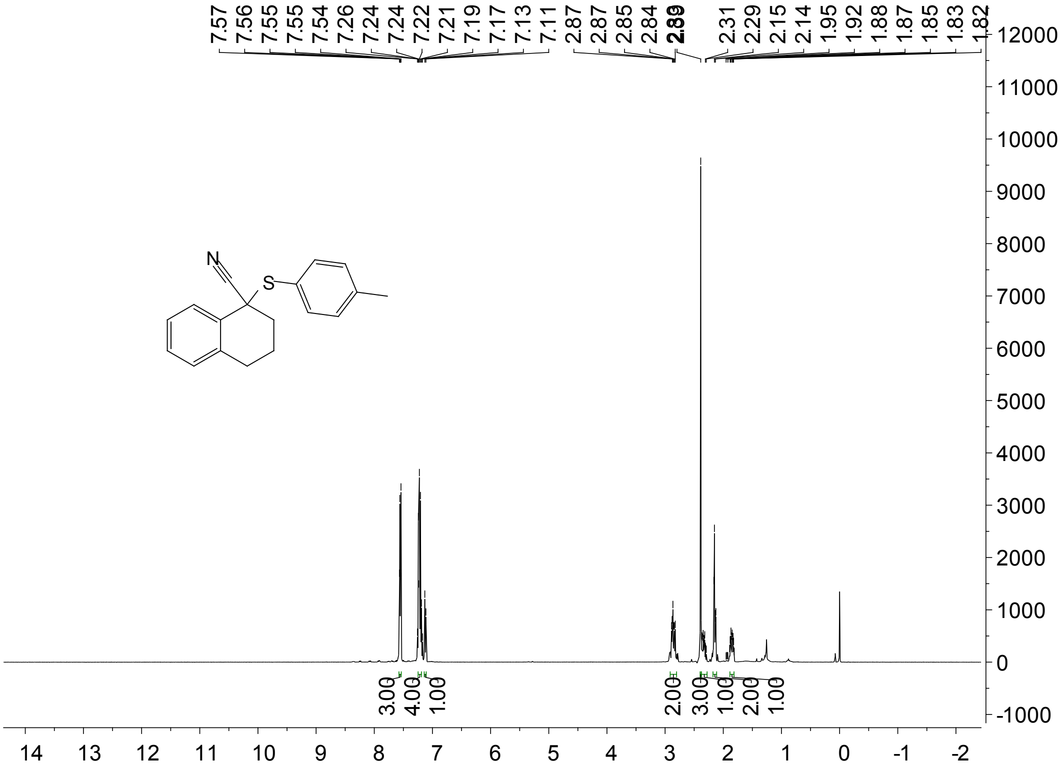

^{13}C NMR (101 MHz, $CDCl_3$) spectrum of compound 3sa

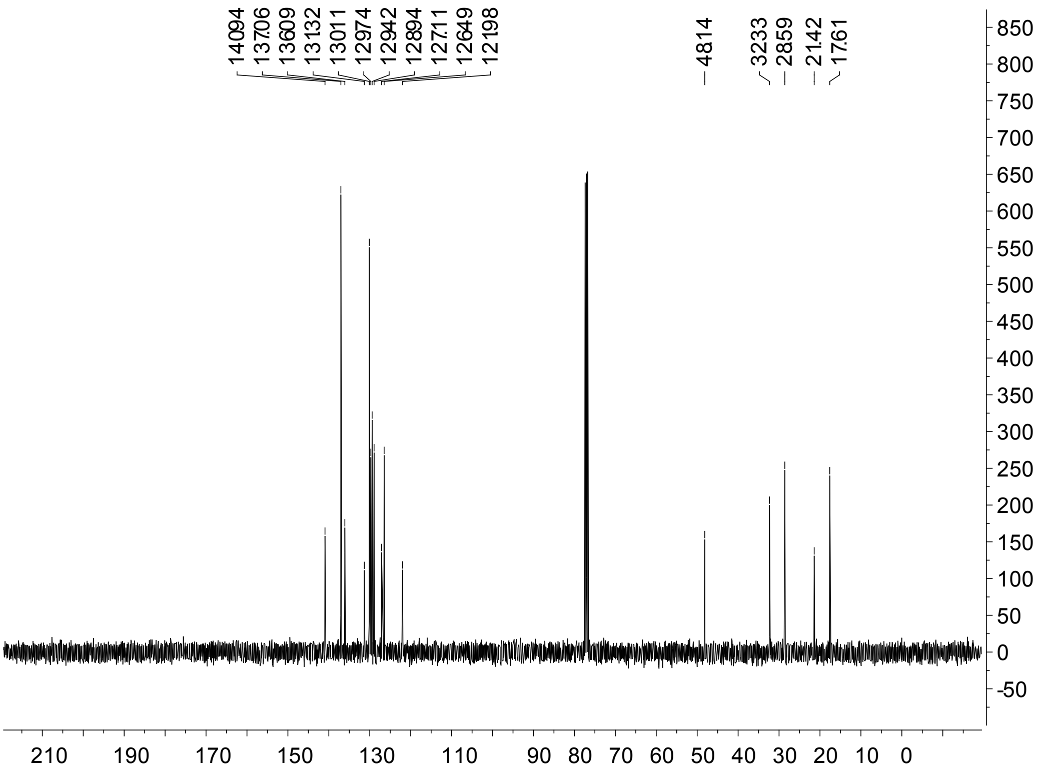

^{1}H NMR (400 MHz, $CDCl_3$) spectrum of compound 3ta

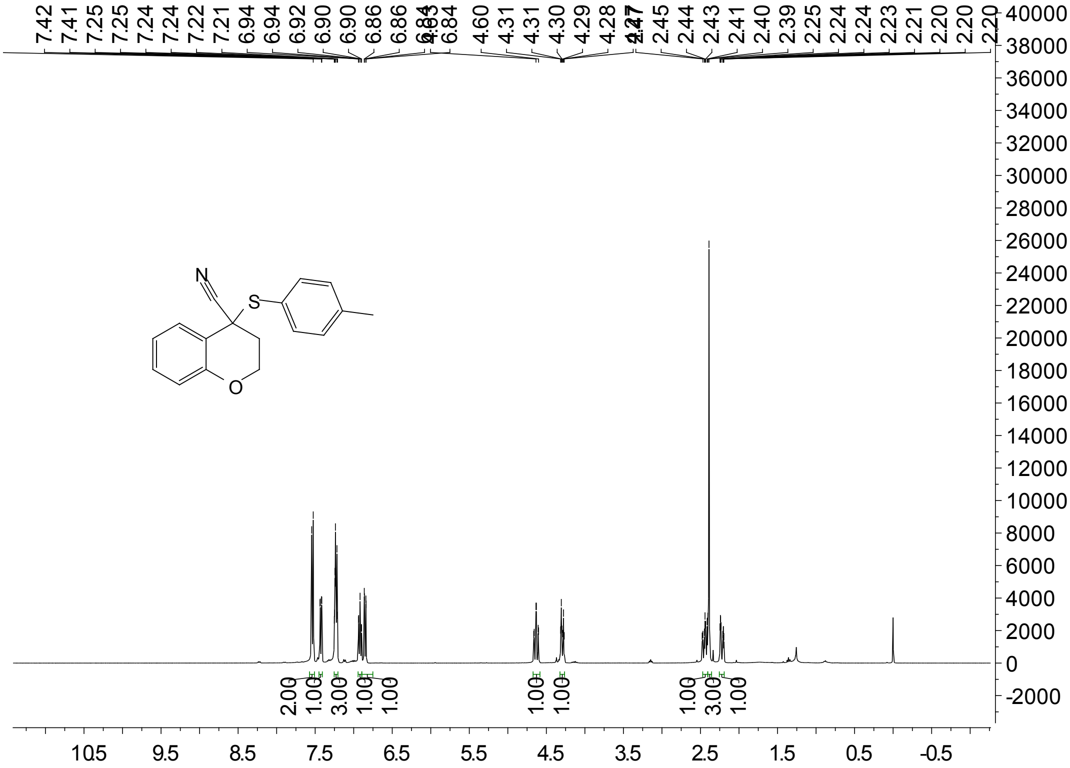

^{13}C NMR (101 MHz, $CDCl_3$) spectrum of compound 3ta

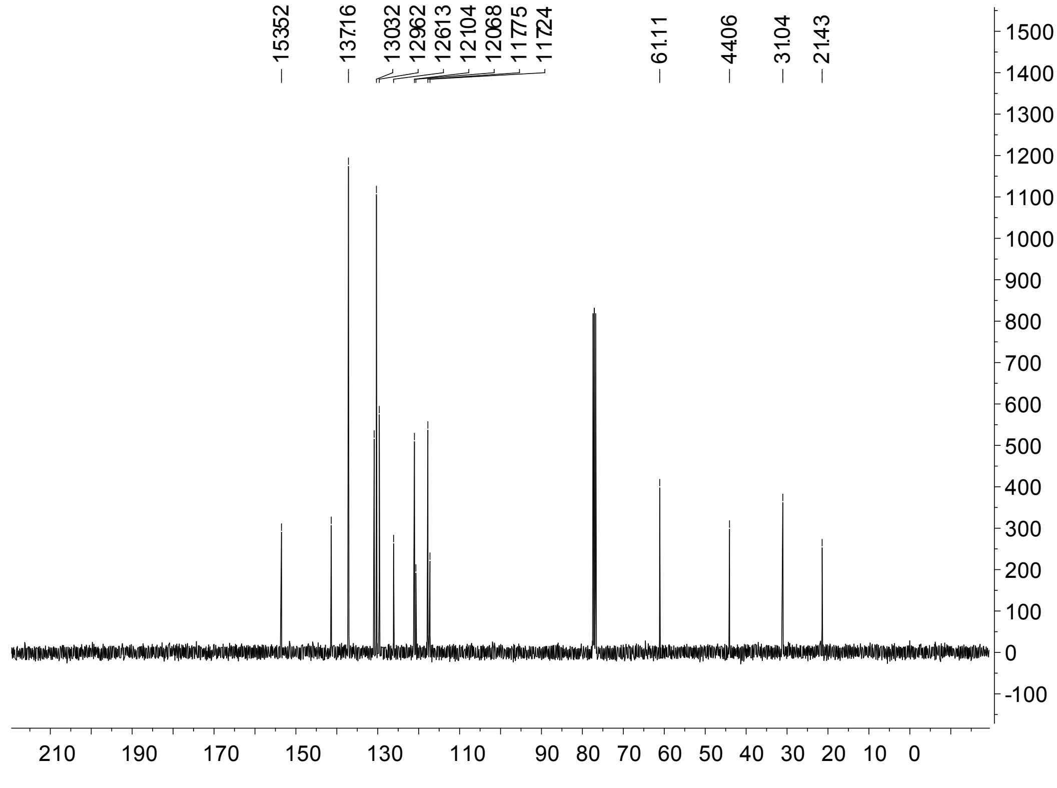

^{1}H NMR (400 MHz, $CDCl_3$) spectrum of compound 3ab

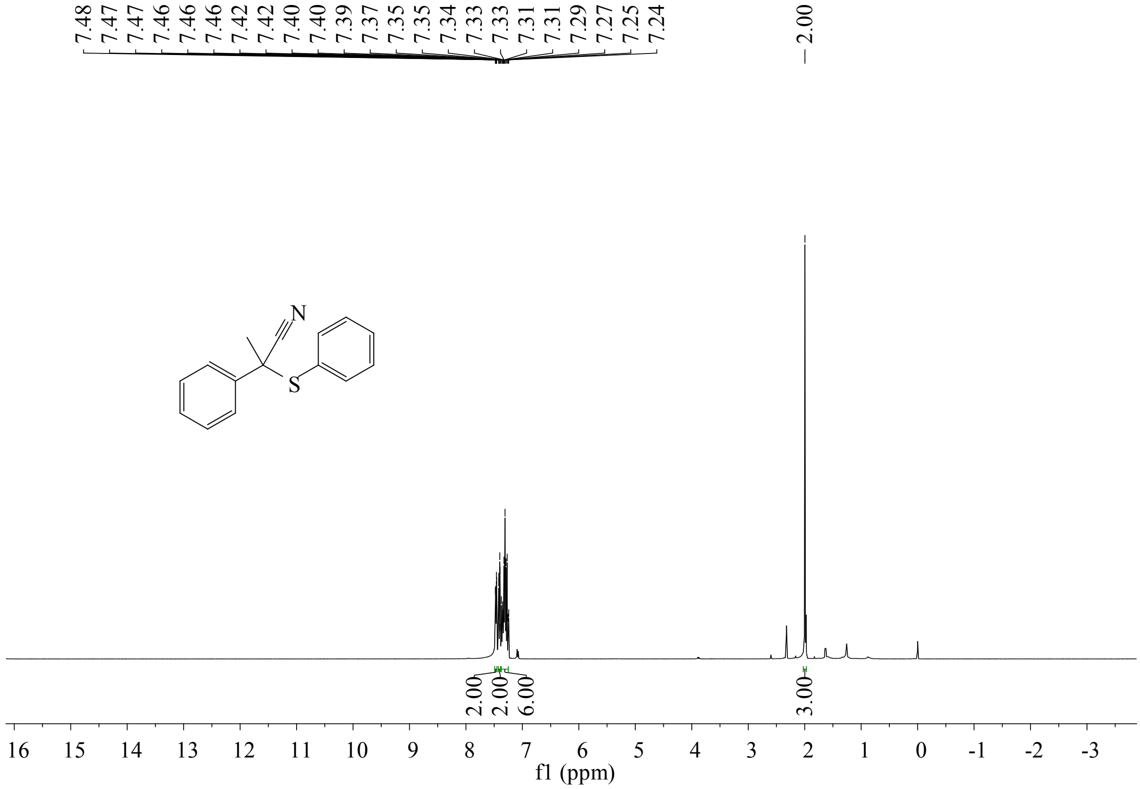

^{13}C NMR (101 MHz, $CDCl_3$) spectrum of compound 3ab

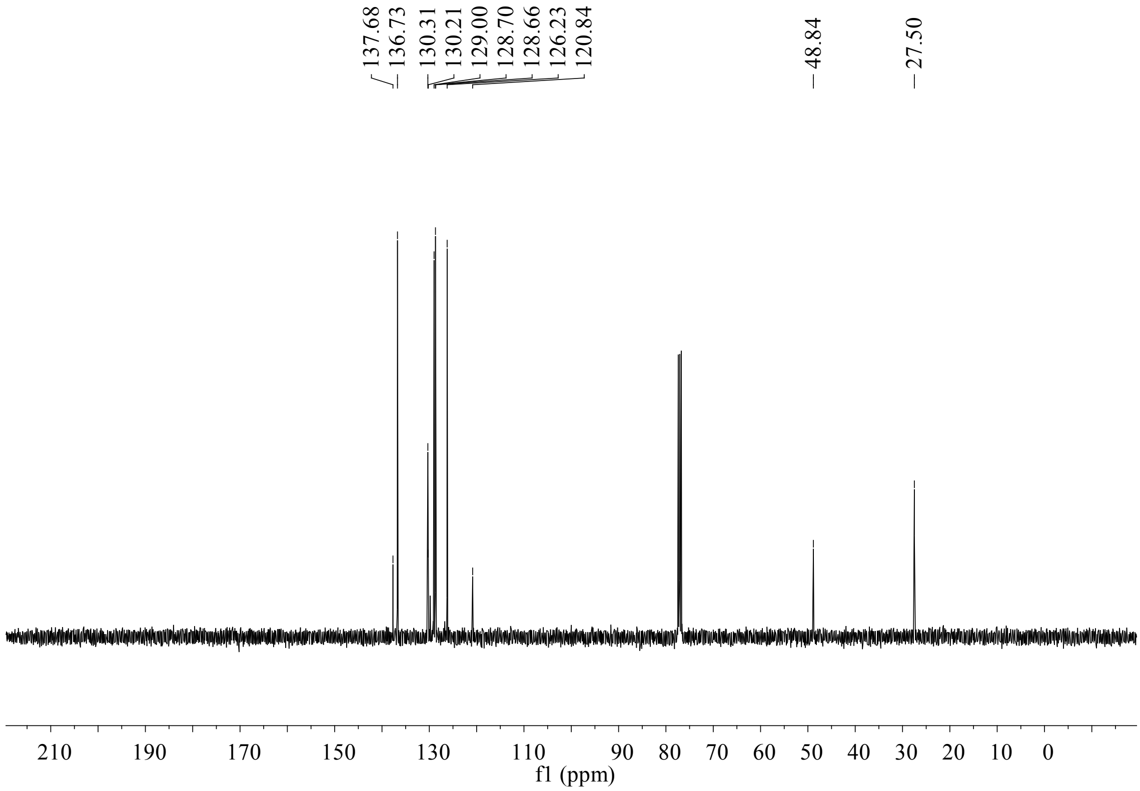

^{1}H NMR (400 MHz, $CDCl_3$) spectrum of compound 3ac

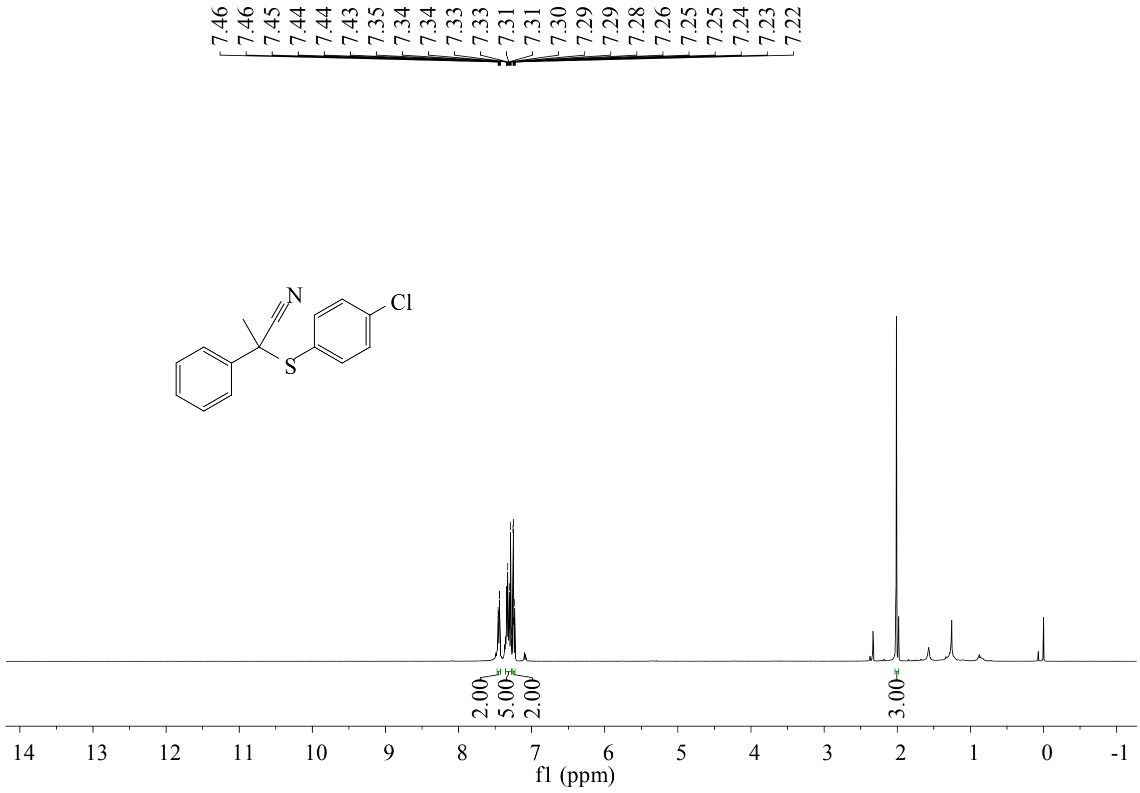

^{13}C NMR (101 MHz, $CDCl_3$) spectrum of compound 3ac

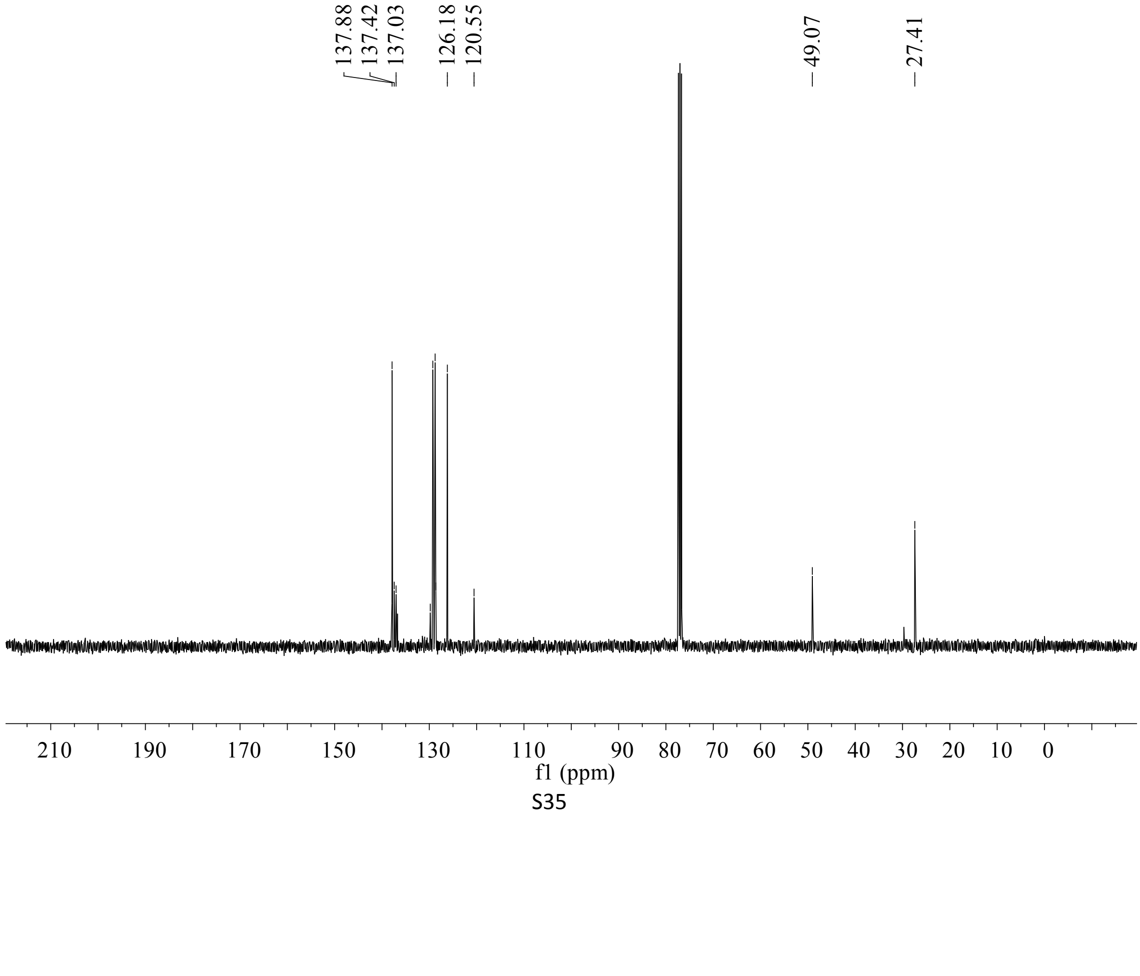

^{1}H NMR (400 MHz, $CDCl_3$) spectrum of compound 3ad

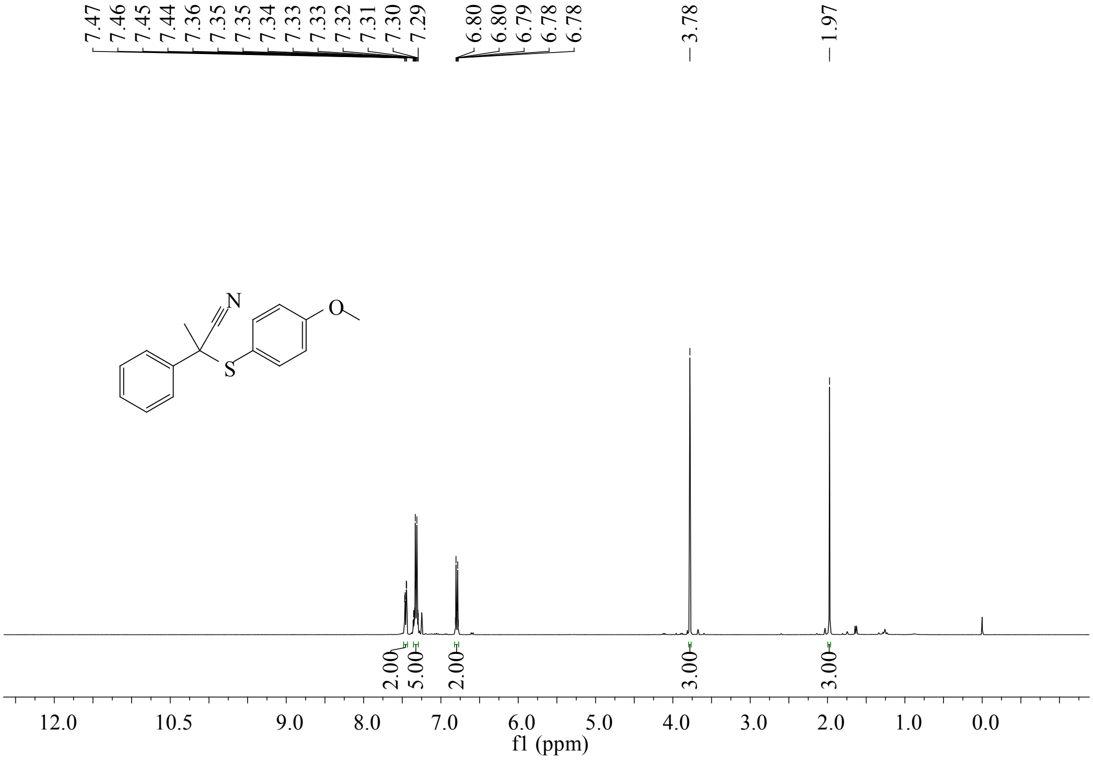

^{13}C NMR (101 MHz, $CDCl_3$) spectrum of compound 3ad

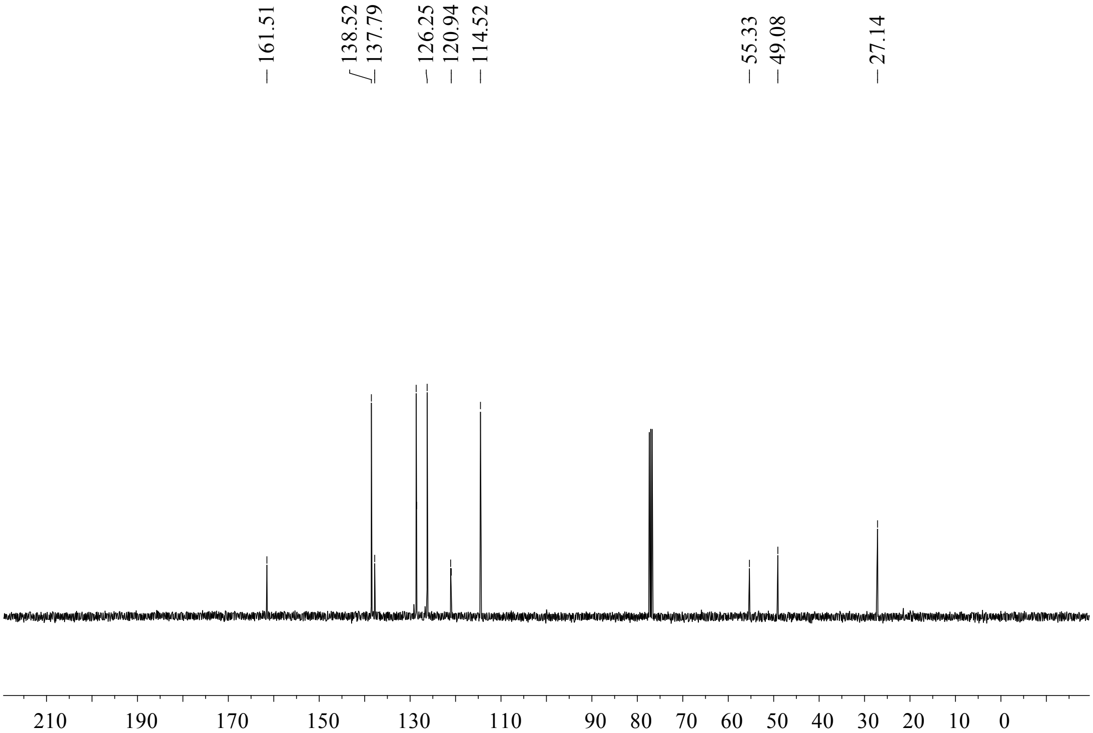

^{1}H NMR (400 MHz, $CDCl_3$) spectrum of compound 3ae

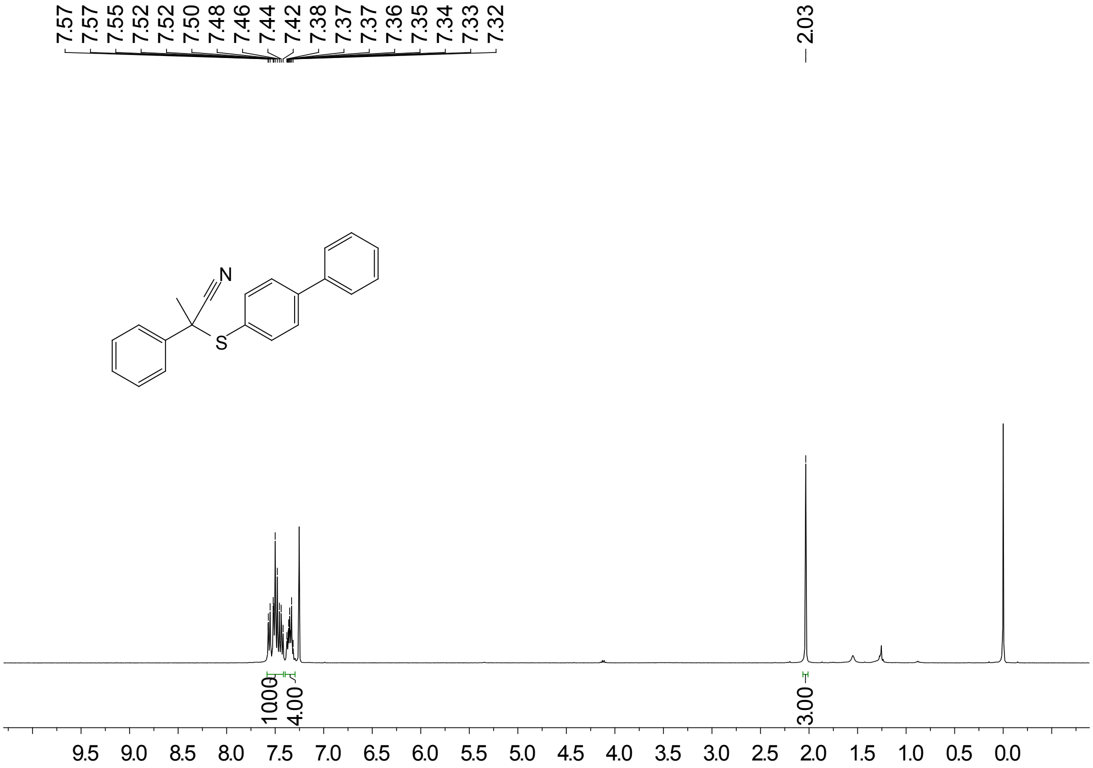

^{13}C NMR (101 MHz, $CDCl_3$) spectrum of compound 3ae

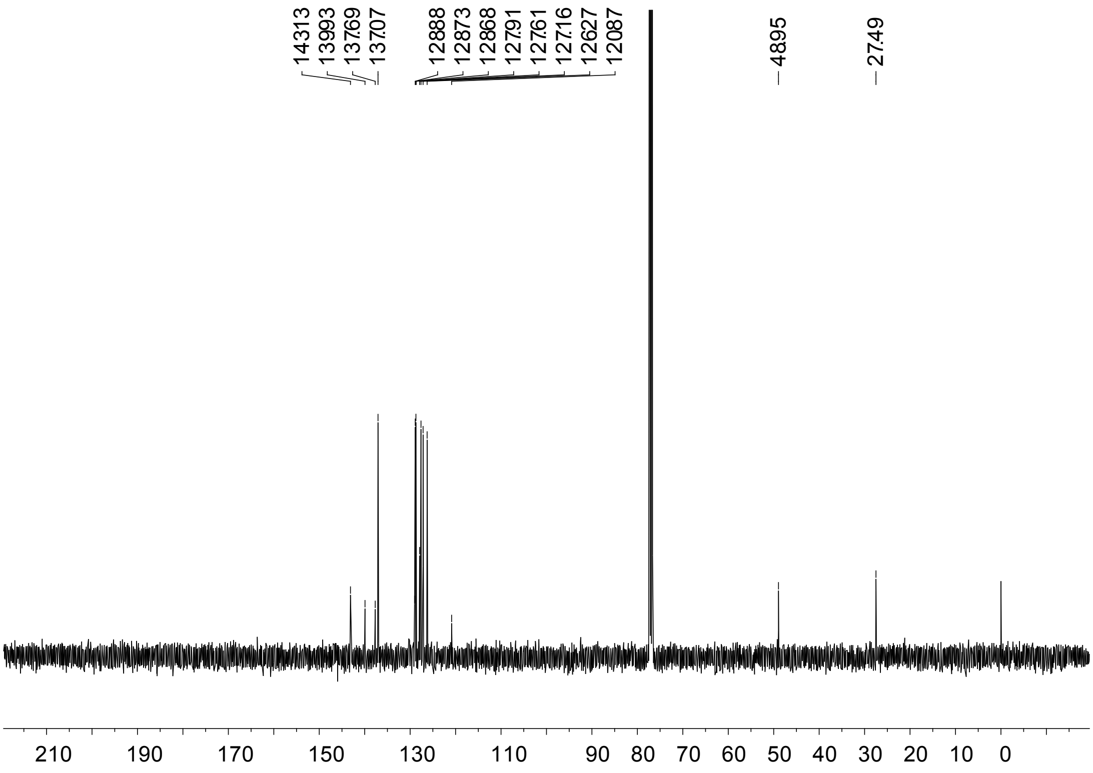

^{1}H NMR (400 MHz, $CDCl_3$) spectrum of compound 3af

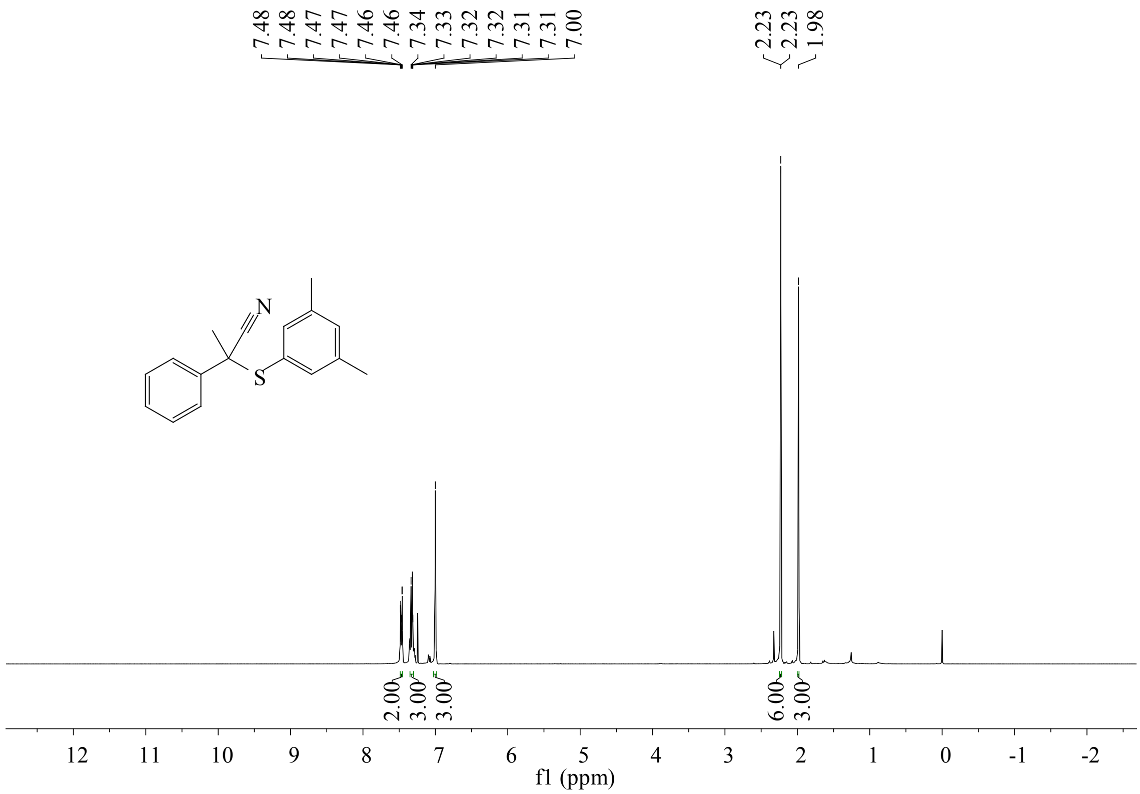

^{13}C NMR (101 MHz, $CDCl_3$) spectrum of compound 3af

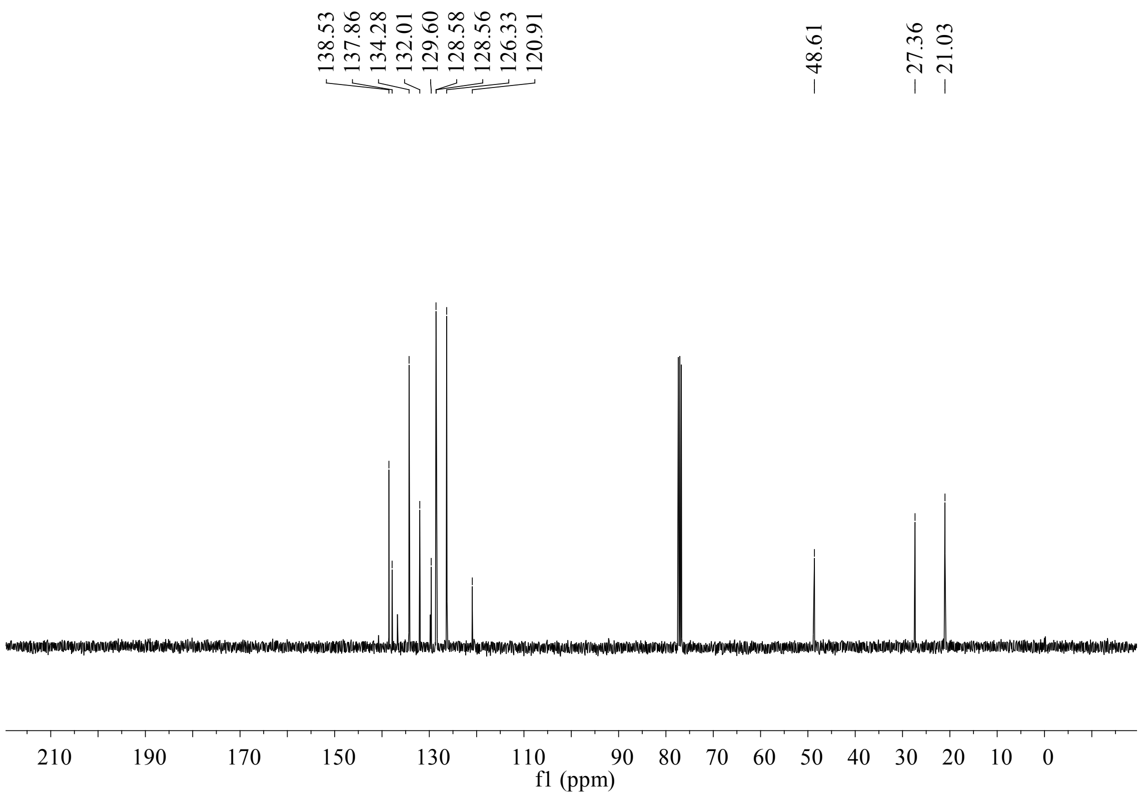

^{1}H NMR (400 MHz, $CDCl_3$) spectrum of compound 3ag

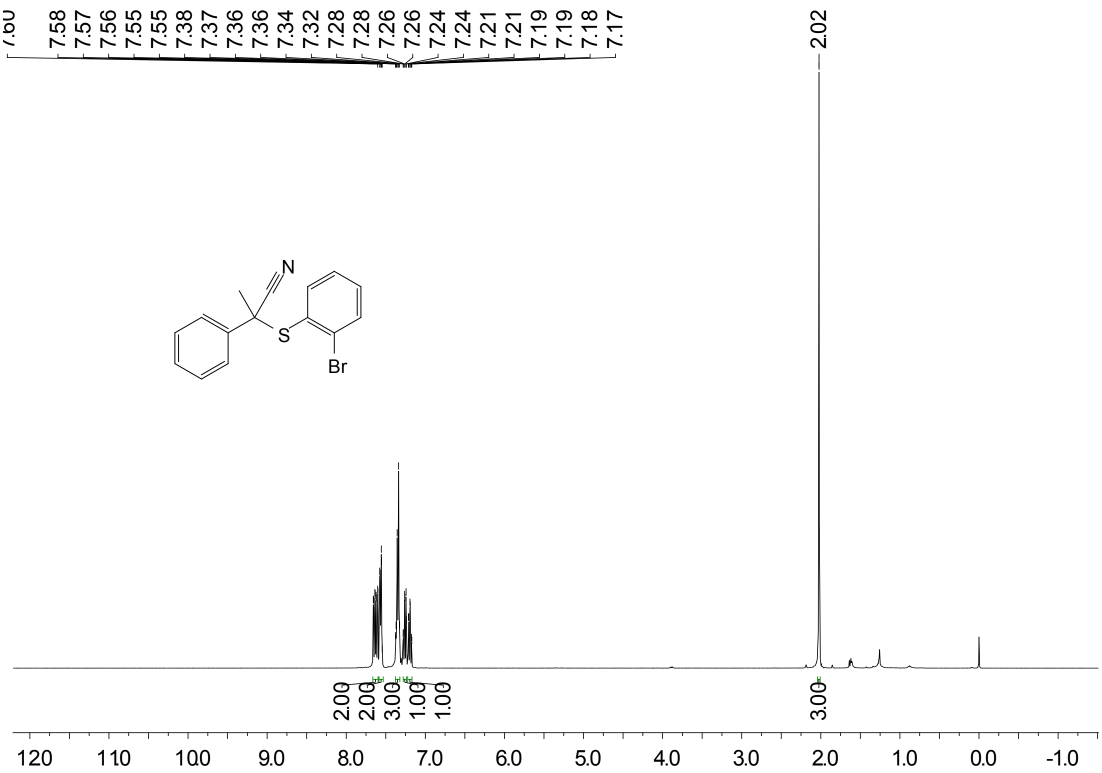

^{13}C NMR (101 MHz, $CDCl_3$) spectrum of compound 3ag

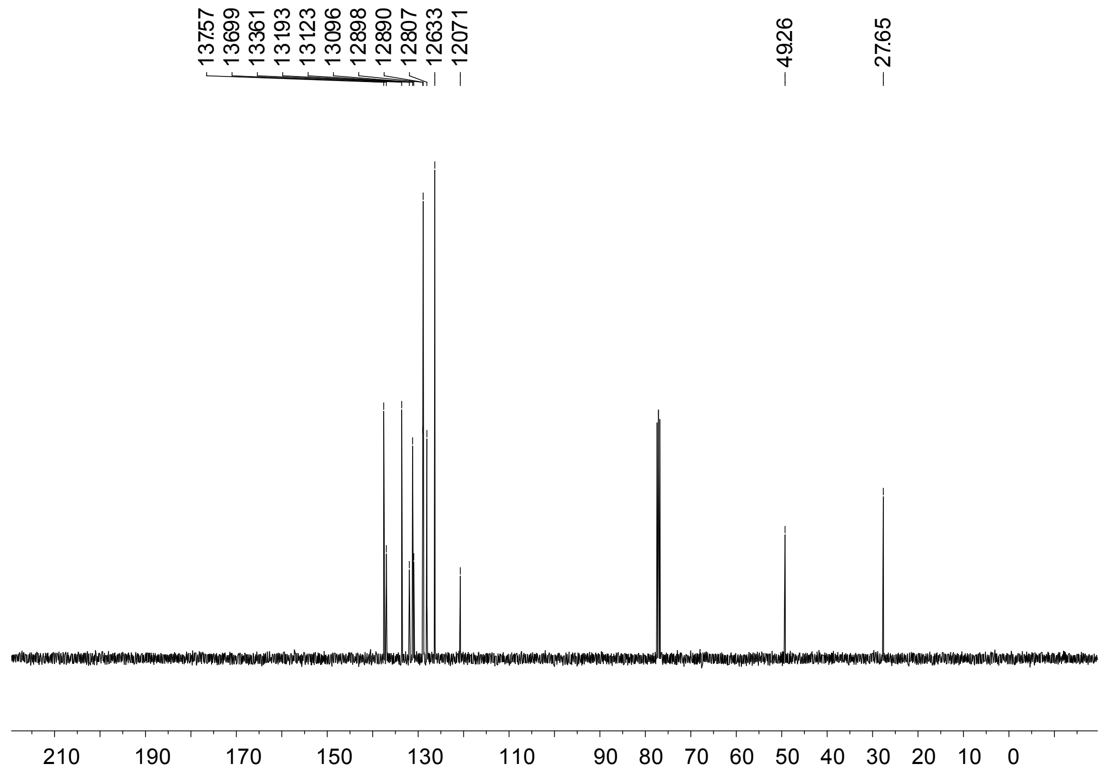

^{1}H NMR (400 MHz, $CDCl_3$) spectrum of compound 3ah

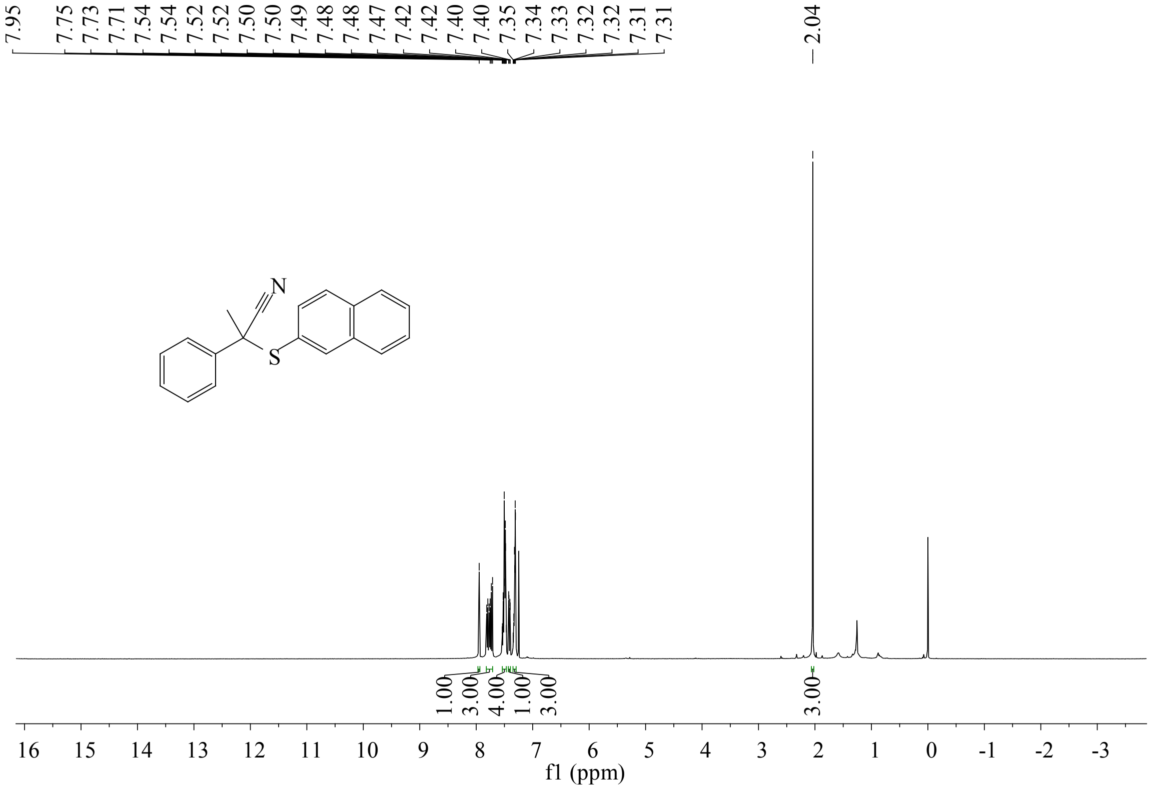

^{13}C NMR (101 MHz, $CDCl_3$) spectrum of compound 3ah

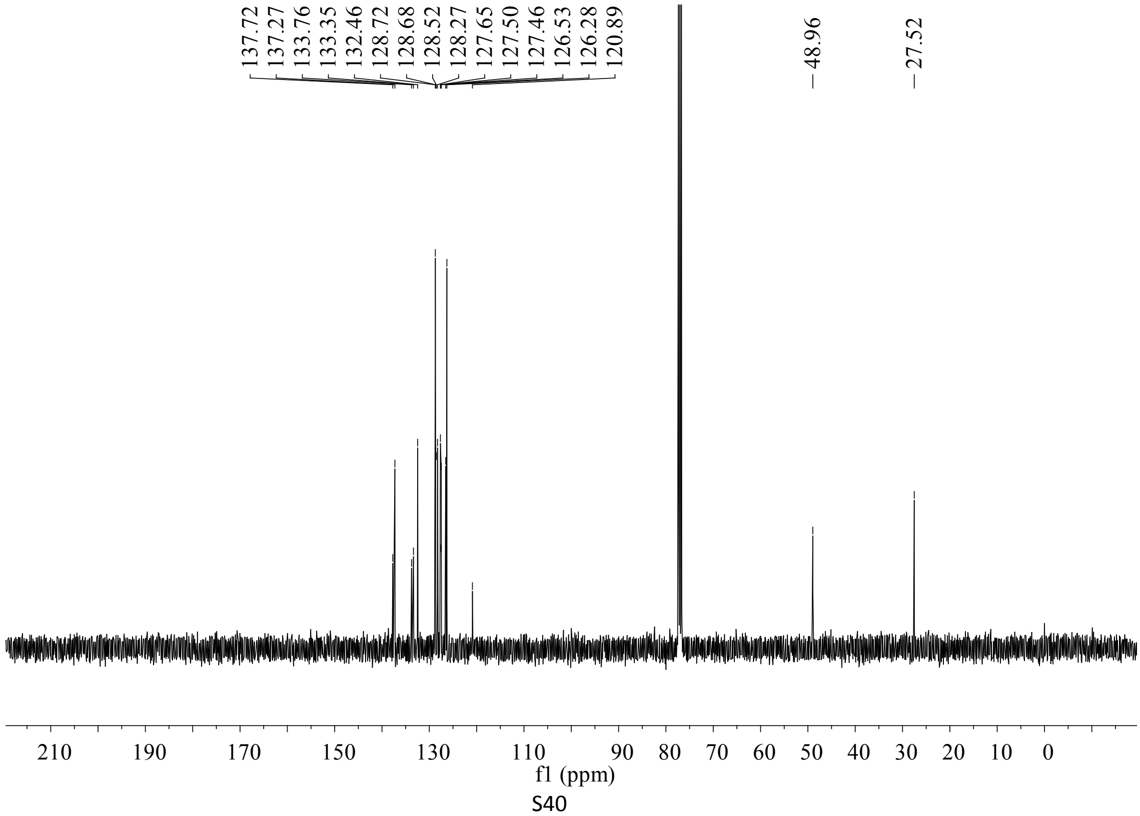

^{1}H NMR (400 MHz, CDCl$_3$) spectrum of compound 3ai

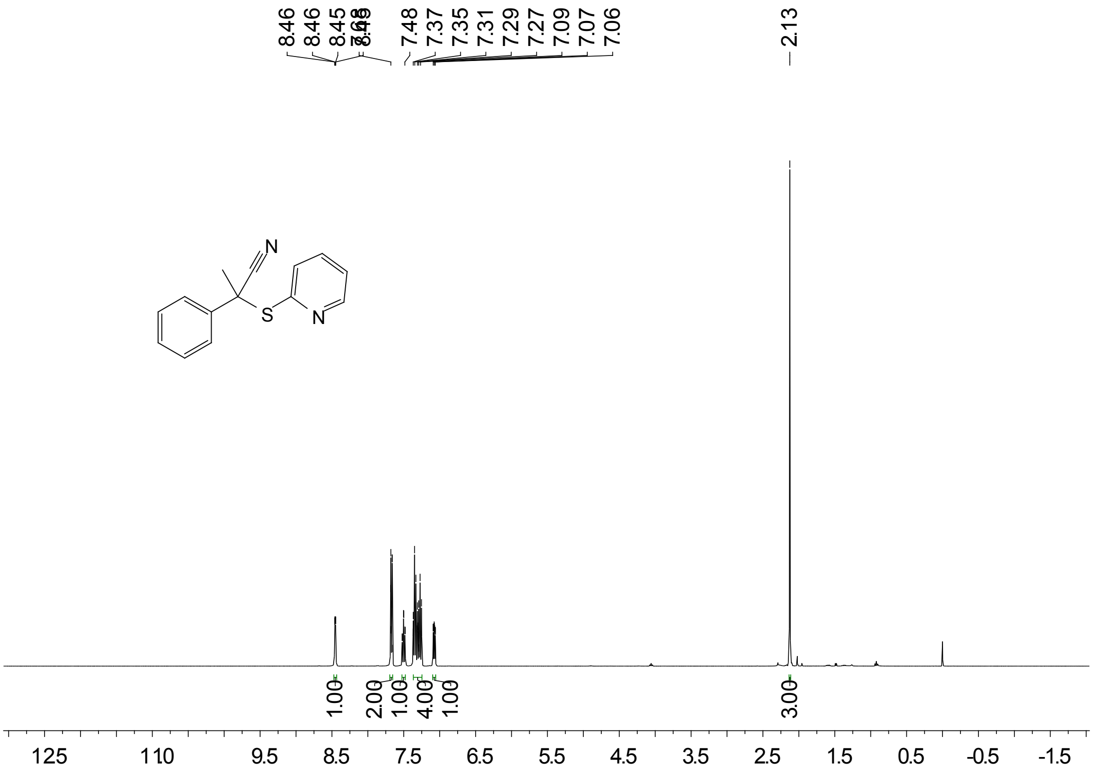

^{13}C NMR (101 MHz, CDCl$_3$) spectrum of compound 3ai

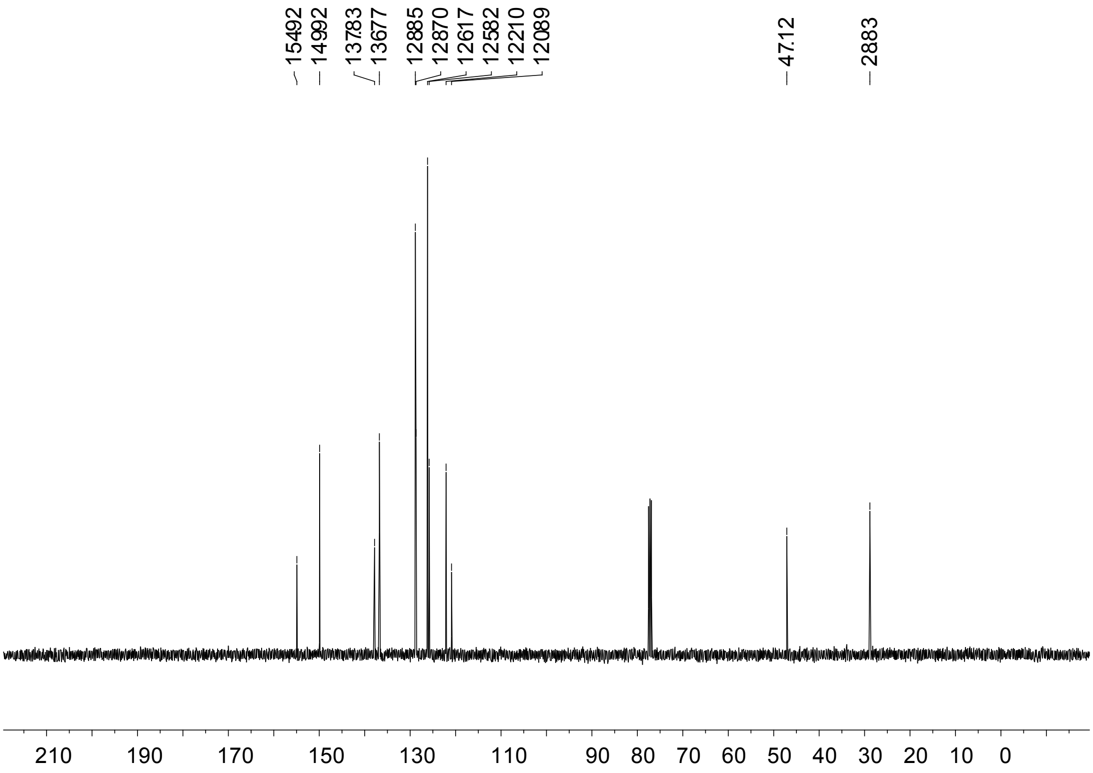

^{1}H NMR (400 MHz, CDCl$_3$) spectrum of compound 3aj

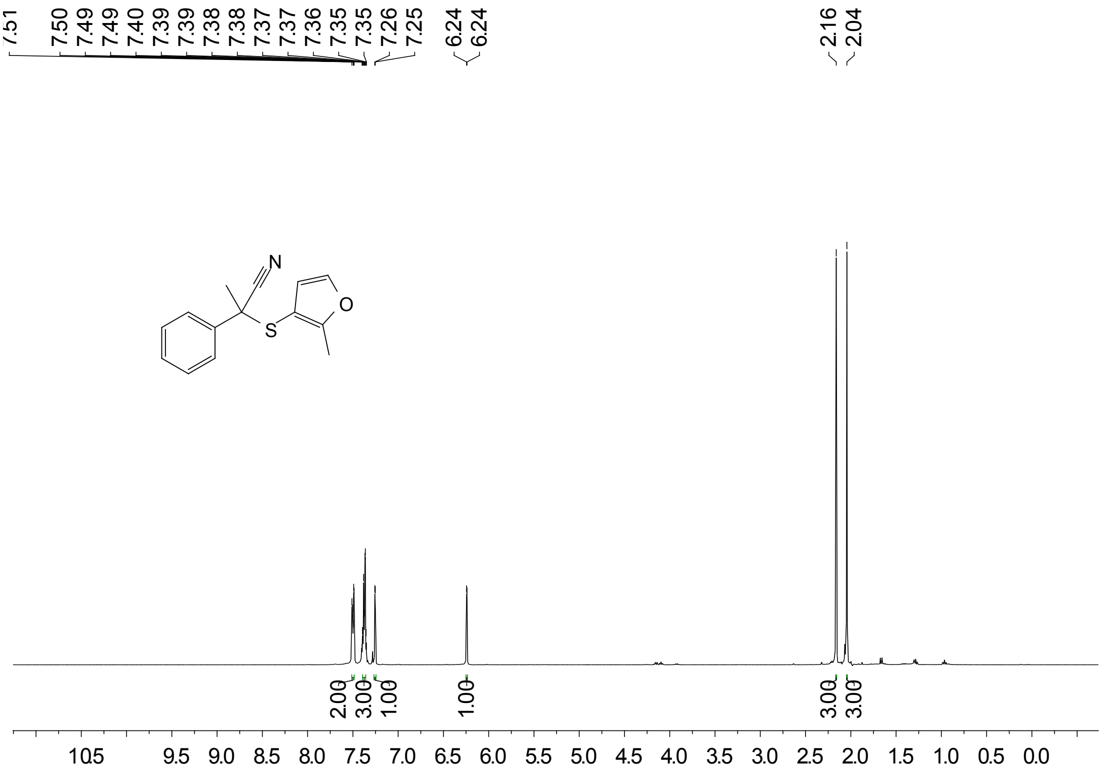

^{13}C NMR (101 MHz, CDCl$_3$) spectrum of compound 3aj

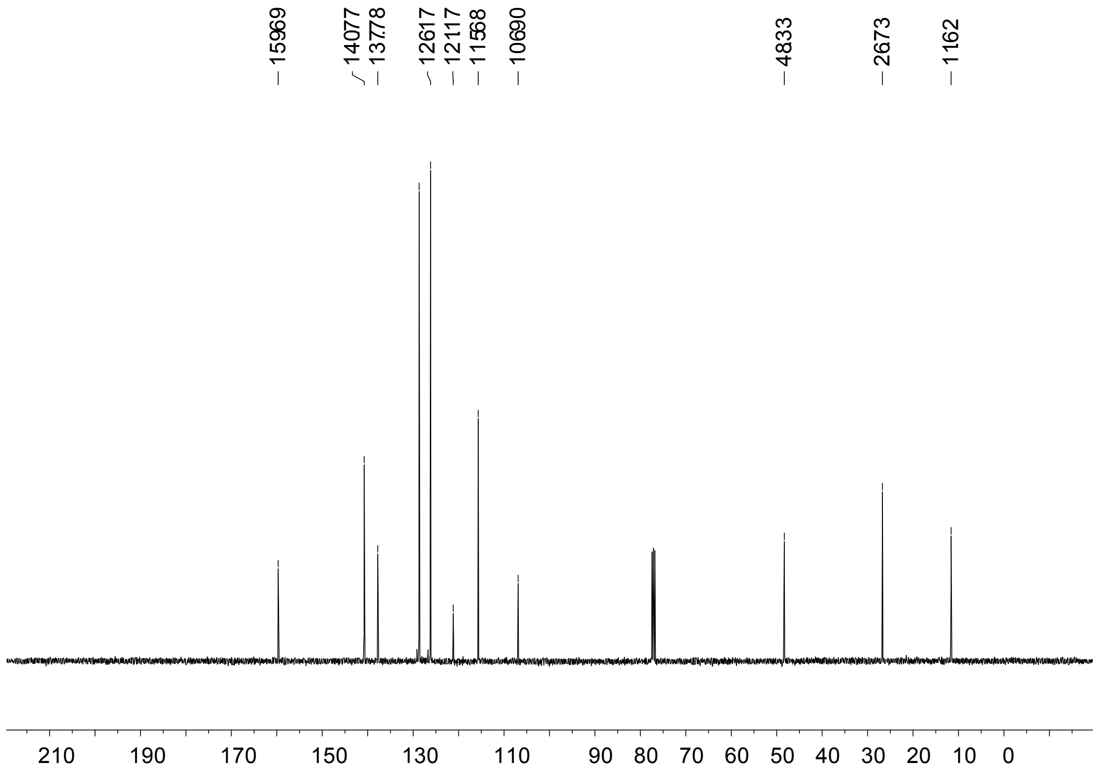

^{1}H NMR (400 MHz, CDCl$_3$) spectrum of compound 3ak

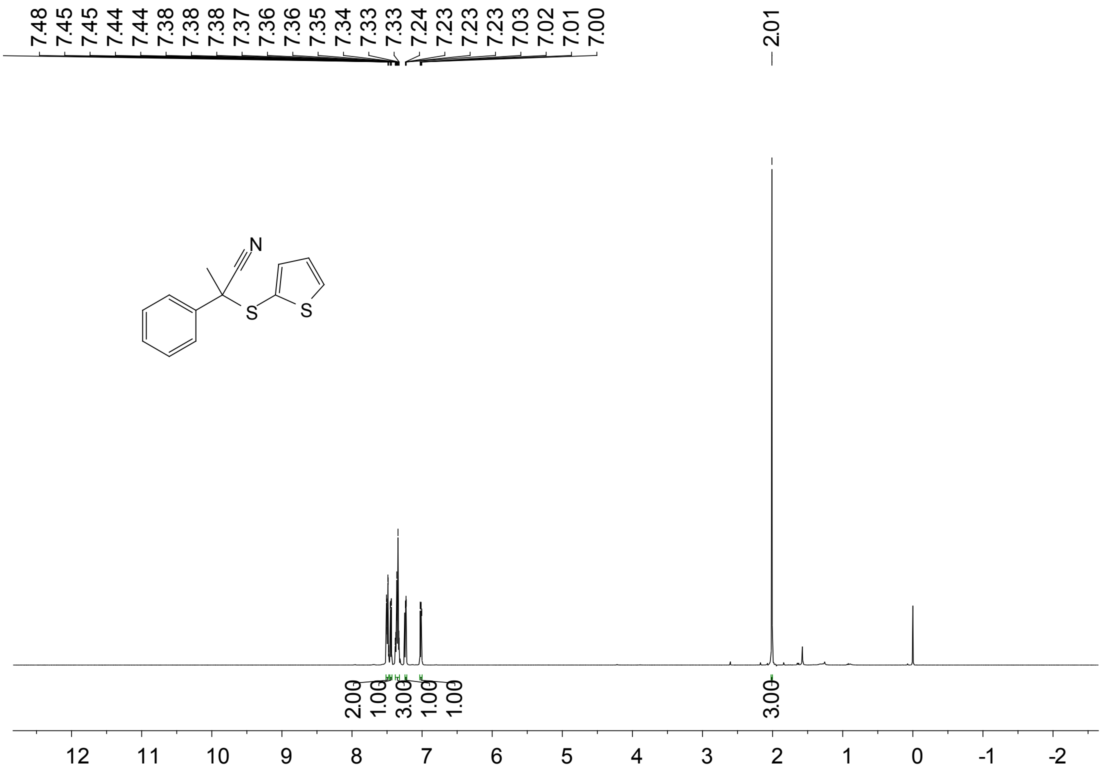

^{13}C NMR (101 MHz, CDCl$_3$) spectrum of compound 3ak

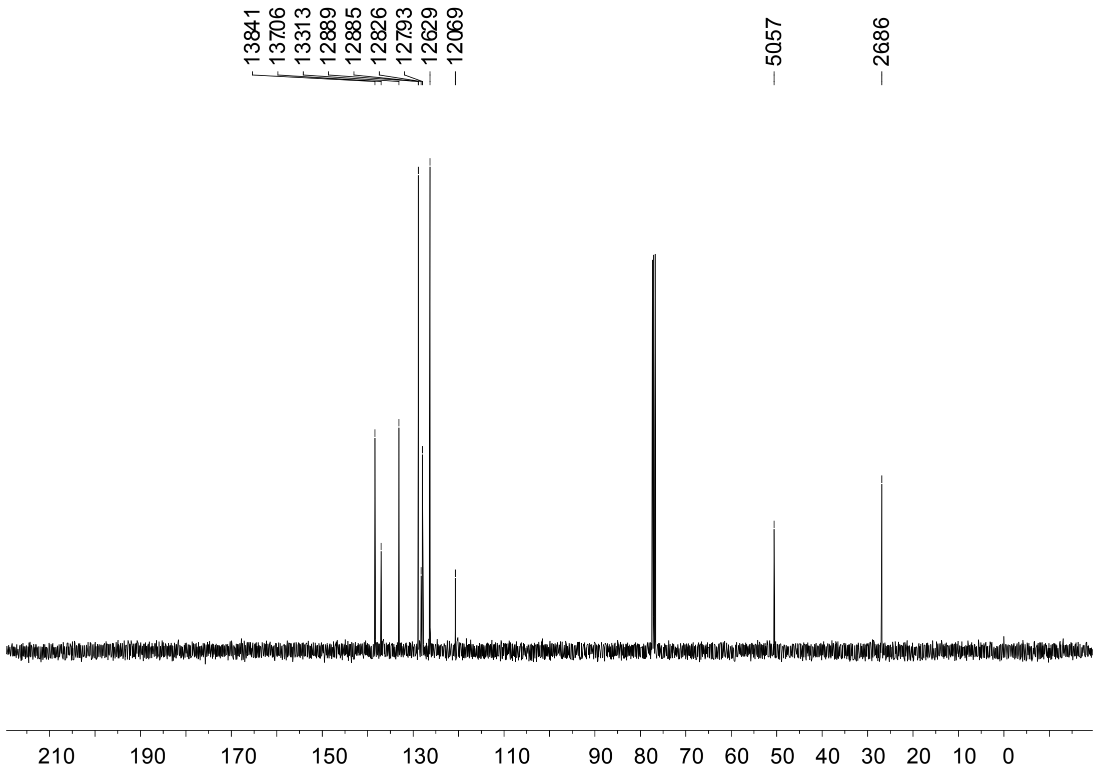

^{1}H NMR (400 MHz, $CDCl_3$) spectrum of compound 3al

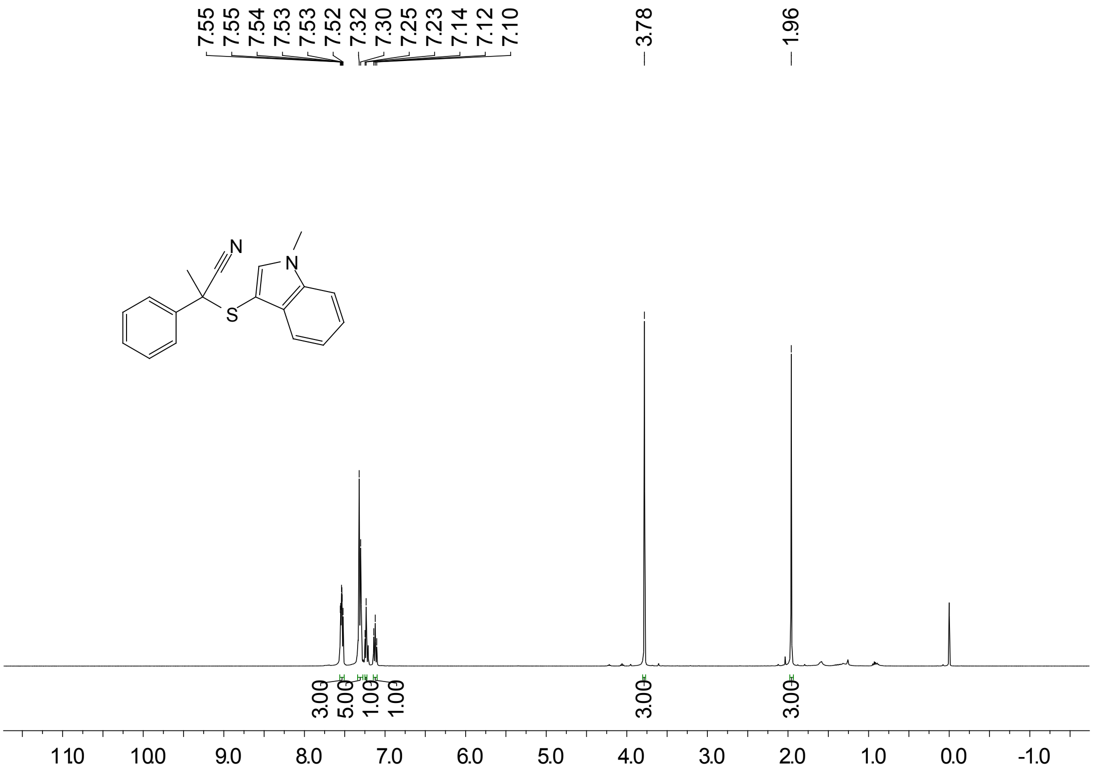

^{13}C NMR (101 MHz, $CDCl_3$) spectrum of compound 3al

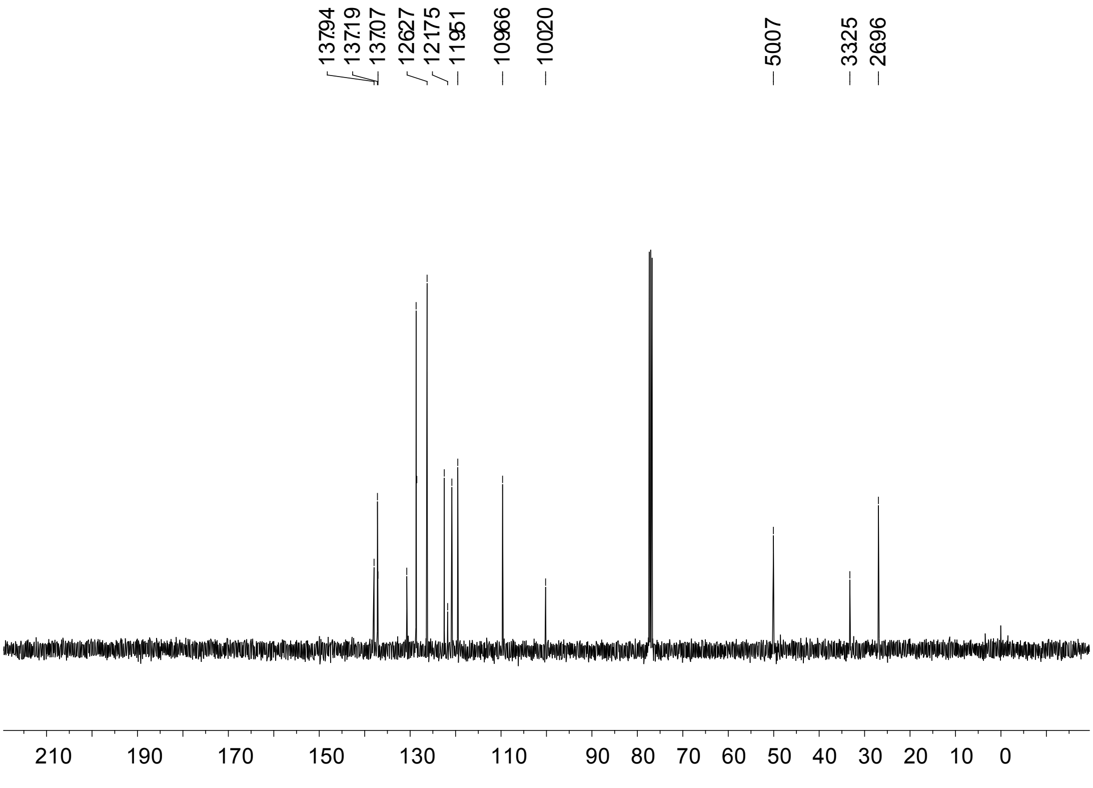

^{1}H NMR (400 MHz, $CDCl_3$) spectrum of compound 3am

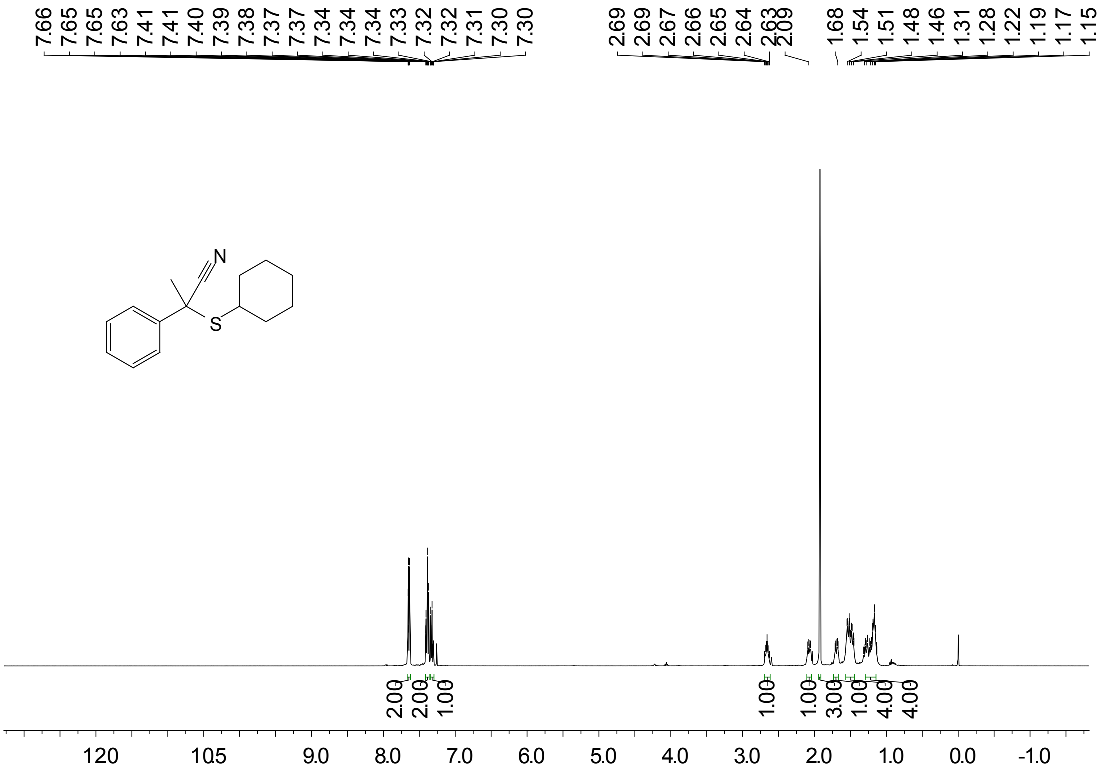

^{13}C NMR (101 MHz, $CDCl_3$) spectrum of compound 3am

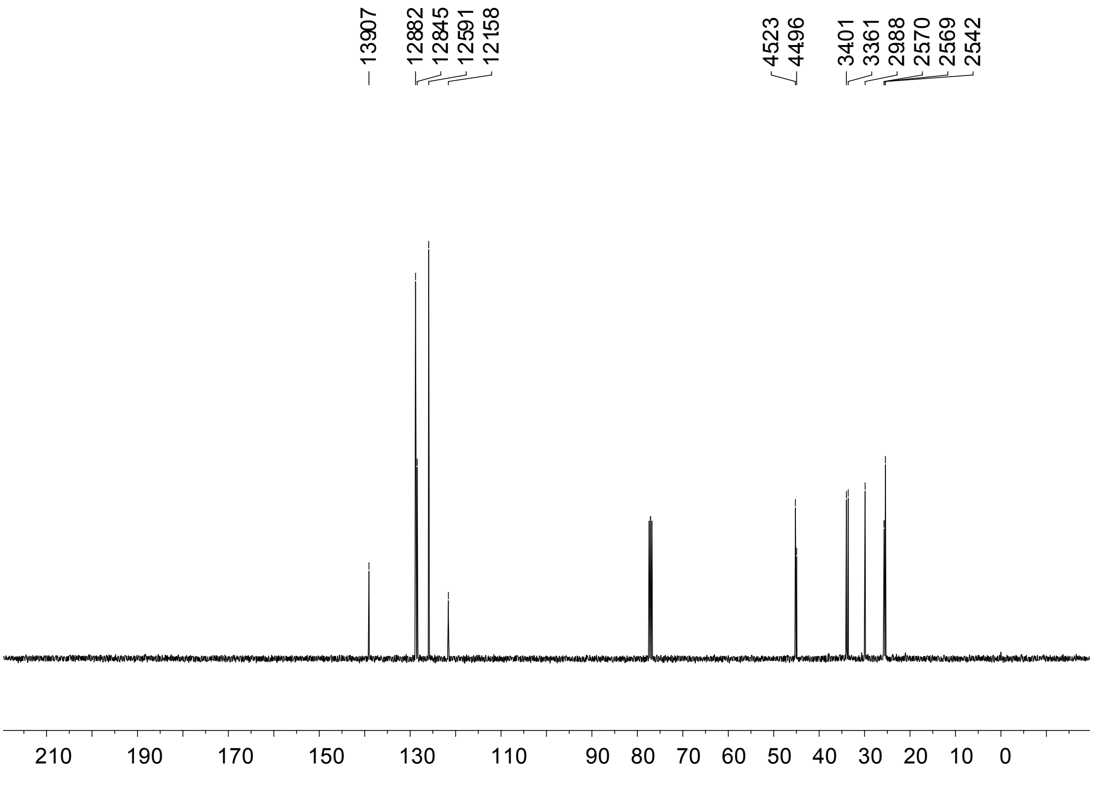

^{1}H NMR (400 MHz, $CDCl_3$) spectrum of compound 3ua

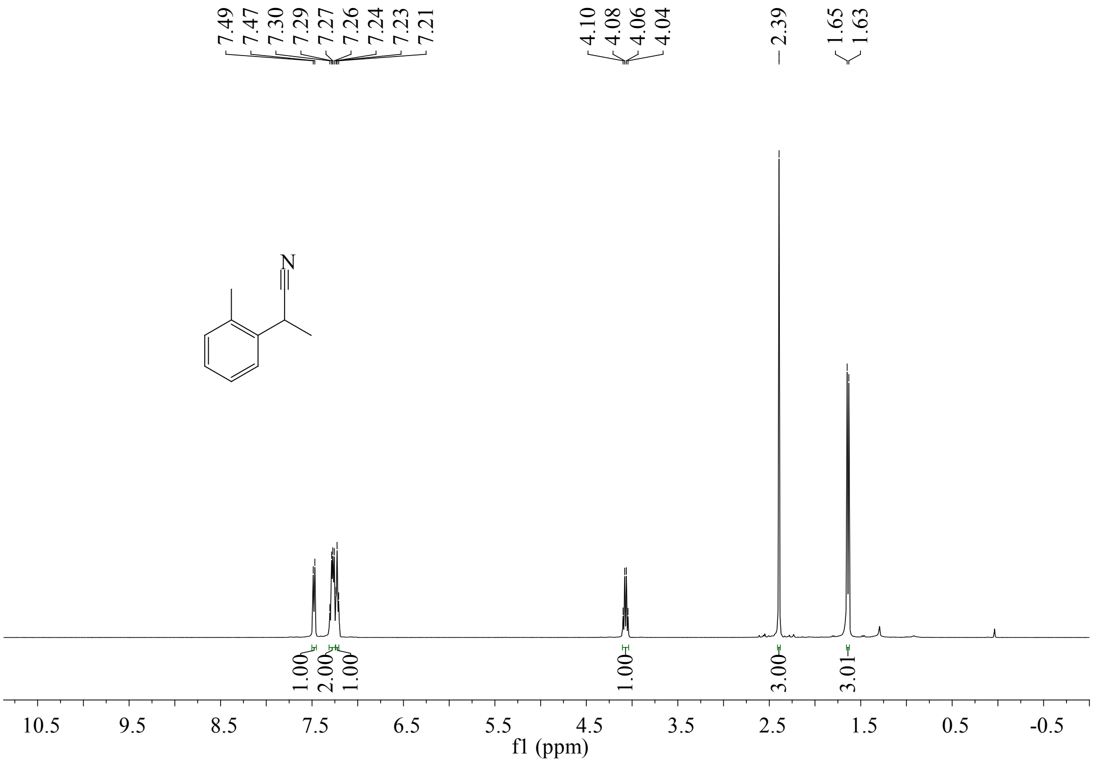

^{13}C NMR (101 MHz, $CDCl_3$) spectrum of compound 3ua

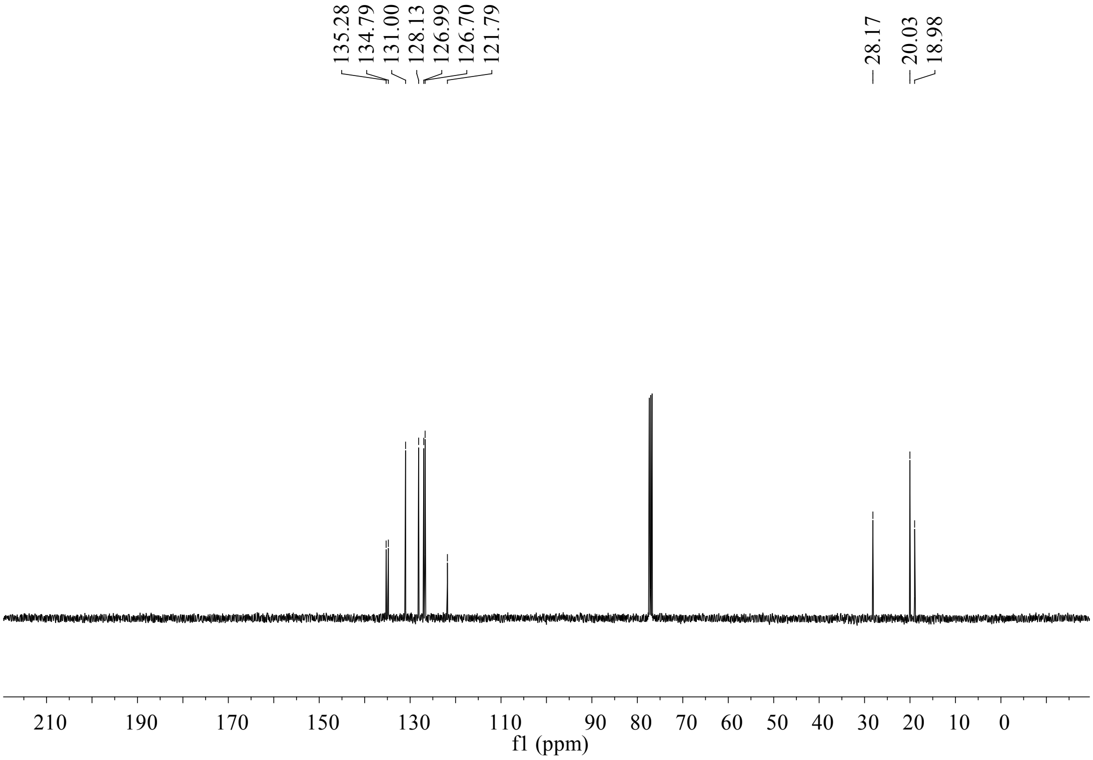

^{1}H NMR (400 MHz, CDCl$_3$) spectrum of compound 3va

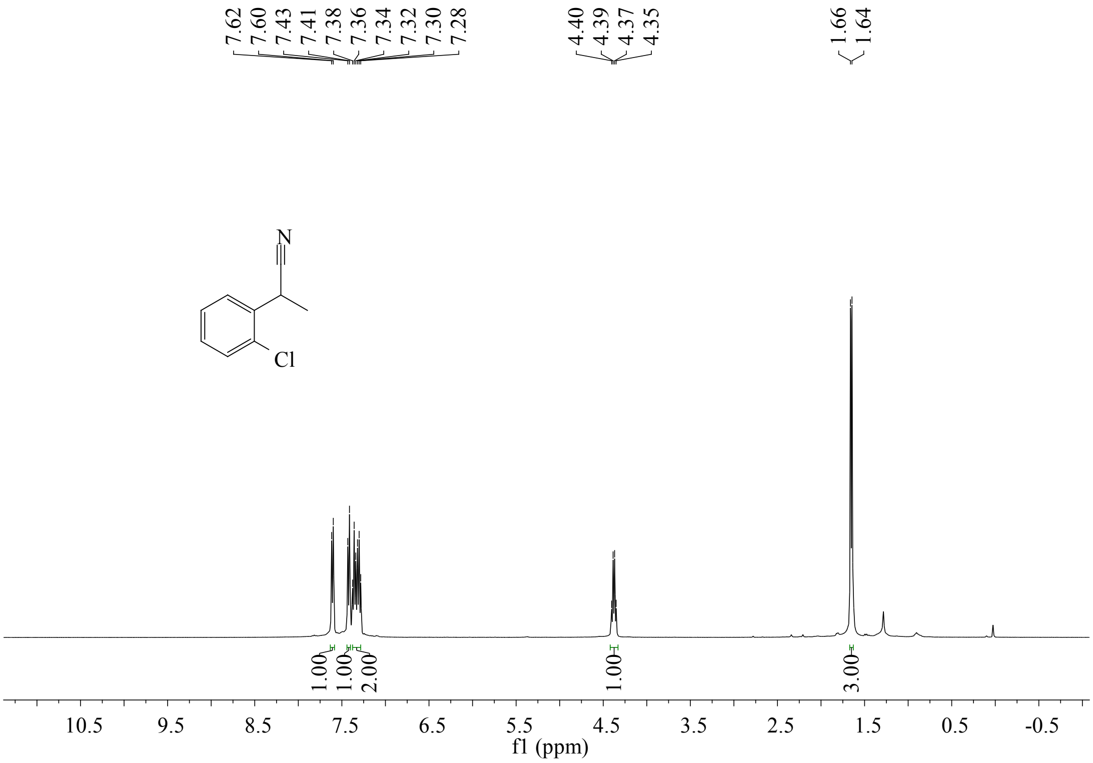

^{13}C NMR (101 MHz, CDCl$_3$) spectrum of compound 3va

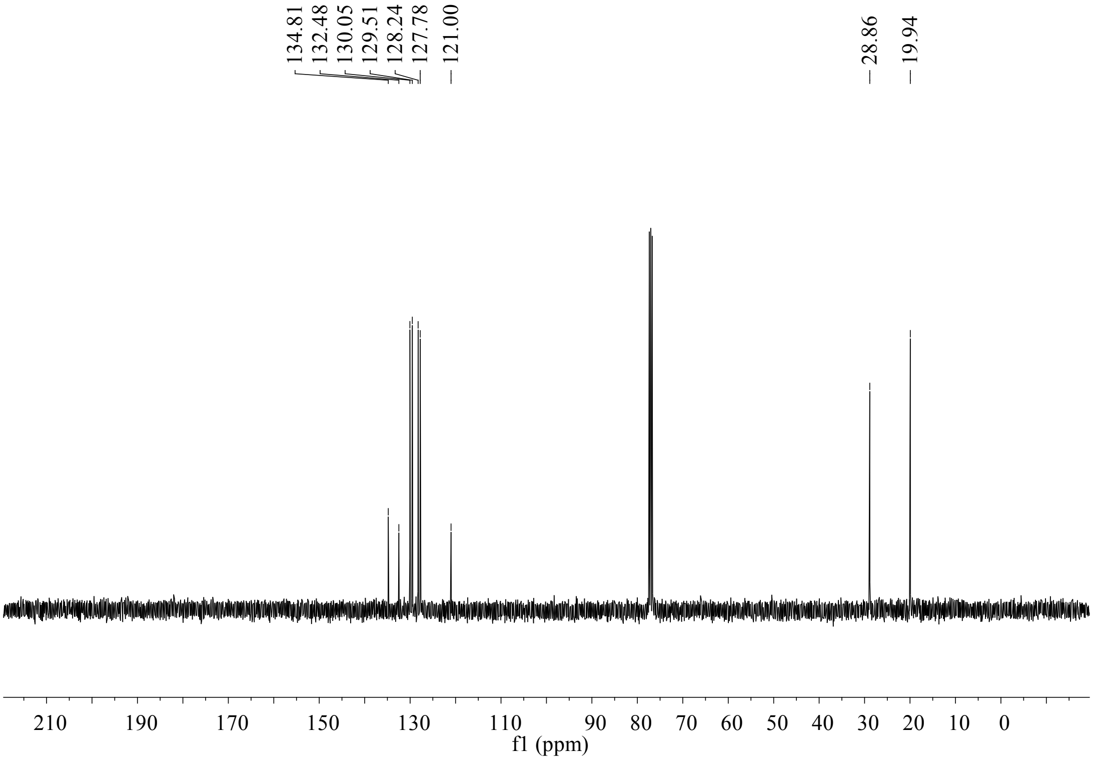

^{1}H NMR (400 MHz, CDCl$_3$) spectrum of compound 3wa

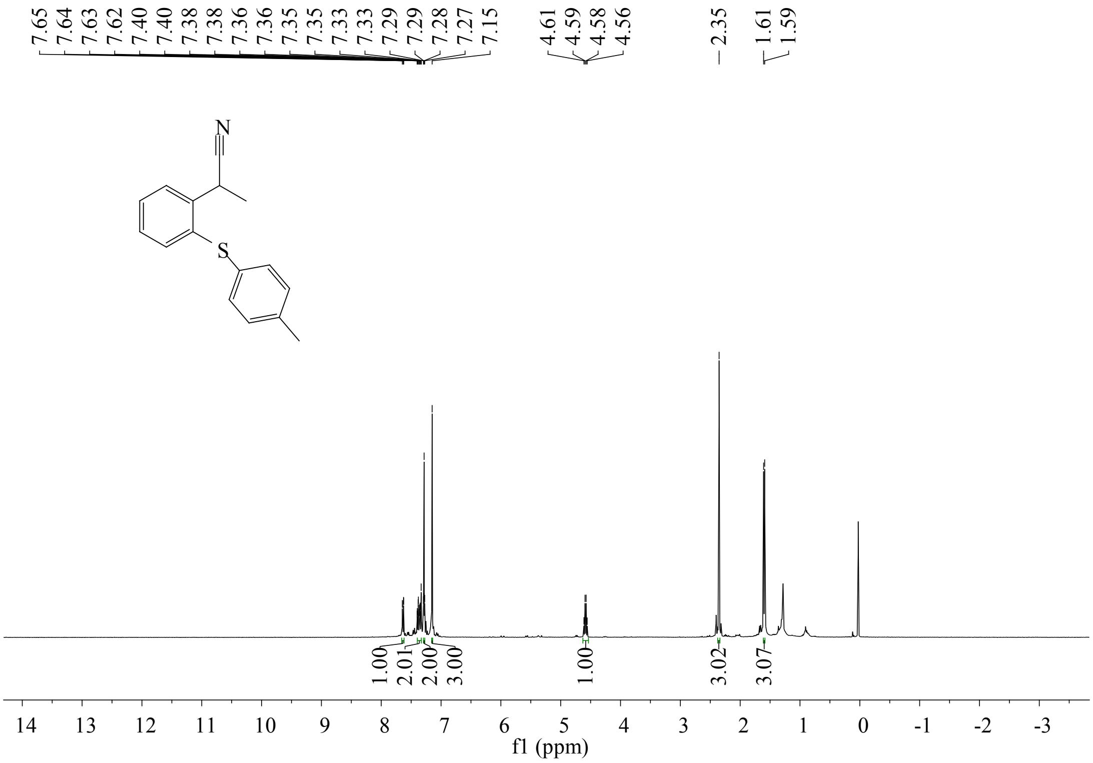

^{13}C NMR (101 MHz, CDCl$_3$) spectrum of compound 3wa

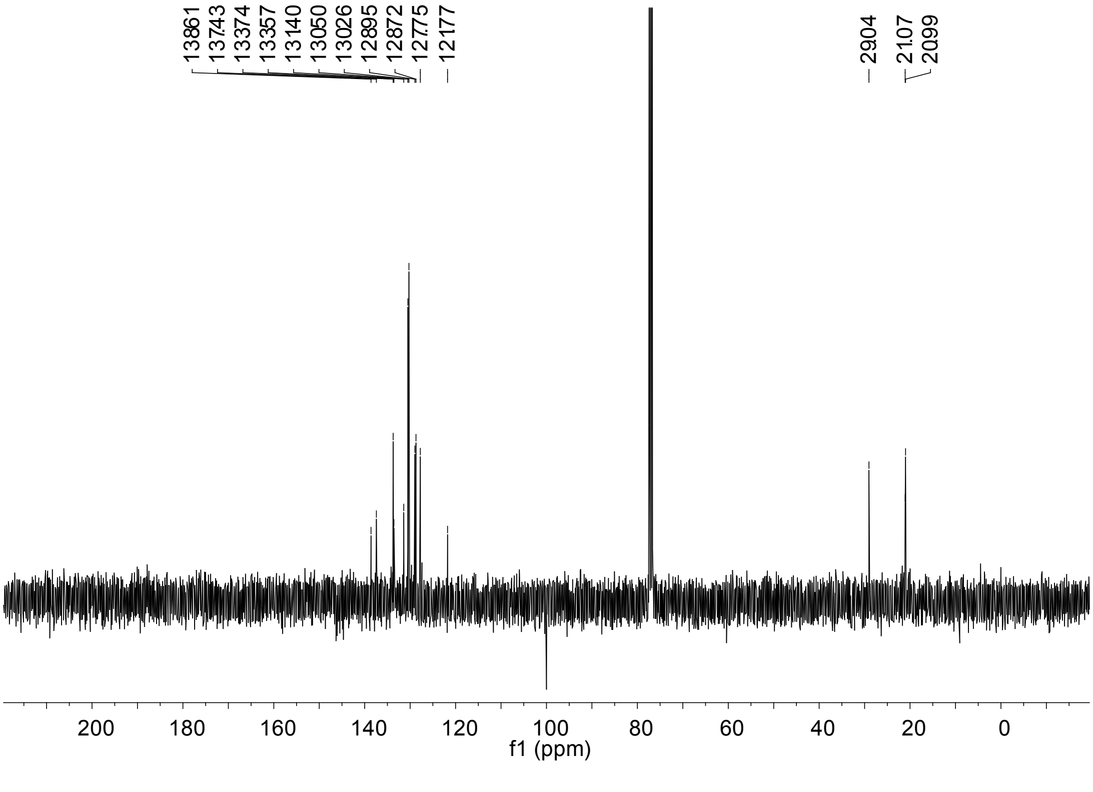

^{1}H NMR (400 MHz, $CDCl_3$) spectrum of compound 3xa

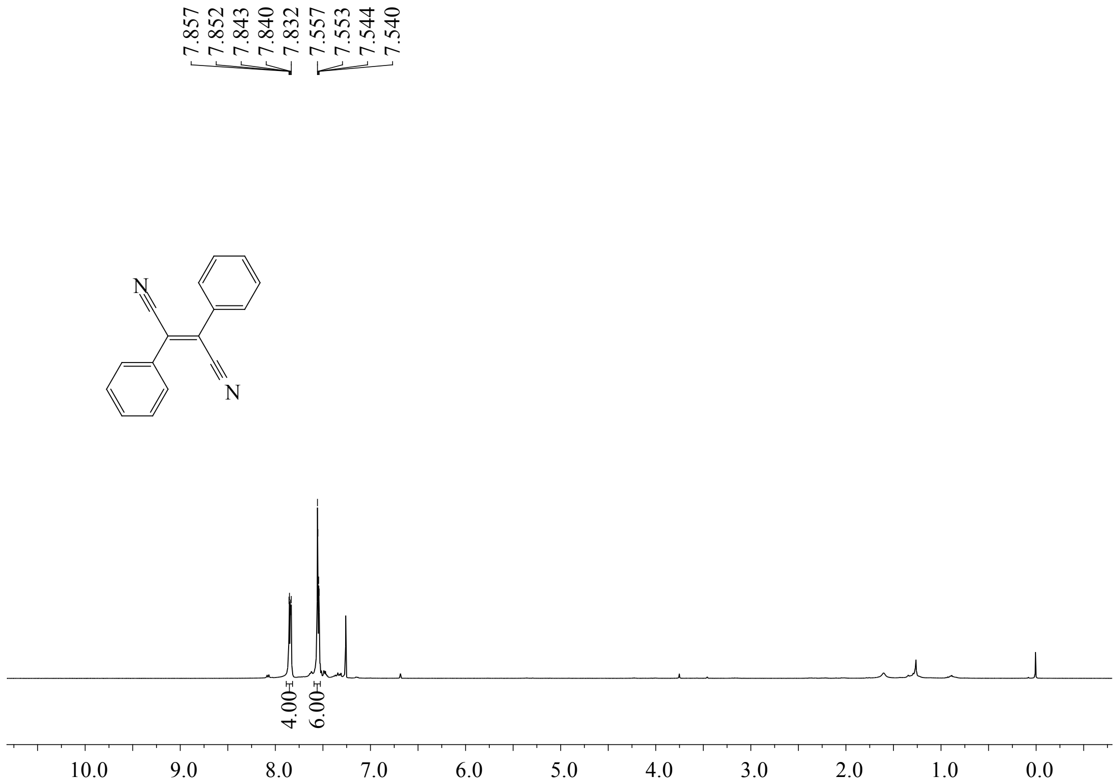

^{13}C NMR (101 MHz, $CDCl_3$) spectrum of compound 3xa

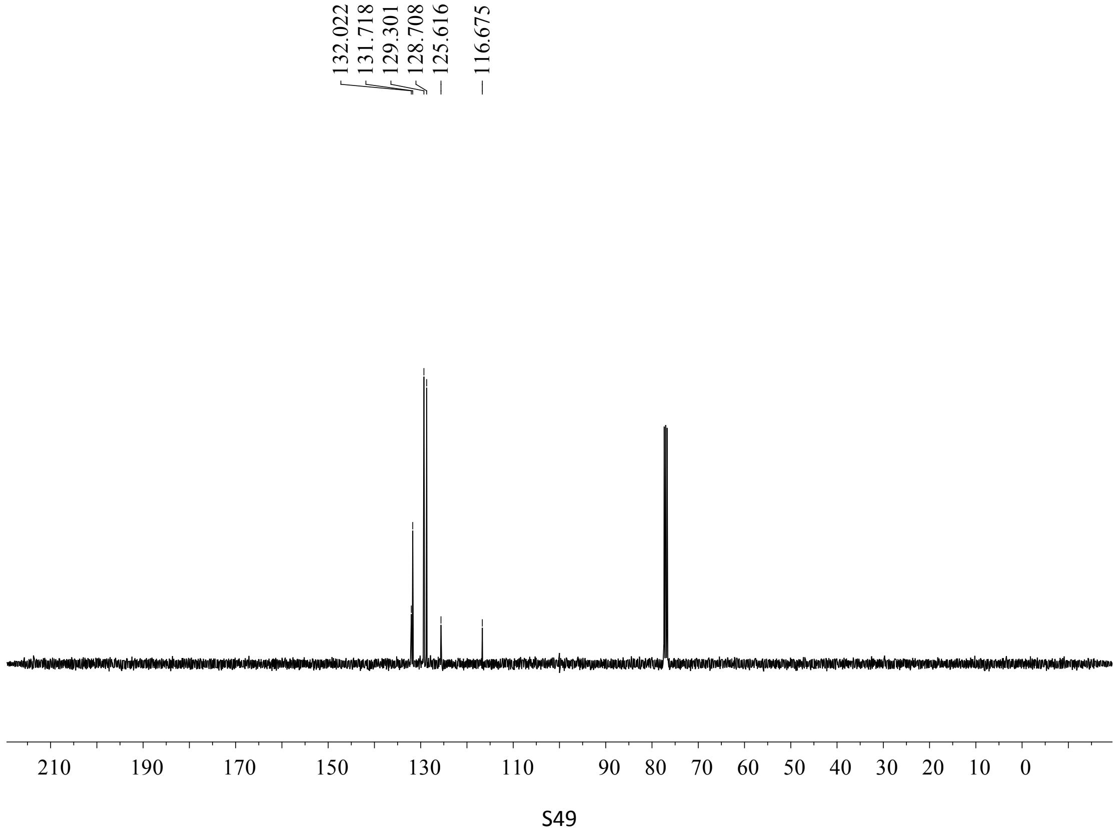

Supplement: Supplementary file 1 [file SC-008-C7SC02867A-s001.pdf]
